# Phthalocyanine-Cored Fluorophores with Fluorene-Containing Peripheral Two-Photon Antennae as Photosensitizers for Singlet Oxygen Generation

**DOI:** 10.3390/molecules25020239

**Published:** 2020-01-07

**Authors:** Seifallah Abid, Sarra Ben Hassine, Nicolas Richy, Franck Camerel, Bassem Jamoussi, Mireille Blanchard-Desce, Olivier Mongin, Frédéric Paul, Christine O. Paul-Roth

**Affiliations:** 1 CNRS, ISCR (Institut des Sciences Chimiques de Rennes)—UMR 6226, INSA Rennes, Univ Rennes, F-35000 Rennes, France; seifallah.abid@cemes.fr (S.A.); sarra.ben-hassine@insa-rennes.fr (S.B.H.); nicolas.richy@univ-rennes1.fr (N.R.); franck.camerel@univ-rennes1.fr (F.C.); olivier.mongin@univ-rennes1.fr (O.M.); frederic.paul@univ-rennes1.fr (F.P.); 2 Faculté des Sciences de Bizerte, Université de Carthage, 7021 Jarzouna, Tunisie; 3 Campus Universitaire Farhat Hached, Université de Tunis El Manar, B.P. n° 94-ROMMANA, Tunis 1068, Tunisie; 4 Department of Environmental Sciences, Faculty of Meteorology, Environment and Arid Land Agriculture, King Abdulaziz University, Jeddah 2798-22253, Saudi Arabia; bassem.jamoussi@gmail.com; 5 Institut des Sciences Moléculaires (CNRS UMR 5255), Univ. Bordeaux, 33405 Talence, France; mireille.blanchard-desce@u-bordeaux.fr

**Keywords:** fluorenyl, luminescence, oxygen sensitization, phthalocyanine, two-photon absorption

## Abstract

A series of free base and Zn(II) phthalocyanines featuring fluorenyl antennae linked by methoxy or oxo bridges to the phthalocyanine core (Pc) were synthesized and characterized. Selected linear and nonlinear (two-photon absorption) optical properties of these new compounds were subsequently studied. As previously observed for related porphyrin dendrimers bearing 2-fluorenyl peripheral dendrons, an efficient energy transfer occurs from the peripheral antennae to the central phthalocyanine core following excitation in the fluorenyl-based π–π* absorption band of these chromophores. Once excited, these compounds relax to the ground state, mostly by emitting intense red light or by undergoing intersystem crossing. As a result, the tetrafunctionalized Zn(II) phthalocyanines are fluorescent, but can also efficiently photosensitize molecular oxygen in tetrahydrofurane (THF), forming singlet oxygen with nearly comparable yields to bare Zn(II) phthalocyanine (**ZnPc**). In comparison with the latter complex, the positive role of the fluorenyl-containing antennae on one- and two-photon brightness (2PA) is presently demonstrated when appended in peripheral (β) position to the phthalocyanine core. Furthermore, when compared to known porphyrin analogues, the interest in replacing the porphyrin by a phthalocyanine as the central core to obtain more fluorescent two-photon oxygen photosensitizers is clearly established. As such, this contribution paves the way for the future development of innovative biphotonic photosensitizers usable in theranostics.

## 1. Introduction

**Figure 1:**
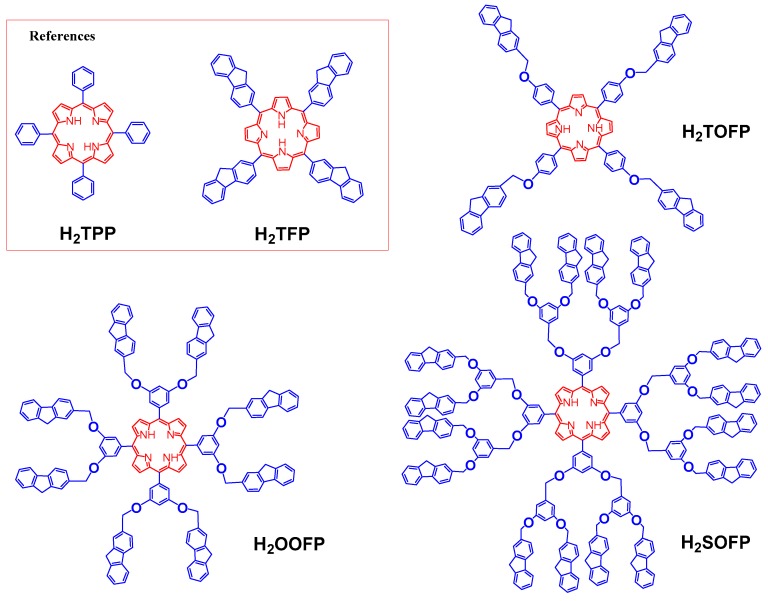
Series of porphyrin dendrimers previously studied and relevant reference compounds (**H_2_TPP** (tetraphenylporphyrin) and **H_2_TFP** (tetrafluorenylporphyrin)).

The chemistry of porphyrins and phthalocyanines involving new structural motifs and subunits, either within the central macrocycle or in its periphery, is in full development, in particular for photonic applications related to cancer therapy [1,2,3,4], such as photodynamic therapy (PDT) [2,5,6]. In this context, given the well-known oxygen photosensitizing properties of these macrocycles [7,8,9], studying new porphyrin- or phthalocyanine-based molecular assemblies that might efficiently undergo two-photon excitation [10,11,12,13] while presenting a significant luminescence, constitutes an appealing goal for researchers interested in developing new photosensitizers for theranostic applications [4,5,14,15,16]. Along these lines, some of us [17,18] and others [19], independently reported that fluorenyl units, when appended at the *meso* positions of porphyrins, were particularly suited to enhance the fluorescence quantum yields of this macrocyclic core, most likely by limiting the rate of non-radiative decay via internal conversion [20]. Thus, tetrafluorenylporphyrin (**H_2_TFP**) [21] features a quantum yield of 24% compared to that tetraphenylporphyrin (**H_2_TPP;** *Φ*_F_ = 12%) taken as a reference compound (Figure 1) [18]. Subsequent photophysical studies with tetra(fluorenylmethoxyphenyl)porphyrin (**H_2_TOFP**), octa(fluorenylmethoxy)tetraphenylporphyrin (**H_2_OOFP**), or hexadeca(fluorenylmethoxy)tetra-phenylporphyrin (**H_2_SOFP**) featuring four, eight [22] and sixteen peripheral fluorenyl groups [23], respectively, evidenced that 2-fluorenyl units were also able to enhance the luminescence quantum yield of the central tetraphenylporphyrin (**TPP**) core when appended by methoxy bridges to the *meso*-phenyl rings of a central **H_2_TPP** core (Figure 1). In these molecular assemblies, the fluorene groups play the role of light-harvesting “antennae”. Once photoexcited in their first π–π* state, these peripheral chromophores efficiently transfer their energy to the central macrocycle before emission, intersystem crossing, or other deactivation processes can take place. In line with Fréchet’s work [24], this “antenna effect” was stronger in dendritic assemblies such as **H_2_OOFP** or **H_2_SOFP** than in star-shaped architectures such as **H_2_TOFP**. As a result, new oxygen photosensitizers with significantly improved brightness over **H_2_TPP** could be obtained by these means [25]. Furthermore, the presence of several fluorenyl units at the periphery of the central macrocycle also allowed for a significant increase in the two-photon absorption (2PA) cross-section maximum at lowest energy, another very desirable feature when targeting PDT or theranostic applications in the near-infrared (NIR) range [14,15]. 

We can notice that, after having studied these non-conjugated TPP-based porphyrin dendrimers featuring flexible ether linkers (Figure 1), we considered more recently porphyrin dendrimers with conjugated peripheral arms [26]. Thus, a series of *meso*-substituted porphyrin dendrimers incorporating an increasing number of fluorenyl units, featuring fully conjugated alkynyl linkers, was studied. For these dendrimers, the 2,7-fluorenyl arms were connected directly to the *meso* positions of the central porphyrin core (i.e., featuring TFP core). Interestingly, in these assemblies, the conjugated peripheral arms incorporate fluorene units at both terminal and internal positions [26]. We also observed that the various fluorene-containing dendrons act as light-harvesting antennae, before transferring their energy to the central porphyrin core. Although the emission quantum yields are slightly diminished compared to that for the smaller **TFP** reference compound [20], we showed that their brightness was much improved [26]. 

**Figure 2:**
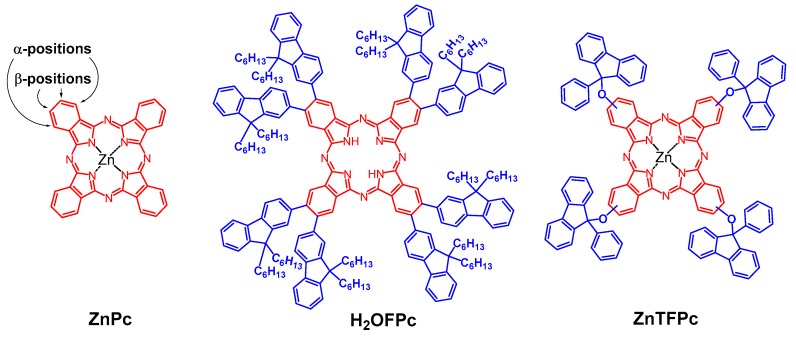
Zinc(II) phthalocyanine (**ZnPc**) (with ring-substitution positions indicated), free phthalocyanine octasubstituted by eight fluorenyl antennae (**H_2_OFPc)**, and zinc(II) complex of a phthalocyanine tetrasubstituted by four fluorenyl antennae at β-positions (**ZnTFPc**).

Following our initial goal to further improve the luminescence quantum yield of the photosensitizers with flexible arms [22,23,25], we now wondered about the impact of replacing the porphyrin central core by a phthalocyanine ring. Based on literature reports, this tetrapyrrolic unit should be able to photosensitize oxygen with an efficiency similar to that of the *meso*-tetraarylporphyrin core once metalated by Zn(II) [27], but should also present a significantly improved fluorescence quantum yield [7]. Thus, a fluorescence quantum yield of 30% was reported for **ZnPc** at 77 K [28], which decreased to 23% in THF at ambient temperature [29], making Zn(II) phthalocyanines an appealing alternative to porphyrins in such molecular assemblies. So far, free base or Zn(II) phthalocyanines substituted by fluorenyl-containing arms are rare in the literature [30,31]. The few extant examples (Figure 2) are more fluorescent than **H_2_TFP** (*Φ*_F_ = 22%) or **ZnTFP** (*Φ*_F_ = 5%) [18], but there is room for improvement. Indeed, the solubility of fully planar compounds, prone to undergoing stacking interactions, is often an issue for their practical use in solution. 

Thus, in spite of its remarkably high fluorescence quantum yield (*Φ*_F_ = 88%) [30], **H_2_OFPc** (Figure 2) suffers from a poorly efficient energy transfer (ET) from the fluorenyl-based antennae toward the central macrocycle, possibly due to the unfavorable conformations adopted by the peripheral antennae relative to the central phthalocyanine core, these conformations being likely induced by steric strain. This steric congestion results from octasubstitution at the β-positions and from the long *n*-hexyl chains at the nine positions of the 2-fluorenyl units, the latter being required to solubilize this compound. In this respect, tetrasubstitution at the α- or β-positions by 2-methoxyfluorenyl units might be more favorable to promote energy transfer while maintaining solubility. Indeed, Hanack and coworkers showed that phthalocyanines substituted at the peripheral positions solely by alkyl or alkyloxy groups present usually fair to good solubilities in common organic solvents, and that tetrasubstituted phthalocyanines are in general more soluble than octasubstituted derivatives [32]. For instance, the regioisomers of the known **ZnTFPc** (Figure 2) do not require the presence of additional alkyl chains to be solubilized in organic solvents [31]. 

**Figure 3:**
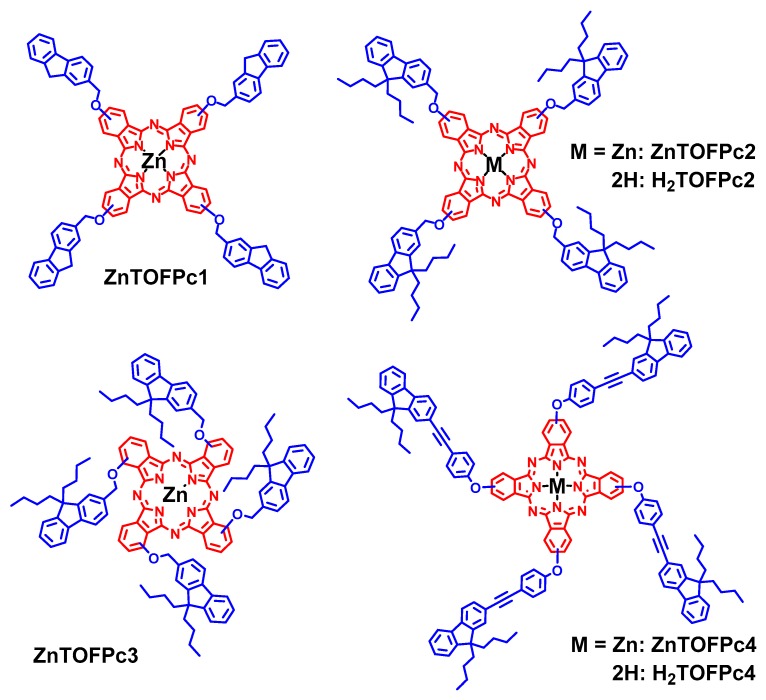
New targeted alkoxy and phenoxy substituted phthalocyanines.

Based on these observations, we have now decided to synthesize and characterize a series of new tetrasubstituted phthalocyanines featuring 2-fluorenyl groups as surrounding antennae (Figure 3). This series is composed of three phthalocyanines peripherically substituted at one β-position on each benzopyrrole unit (**ZnTOFPc1**, **ZnTOFPc2**, and **ZnTOFPc4**) and one analogue tetrasubstituted at α-positions (**ZnTOFPc3**). The fluorescence quantum yields, oxygen photosensitization yields, and two-photon absorption (2PA) cross-section maxima will then be experimentally determined for these new compounds. The comparison between **ZnTOFPc1** and **ZnTOFPc2** should allow pointing out (i) the influence of the *n*-butyl groups on the nine positions of the fluorene units, while the comparison between **ZnTOFPc2** and **ZnTOFPc3** should provide information about (ii) the influence of the substitution site on the phthalocyanine core. Finally, the comparison between **ZnTOFPc2** and **ZnTOFPc4** should provide some information about the impact of (iii) the *π*-manifold extension on the peripheral arms in compounds featuring phenoxy spacers. Indeed, like carbonyl or 1,1′-alkene spacers, [33,34] oxygen atoms should allow for n–π “cross-conjugation” [35], potentially mediating an interaction between the *π*-manifolds located on each side of the oxygen atoms in compounds such as **ZnTOFPc2**. In addition, (iv) the role of the metal center on the optical properties might be inferred by comparing the data gathered for the two last examples from their corresponding free bases (**H_2_TOFPc2** and **H_2_TOFPc4**). With the help of density functional theory (DFT) calculations, the impact of these structural changes on the optical properties of interest will be better rationalized, and the potential of these new compounds relative to free-base porphyrin analogues such as **H_2_TOFP**, **H_2_OOFP**, or **H_2_SOFP** for fluorescence imaging or PDT will be evaluated.

## 2. Results

The targeted phthalocyanines were obtained via the most common synthetic approach: thermal condensation of the corresponding phthalonitrile precursors [36]. The latter (**Pn1**–**4**) were obtained by nucleophilic substitution of the nitrophthalonitrile precursors **1** or **2** by the corresponding alcohols, which are readily available in a few steps from commercial products [37,38]. 

### 2.1. Synthesis of the Phthalonitrile Precursors

**Scheme 1:**
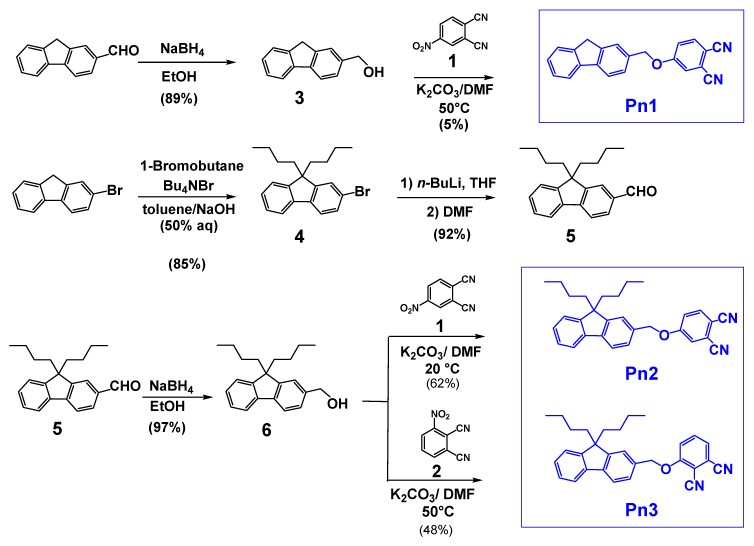
Synthesis of phthalonitriles **Pn1**–**3.**

The synthesis of the alkoxy phthalonitriles **Pn1**–**3** is detailed in Scheme 1. Whereas **Pn1** was isolated in low overall yield from **1** and fluorenaldehyde (ca. 4%), better overall yields were obtained for **Pn2** (47%) and **Pn3** (36%) from **1** or **2**, respectively, and commercial 2-bromofluorene. For **Pn1** and **Pn3**, heating at 50 °C was essential to optimize the yields.

**Scheme 2:**
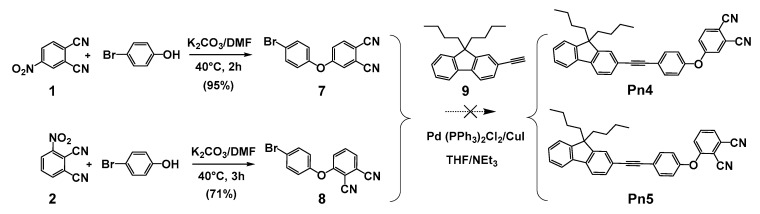
Unfruitful synthetic assays for isolating phthalonitriles **Pn4** and **Pn5.**

To synthesize the new aryloxy phthalonitriles **Pn4** and **Pn5**, we firstly tried the reaction sequence depicted in Scheme 2 [1], but this approach did not lead to the desired compounds.

**Scheme 3:**
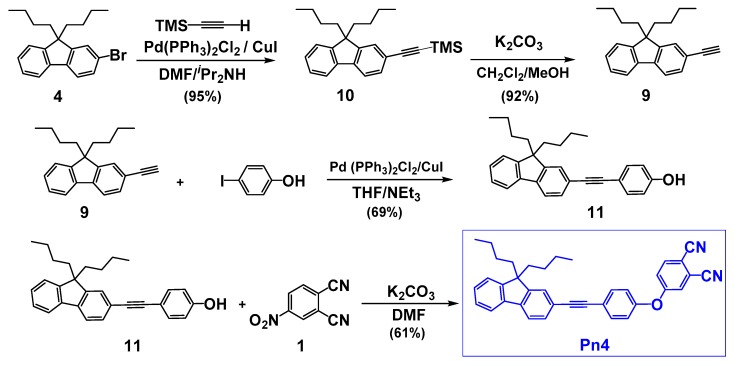
Synthesis of phthalonitrile **Pn4** featuring an extended fluorene-containing arm.

**Pn4** was finally obtained via another synthetic route (Scheme 3) in modest overall yield (31%) from **1** and commercial bromofluorene (**4**) [39], whereas **Pn5** could not be obtained via this route.

**Figure 4:**
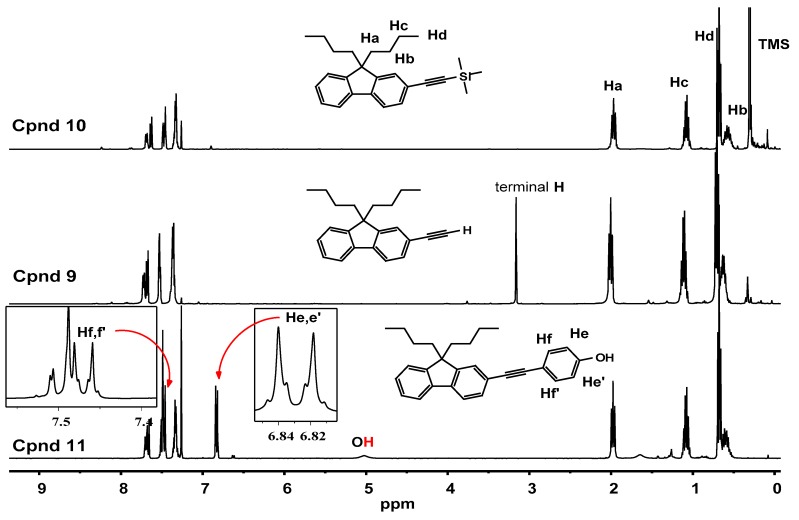
^1^H-NMR spectra of the fluorene-containing precursors **10**, **9**, and **11** in CDCl_3_.

**Figure 5:**
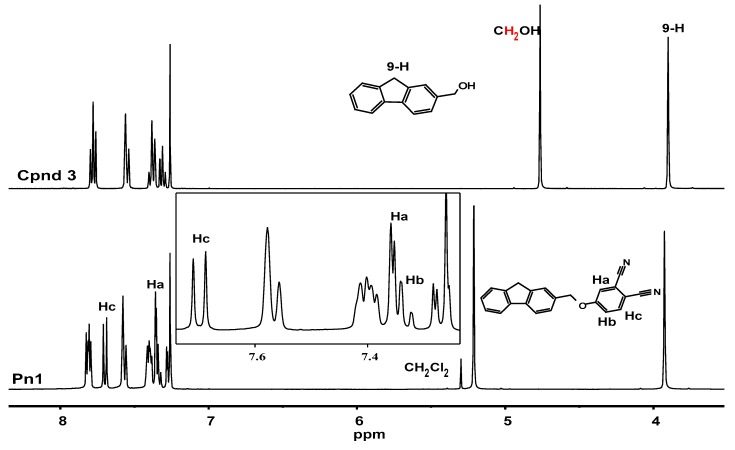
Comparison of the ^1^H-NMR spectra of the precursor **3** and **Pn1** in CDCl_3_.

The various fluorene precursors (**3**–**9**), as well as the phthalonitriles **Pn1**–**4**, were conveniently characterized by ^1^H-NMR (Figures S1–S13, Supplementary Materials). This spectroscopy can even be used to monitor the various reactions using diagnostic signals. Thus, the intense singlet toward 0 ppm corresponding to the nine protons of the trimethylsilane (TMS) group in ethynylfluorene (**10**) disappears after deprotection and is replaced by a singlet at 3.2 ppm corresponding to the alkyne proton of **9**, while the aromatic protons (between 7.3 and 7.7 ppm) remain fairly similar for compounds **7** and **8** (Figure 4) [40]. For the alcohol **11**, the alkynyl proton peak at 3.2 ppm disappears while additional signals corresponding to a *para*-phenol group appear (i.e., an AA’XX’-system H_e_, H_e’_, H_f_, H_f’_, and a broad hydroxy peak, see Figure 5). For all these compounds, the *n*-butyl chains at the nine positions of fluorenyl groups are clearly identified by four multiplets between 0 and 2 ppm corresponding to H_a_, H_b_, H_c_, and H_d_, and the OH proton appears as a large singlet peak at about 5 ppm (Figure 4, bottom).

The phthalonitriles **Pn1**–**4** also exhibited diagnostic ^1^H-NMR signatures. When compared to their alcoholic precursors **3**, **6**, and **11**, their formation was clearly established by the appearance of three new peaks in the aromatic region (7–8 ppm), corresponding to the phthalonitrile protons H_a_, H_b_, and H_c_ (as exemplified for **Pn1** in the inset of Figure 5). A strong downfield shift of the methylenic protons, which appear as an intense singlet at 5.2 ppm, could also be noticed for **Pn1**–**3**. For **Pn1**, by comparison, the two protons at position nine on the fluorene are not shifted and appear at a similar shift as in precursor **3**, i.e., around 3.9 ppm (9-H).

### 2.2. Synthesis of Phthalocyanine Macrocycles

**Scheme 4:**
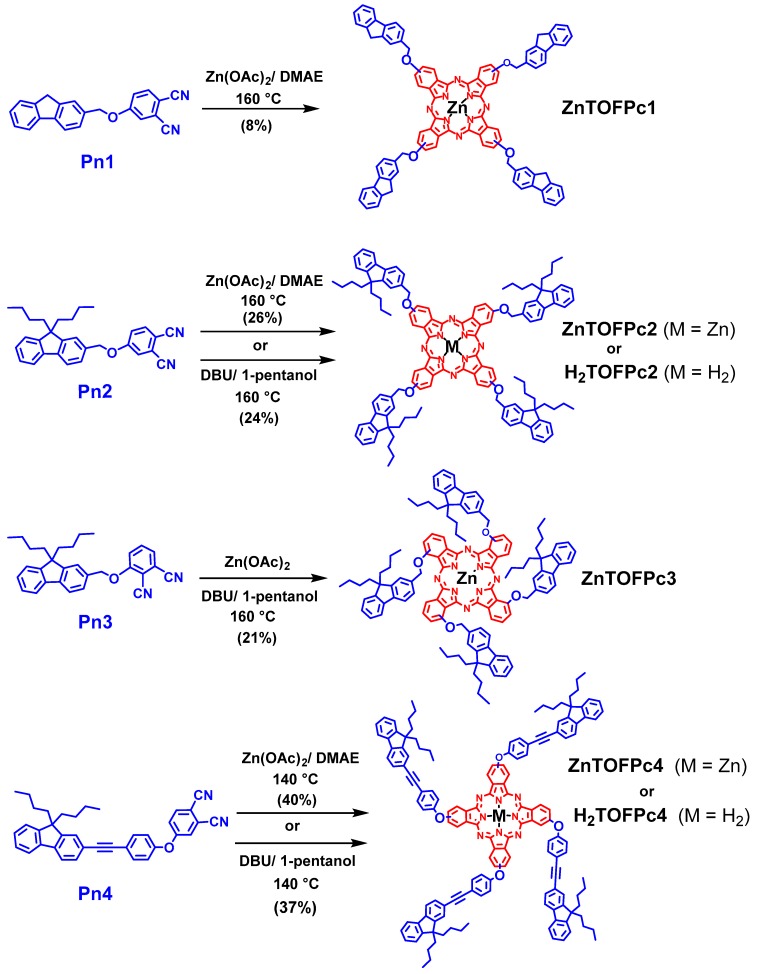
Synthesis of zinc(II) complexes **ZnTOFPc1**–**4** and free bases **H_2_TOFPc2** and **H_2_TOFPc4**.

The synthesis of the targeted phthalocyanines was then performed from **Pn1**–**4** precursors (Scheme 4). 

**Figure 6:**
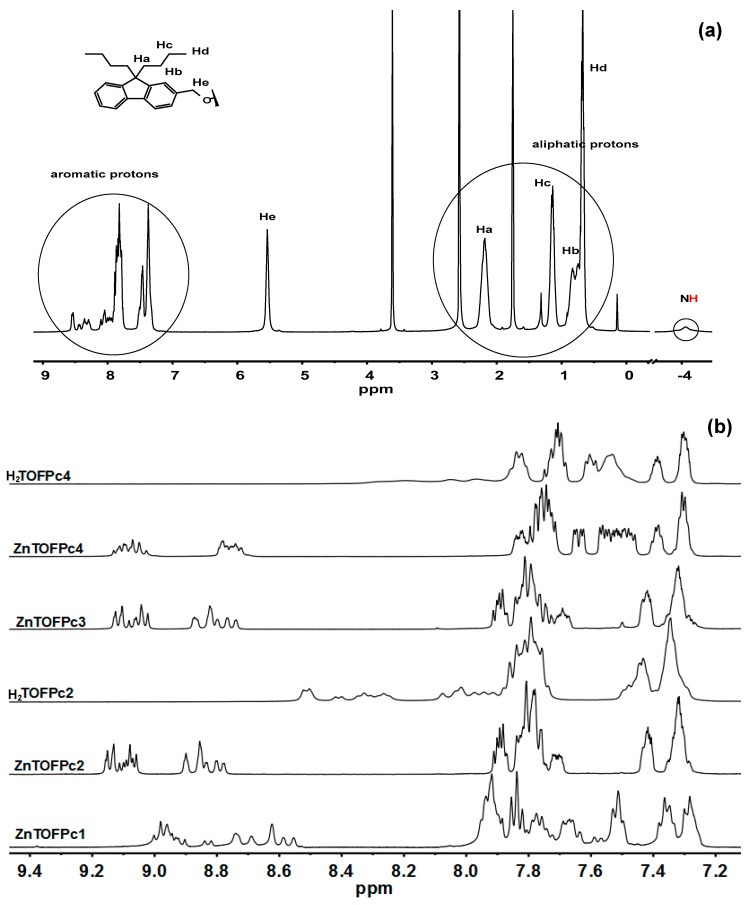
^1^H-NMR in THF-d_8_: (**a**) Complete spectrum of **H_2_TOFPc2**. (**b**) Aromatic part of the ^1^H-NMR spectra of **ZnTOFPc1**–**4** complexes and free **H_2_TOFPc2** and **H_2_TOFPc4**, showing the presence of several positional isomers.

The Zn(II) complexes **ZnTOFPc1**–**2** and **ZnTOFPc4** were obtained by heating the corresponding phthalonitriles with zinc acetate in dimethylaminoethanol (DMAE), while the free bases **H_2_TOFPc2** and **H_2_TOFPc4** were isolated by heating their phthalonitrile precursors in pentanol, in the presence of 1,8-diazabycyclo[5.4.0]undec-7-ene (DBU), following an approach reported in the literature [36,41]. The zinc phthalocyanine complex **ZnTOFPc3**, which could not be obtained following the conditions used for isolating the other Zn(II) derivatives, could be isolated via this second approach in presence of zinc acetate. Except for **H_2_TOFPc4** and **ZnTOFPc4**, all these condensation reactions were carried out at 160 °C in order to solubilize the various phthalonitrile precursors in the solvents used. These new phthalocyanines were purified by chromatography and recrystallized from dichloromethane/methanol. As confirmed by ^1^H-NMR (Figure 6 and Supplementary Materials; Figure S14–S24), all compounds were isolated as a mixture of regioisomers [32,42]. Following an established practice [16,31,43,44], no further attempt was made to separate these isomers by chromatography, and the products were characterized as mixtures [42,45]. 

The yields of the various phthalocyanines isolated after these reactions varied widely from one phthalonitrile precursor to another, an outcome most likely resulting from their different solubilities. For example, the unsubstituted **Pn1**, used directly for the synthesis of **ZnTOFPc1**, had a very low solubility in DMAE, even at high temperature. As a result, the isolated yield of **ZnTOFPc1** reached only 8% after chromatographic separation. However, better yields were obtained for other derivatives featuring *n*-butyl chains at the ninth position of the fluorene group. Quite likely, the better solubility of their phthalonitrile precursors facilitated their reaction, while the enhanced solubility of the resulting phthalocyanines simplified their purification by chromatography. In this respect, the two butyl chains at the ninth position of the peripheral fluorenyl groups significantly improved the solubility of **ZnTOFPc2** in common organic solvents compared to **ZnTOFPc1**. As a result, the former compound was amenable to ^13^C-NMR characterization, whereas the latter was not soluble enough for these studies.

In general, the low solubility of phthalocyanines, which results from their tendency to aggregate in solution, is an obstacle to their characterization and practical use. Aggregation takes usually place via strong π–π interactions between some of the condensed aromatic rings of different molecules [44]. To study aggregation in solution, ultraviolet-visible (UV–Vis) [31,44] and NMR [46,47] spectroscopies are commonly used. These two techniques provide information on the formation of dimers, trimers, and higher aggregates, and can also give information on the conformation of dimeric aggregates in same cases [48]. Presently, we could show that the chemical shifts of the protons depended both on the concentration and on the temperature [49,50]. Thus, the ^1^H-NMR spectra of the zinc complex **ZnTOFPc2** at different concentrations (C ≈ 6, 14, and 22 mM) revealed a doubling of the characteristic methylene (CH_2_O) peak upon increasing concentrations (Figure S24, Supplementary Materials), presumably due to dimer formation. This chemical shift underwent a change up to 0.6 ppm in line with *π*-stacking. Most likely, these protons feel the shielding effect of aromatic rings from aggregated partner molecules [50,51]. 

In spite of this self-aggregation process, the phthalocyanines **ZnTOFPc1**–**4**, **H_2_TOFPc2,** and **H_2_TOFPc4** remained soluble enough for ^1^H-NMR characterization at lower concentrations (below 10 mM), before aggregation started, as illustrated with **TOFPc2** (Figure 6a). The singlet H_e_ at 5.5 ppm corresponds to the eight protons of the four –CH_2_O– units of the fluorenyl pendant arms. The protons of the alkyl chains (H_a_–H_d_) appear between 0.5 and 2.5 ppm and the two NH protons of the macrocycle core are observed at stronger field (−4 ppm). For the aromatic part, the complexity of the spectrum reveals the presence of several positional isomers in solution, a feature not apparent for the other signals. We can also notice (Figure 6b) that the influence of the ring current is stronger for the four zinc complexes **ZnTOFPc1**–**4** in the aromatic region than for free phthalocyanines. The latter resulted in a downfield shift of the phthalocyanine aromatic protons ca. 1 ppm, more pronounced in the former case.

### 2.3. Photophysical Properties

One- and two-photon absorption and emission properties, as well as oxygen photosensitization properties, were next determined for **ZnTOFPc1**–**4**, **H_2_TOFPc2,** and **H_2_TOFPc4** in THF solution (Table 1). The known **ZnPc** was used as a model complex to study the influence on the optical properties of the various antennae appended at α- and β-positions to the phthalocyanine core.

### 2.4. Absorption Properties

**Figure 7:**
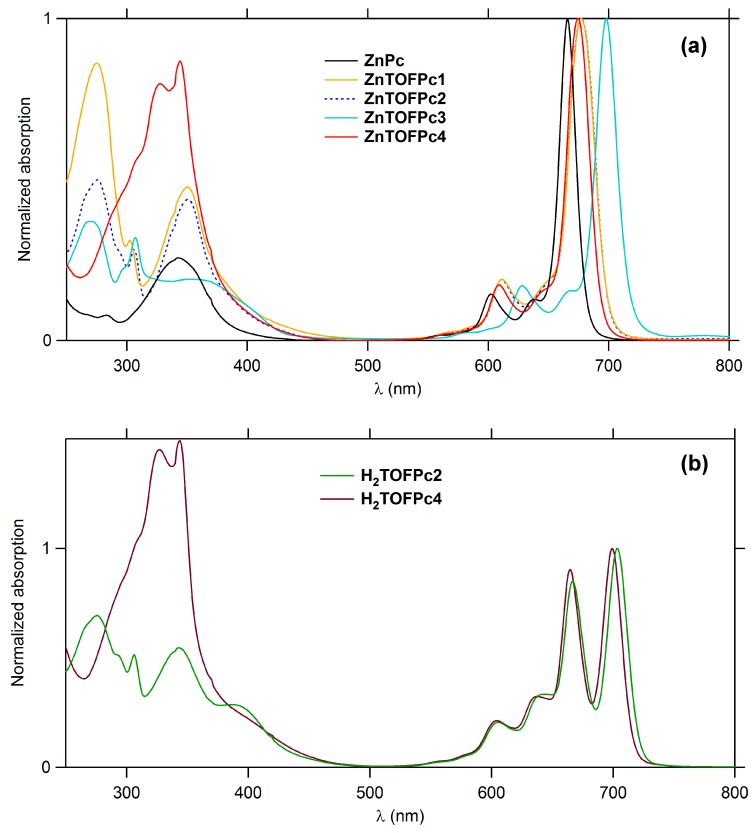
UV/Vis absorption spectra of the various phthalocyanine derivatives in THF at 20 °C: (**a**) **ZnTOFPc1**–**4**, and **ZnPc** used as reference; (**b**) **H_2_TOFPc2** and **H_2_TOFPc4**.

The UV/Vis absorption spectra of all these phthalocyanine derivatives were recorded between 250 and 800 nm (Figure 7a,b). The absorption spectra of the zinc phthalocyanine featured three characteristic types of bands: (i) an intense absorption band between 650 and 750 nm corresponding to π–π* transitions from the highest occupied molecular orbital (HOMO) toward the lowest vacant molecular orbitals (LUMO and LUMO + 1), called the Q band, (ii) another intense band, between 300 and 400 nm, corresponding to π–π* transitions from deeper-lying molecular orbitals toward the LUMO and LUMO + 1, called the Soret band (or B band), and a less intense and much narrower band, around 280–300 nm, called the N band, corresponding to more energetic π–π* transitions [52]. In line with ^1^H-NMR findings, the shape (absence of shoulders) and half-width of these bands are indicative of non-aggregated phthalocyanines [29,44]. In addition to these three characteristic absorption bands, we could also observe another strong band between 250 and 400 nm, attributable to fluorene-based π–π* transitions originating from the peripheral antennae (Figure 7a) [22,23]. In **ZnTOFPc4** and **H_2_TOFPc4**, due to the extended conjugation of the peripheral arms, this absorption band is bathochromically shifted and overlapped with the Soret and N bands.

The zinc(II) complexes are characterized by a main intense Q band around 700 nm, whereas a splitting of this band is observed for free bases **H_2_TOFPc2** and **H_2_TOFPc4** due to the loss of symmetry induced by the presence of the two central NHs (Figure 7b and Table 1) [52]. This splitting results in a significant red shift of the first Q-band absorption for the free bases compared to the corresponding Zn(II) complexes. The characteristic absorptions for the new Zn(II) phthalocyanines are all bathochromically shifted compared to **ZnPc** used as a reference, particularly regarding the Q band. Notably, this shift is more important for the α-substituted complex **ZnTOFPc3** than for its β-substituted analogue **ZnTOFPc1**. The former presents a maximum at 698 nm for the Q band (i.e., with a red shift of 688 cm^−1^ compared to **ZnPc**). Along the same lines, comparing the spectra of **ZnTOFPc1** and **ZnTOFPc2** reveals that the introduction of *n*-butyl chains at the nine positions of the fluorene groups has no effect on the B- and Q-band energies.

### 2.5. Emission Properties

**Figure 8:**
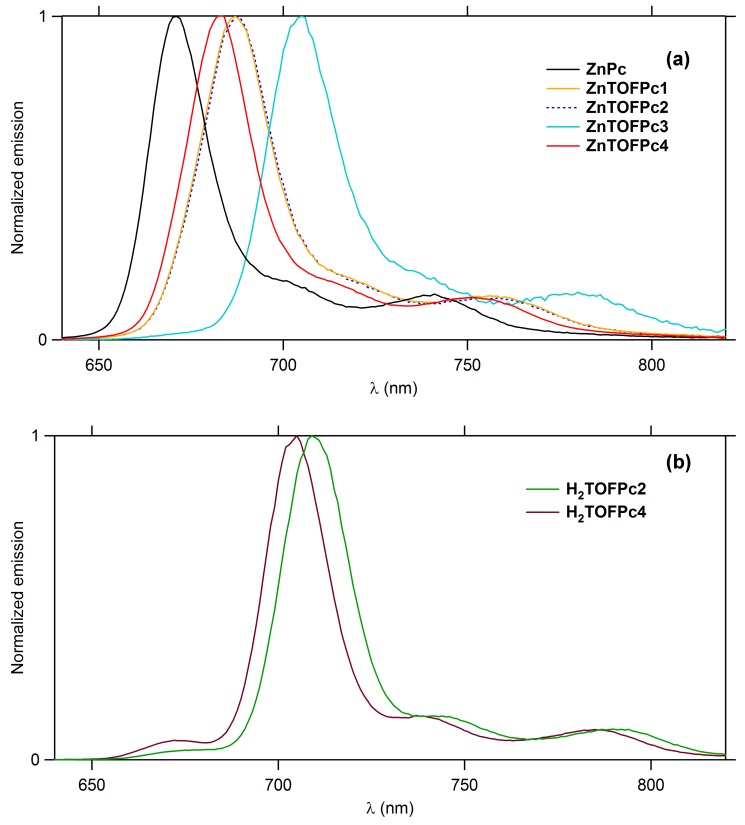
Emission spectra of the various phthalocyanine derivatives in THF at 20 °C (excitation at 606 nm): (**a**) **ZnTOFPc1**–**4** and **ZnPc** used as reference; (**b**) **H_2_TOFPc2** and **H_2_TOFPc4**.

Upon excitation in their first Q band at 606 nm, all these compounds are characterized by a strong red emission in solution, between 650 and 800 nm (Table 1). The emission spectra of the Zn(II) complexes always present the same profile (Figure 8a), i.e., a very intense Q(0,0) band followed by a less intense Q(1,0) band at lower energy, mirroring the Q-band absorption profile. In contrast, four Q bands instead of two are observed for the free bases **H_2_TOFPc2** and **H_2_TOFPc4** (Figure 8b), with a maximum at higher wavelengths than that of the corresponding zinc complexes (i.e., corresponding to a red shift of ca. 24–26 nm or 509–546 cm^−1^). Compared to **ZnPc**, the emission bands of the Zn(II) derivatives are bathochromically shifted, with the α-substituted derivative (**ZnTOFPc3**) being the most red-shifted among them. The Stokes shifts are slightly larger for the Zn(II) complexes (200 ± 40 cm^−1^) than for the free bases (ca. 120 ± 2 cm^−1^), but these remained overall quite weak, indicative of a modest structural relaxation taking place in the Q(0,0) state [52]. 

The fluorescence quantum yields of these compounds were then determined by comparison with **ZnPc** in a toluene/pyridine (99:1) solution. In this mixture, the complex **ZnPc** is axially coordinated by a pyridyl ligand to form the **(Py)ZnPc** adduct. Compared to this standard, we firstly determined the quantum yield of **ZnPc** in THF. A value of 26% was found, in fair agreement with the literature value of 23%, which was determined using a different standard (chlorophyll in diethylether) [29]. The quantum yields of the other compounds were then determined and showed significant differences. The free bases are always more luminescent than the corresponding Zn(II) complexes [43], in line with the absence of the heavy-metal atom effect. The latter usually favors intersystem crossing at the expense of fluorescence [28,54]. Then, all Zn(II) complexes substituted at the β-positions were shown to present a higher fluorescence yield than **ZnPc**, while the α-substituted derivative **ZnTOFPc3** had a slightly lower yield (23%). The relatively lower fluorescence quantum yield of **ZnTOFPc2** compared to **ZnTOFPc1** (featuring two butyl chains) might be traced back to the existence of additional *ν*(C–H) modes, providing perhaps additional non-radiative decay pathways.

Finally, the one-photon brightness (*ε*.*Φ*_F_), an important figure of merit for fluorescence imaging [55], was determined for all these compounds using the Q-band absorption coefficients (*ε*_max_). The Zn(II) complexes were found to be brighter than their free bases upon excitation in their Q band, in spite of lower fluorescence quantum yields. This was due to the symmetry change of the phthalocyanine core upon metalation by Zn(II). As a result, the two non-degenerate Q bands of the free base merged into a single band in the complex, resulting in an increase in intensity of the first Q band. The brightest of these Zn(II) phthalocyanines was **ZnTOFPc4**. 

### 2.6. Energy Transfer from the Fluorene Units to the Phthalocyanine Core

The existence of an energy transfer (ET) mechanism between the peripheral dendrons and the central phthalocyanine core was probed by specifically exciting the fluorene-based π–π* transition of the dendrons (around 270 or 320 nm) for **ZnTOFPc1**–**4**, **H_2_TOFPc2**, and **H_2_TOFPc4** (Figure S25, Supplementary Materials). Only the red emission between 650 and 800 nm, characteristic of the phthalocyanine core, was detected, with similar fluorescence quantum yields to when these fluorophores were excited in their Q absorption band (606 nm). This observation was indicative of a nearly quantitative (*Ф*_ET_ ~ 100%) energy transfer from the peripheral fluorene units to the central macrocyclic core for all these compounds.

### 2.7. Oxygen Photosensitization

The quantum yields of singlet oxygen generation were then determined for these phthalocyanine complexes (**ZnTOFPc2**–**4**) and for **ZnPc**, used as reference, in a toluene/pyridine (99:1) mixture, using singlet oxygen luminescence detection (Table 1) [54]. Comparison between these complexes showed that all these phthalocyanines have a strong capacity to produce singlet oxygen upon photoexcitation. Albeit slightly lower than that of ZnPc, *Φ*_Δ_ values of 57%, 60%, and 54% were found, respectively (Table 1). These *Φ*_Δ_ values are in the range usually reported for Zn(II) phthalocyanines [27,29,44,54,56]. Upon comparison with the fluorescence quantum yields of these compounds, it appears that the increase in fluorescence efficiency stated for these fluorophores was only partly obtained at the expense of the singlet oxygen production. Considering that the quantum yield for intersystem crossing to the triplet state is usually (marginally) higher than the oxygen photosensitization yield [44,54,56], this means that internal conversion or other (less interesting) decay processes were minimal in those compounds.

### 2.8. Two-Photon Absorption

**Figure 9:**
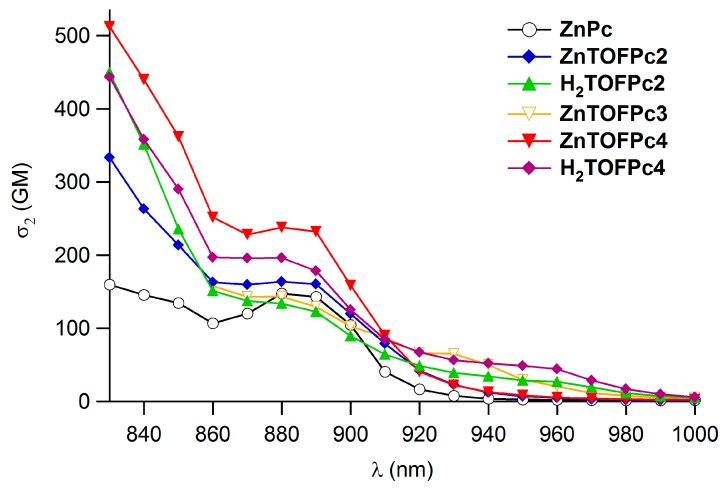
Two-photon absorption spectra for the various phthalocyanines in THF at 20 °C.

As the new tetrasubstituted phthalocyanines exhibited good fluorescence properties, their two-photon absorption cross-sections were determined by two-photon excited fluorescence (TPEF) in THF (Figure 9 and Table 2). Measurements were performed with 10^−4^ M solutions, using a mode-locked titanium–sapphire laser delivering femtosecond pulses, following the experimental protocol described by Xu and Webb [57]. A totally quadratic dependence of the fluorescence intensity as a function of the laser excitation power was observed for each compound at all wavelengths (Figure S26, Supplementary Materials), indicating that the measured cross-sections are solely due to pure two-photon absorption (intrinsic *σ*_2_) [10].

Likewise to what was observed with related free base porphyrin derivatives such as **H_2_OOFP** or **H_2_SOFP** (Figure 1) [25], the cross-sections increased toward higher energies (lower wavelengths) until a limit below which residual one-photon absorption started taking place, i.e., 840 nm for **ZnTOFPc1**, 830 nm for **ZnTOFPc2** and **ZnTOFPc4**, and 860 nm for **ZnTOFPc3**. A first (less intense) 2PA maximum was also observed at lower energies, around 880 nm, for all compounds. Whereas the second maximum might be related to an excited state located within the Soret band, the first one cannot be related to an allowed one-photon excited state, since no absorption band was observed around 440 nm, a spectral region located within the tail of the Soret band. These results are in perfect agreement with those previously reported by Rebane and coworkers for Zn(II) phthalocyanines in pyridine [58]. The first maxima is attributed to a transition into a one-photon forbidden *g*–*g* excited state, whereas the increase of the 2PA curve at shorter wavelengths is attributed to a second 2PA transition into another one-photon forbidden *g*–*g* state located at higher energy.

At this stage, several statements can be made regarding the 2PA properties of the various derivatives. Firstly, as is obvious from the various 2PA curves (Figure 9), metalation slightly promotes the 2PA cross-sections of the Zn(II) complexes relative to the corresponding free base phthalocyanines around the first maxima and at higher wavelengths. Then, regarding the various Zn(II) complexes, depending on the substitution sites or on the nature of the peripheral arms on the phthalocyanine, significant differences in the 2PA cross-sections at both maxima are stated between them (Table 2). Thus, an increase of *σ*_2_ is observed for the β-substituted derivative **ZnTOFPc1** with respect to **ZnPc** (200 GM at 820 nm), whereas its α-substituted analogue **ZnTOFPc3** (160 GM at 860 nm) exhibits the lowest cross-section at the maximum (even lower than for **ZnPc** used as reference). Notably, the **ZnTOFPc4** and **H_2_TOFPc4** derivatives, featuring extended and more conjugated peripheral substituents at the β-positions, exhibit the largest *σ*_2_ values of this series.

Finally, the two-photon brightness (*σ*_2_.*Φ*_F_), another important figure of merit for two-photon fluorescence imaging [55], was determined using these 2PA absorption cross-sections at their maxima. This time, in contrast to what was previously observed for the one-photon brightness, the Zn(II) complexes are slightly less brilliant than their free bases at the first 2PA maximum (880 nm), with the record value among these compounds being held by **ZnTOFPc4**.

### 2.9. DFT Studies

In order to get more insight into the nature and position of the peripheral fluorene-containing antennae with respect to the electronic properties and to also confirm the nature of the main electronic transitions observed in the absorption spectra of the new phthalocyanine derivatives presently synthesized, single-point energy- and time-dependent density functional theory (TD-DFT) calculations were carried out following prior geometry optimization in vacuo. Computationally simpler model compounds **ZnTOFPc2′**–**4′** and **H_2_TOFPc4′**, in which the *n*-butyl groups at the ninth position of fluorene in **ZnTOFPc2**–**4** and **H_2_TOFPc4** were replaced by methyl groups, were used for modeling the real compounds.

Optimization revealed that substitution of the peripheral arms at the β-positions, as for **ZnTOFPc2′**, allowed for the fluorene fragments to arrange around the flat aromatic core with only a slight deviation from the phthalocyanine plane after optimization (Figure S27, top left, Supplementary Materials). In contrast, substitution at the α-positions, as for **ZnTOFPc3′**, imparted more steric strain around the phthalocyanine core and induced stronger deviations more from the phthalocyanine plane after optimization (Figure S27, top right, Supplementary Materials). For **ZnTOFPc4′**, the replacement of the methoxy spacer by a 4-phenoxy spacer generates additional steric strain due to an interaction between the *ortho*-hydrogen atoms of this spacer and the α-hydrogen atoms of the phthalocyanine core. As a result, the 4-fluorenylethynylphenoxy fragments, albeit remaining perfectly co-planar (to maximize the *π* interaction along the fragment), cannot adopt a coplanar conformation with the phthalocyanine core (Figure S27, bottom left, Supplementary Materials). Notably, the replacement of zinc(II) by two protons did not appear to significantly affect the geometry of the phthalocyanine core after optimization (Figure S27, bottom left and right, Supplementary Materials).

**Figure 10:**
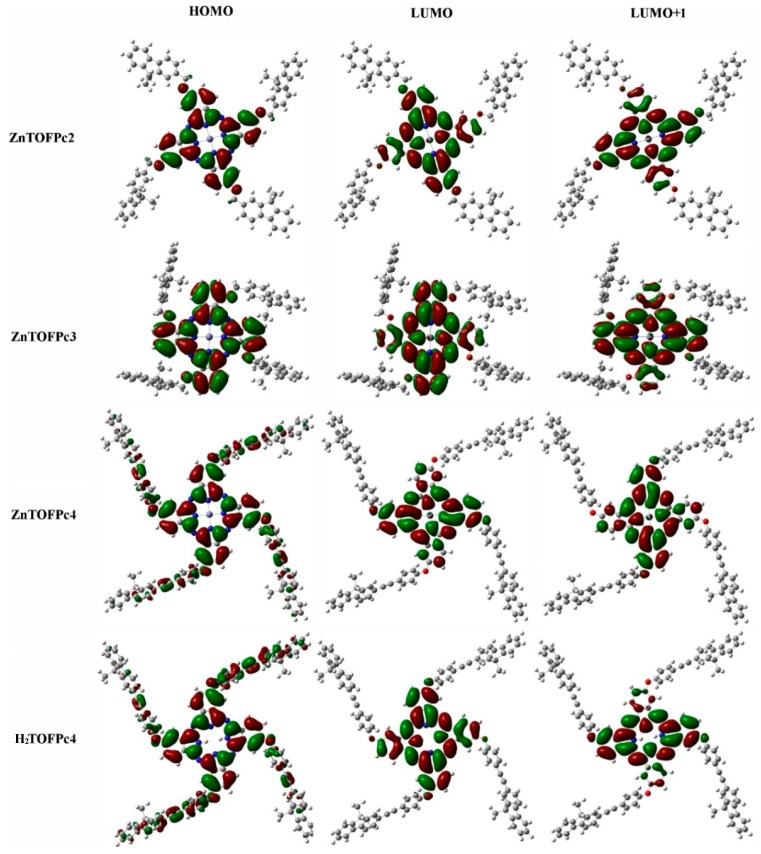
Frontier molecular orbitals of the four model compounds (left: highest occupied molecular orbital (HOMO), middle: lowest unoccupied molecular orbital (LUMO), right: LUMO + 1), shown with an isovalue of 0.01 [e bohr^−3^]^1/2.^

The molecular orbital (MO) analysis revealed that the frontier MOs are largely centered on the phthalocyanine ring in **ZnTOFPc2′** and **ZnTOFPc3′** (Figure 10). In contrast, for **ZnTOFPc4′** and **H_2_TOFPc2′**, the HOMOs extends significantly onto the peripheral arms. Thus, while the methoxy (–CH_2_O–) linker completely disrupted any electronic conjugation between the central phthalocyanine and the fluorene groups, its replacement by an ethynylphenoxy linker allowed maintaining some electronic interaction, possibly via n–π cross-conjugation.

**Figure 11:**
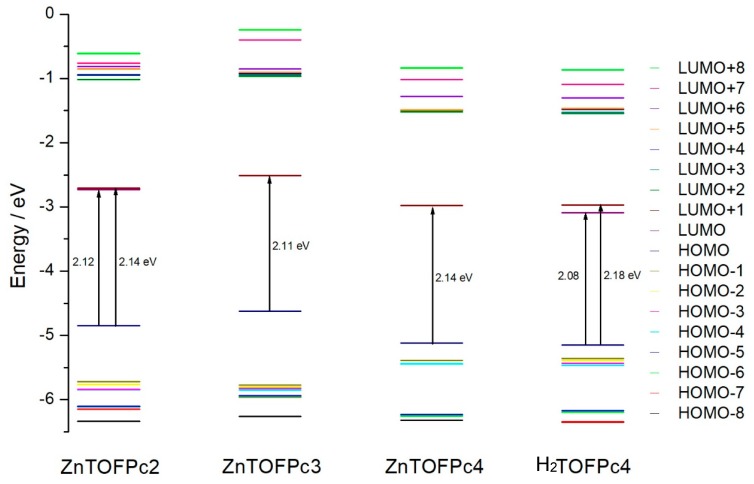
Frontier molecular energy levels (eV) of **ZnTOFPc2**, **ZnTOFPc3**, **ZnTOFPc4**, and **H_2_TOFPc4** obtained by time-dependent density functional theory (TD-DFT) B3LYP/6–31* (H, C, N, O)/ LANL2DZ (zinc). Only the energy gaps between the HOMO and the LUMO and LUMO + 1 levels are indicated.

For all the phthalocyanine derivatives, the HOMO–LUMO and HOMO–LUMO + 1 gaps are very close in energy and lay around 2.11–2.14 eV (Figure 11). Substitution at the α-position in **ZnTOFPc3** induces a slight decrease in the HOMO–LUMO and HOMO–LUMO + 1 gaps compared to substitution at the β-position in **ZnTOFPc2**. The stronger α-substituent effect on the HOMO–LUMO gaps can be related to the larger atomic coefficient on the carbon atom on this position in the HOMO, resulting in a relatively larger destabilization of the latter (of ca. 0.2 eV) by the electron-releasing peripheral antennae for **ZnTOFPc3** compared to **ZnTOFPc2** [43,54]. In comparison, the HOMO in **ZnTOFPc4**, featuring ethynylphenoxy-bridged antennae poorly overlapping with the *π*-manifold of the central phthalocyanine core at the β-positions, is less destabilized than in **ZnTOFPc2** (by ca. 0.3 eV), for which a more coplanar conformation can be adopted. However, given that the conjugated arms in **ZnTOFPc4** do also appear to stabilize the LUMO and LUMO+1 by roughly the same amount, the HOMO–LUMO gap of **ZnTOFPc4** remains nearly unaffected compared to that of **ZnTOFPc2**. The same energetical ordering for frontier MOs prevails in the corresponding free base (**H_2_TOFPc4**) except for the splitting (0.12 eV) of the LUMO and LUMO+1 MOs, which were nearly degenerate in **ZnTOFPc4**. As anticipated [52], this splitting is due to the disymmetrization of the phthalocyanine core induced when the Zn(II) atom is replaced by two protons.

The UV–visible absorption spectra of these compounds were then simulated by TD-DFT calculations over their first 40 excited states (Supplementary Materials). The spectra of **ZnTOFPc2′**–**4′** display a strong absorption band centered around 620–630 nm corresponding mainly to HOMO–LUMO and HOMO–LUMO + 1 π–π* transitions with high oscillator strengths (Figure S28, Supplementary Materials). These transitions formally correspond to the Q band [59]. Then, a second set of intense absorptions dominated by transition from deeper-lying *π*-MOs (HOMO-5 or HOMO-8) to the LUMO and LUMO + 1 was found around 350–390 nm (Table S2, Supplementary Materials). The latter set, not always well resolved on the experimental spectra from the transitions at higher energy (N band), formally corresponds to the Soret band (or B band) [59]. Notably, no fluorene-based π*←*π* excitations could be found among the allowed singlet states that were computed for **ZnTOFPc4′** (Table S2, Supplementary Materials). Such transitions, experimentally observed as a separate band around 270 nm for **ZnTOFPc2** and **ZnTOFPc3**, were also not among the 40 first singlet transitions computed for **ZnTOFPc2′**–**3′**. For **ZnTOFPc4′**, despite the fact that they might have been shifted to lower energies by the *π*-manifold extension on the peripheral antennae; we were not able to identify them among the computed states. However, some additional π–π* contributions featuring a charge transfer character between the arms and phthalocyanine core were computationally found for **ZnTOFPc4′** (Table S2, Supplementary Materials). These correspond to transitions that were not experimentally detected as separate bands for **ZnTOFPc4** (Figure 7a). The least energetic among them were certainly hidden within the vibronic bands on the low-energy side of the Q band, whereas the more energetic ones could be at the origin of some of the numerous shoulders observed on the high-energy side of the Soret band. Interestingly, the latter excitations correspond to intense (π*)_Flu_←(*π*)_Pc_ transitions, slightly higher in energy than the first Soret transitions (around 350–360 nm). In line with our expectations, they possibly result from intimate admixture of Pc-based and fluorene-based π*←*π* transitions close in energy. Finally, the spectrum simulated for the free base **H_2_TOFPc4′** was very similar to that of **ZnTOFPc4′**, except for the more pronounced splitting between the two intense transitions contributing to the Q band. Overall, considering that the experimental measurements were obtained with mixtures of stereoisomers and that the dielectric constant of the solvent was not taken in consideration during these calculations, the agreement between the simulated UV–V is spectra and the experimental ones can be considered as correct, especially regarding the shape and position of the main transitions observed (Soret and Q band).

## 3. Discussion

All the targeted free base and Zn(II) tetra-functional phthalocyanines (Figure 3) could be isolated as mixtures of regioisomers. Among them, those with the *n*-butyl chains on the peripheral fluorene groups (**ZnTOFPc2**–**4**, **H_2_TOFPc2,** and **H_2_TOFPc4**) exhibited sufficient solubilities in THF for their NMR characterization and appeared to be essentially non-aggregated in solution, at least below concentrations of 10 mM. The similarity between the absorption and emission spectra of **ZnTOFPc1** and **ZnTOFPc2** confirm that the introduction of *n*-butyl chains has almost no effect on their electronic structure, apart from perhaps slightly decreasing their fluorescence quantum yield. The position and the nature of their peripheral antennae, as well as the effect of metalation by zinc(II) on their optical properties of interest for PDT or fluorescence imaging, were then investigated.

### 3.1. Structural Effects on the Linear and Nonlinear Optical Properties

Given that the fluorescence of these phthalocyanines originates from their lowest singlet state (Q(0,0) band) after minimal structural reorganization and given that the transition to this singlet state results in a strong absorption a low energy, the linear optical properties of interest are directly linked to the HOMO–LUMO gap of the various phthalocyanines. In these compounds, the **Pc** core is more electron-rich than the peripheral antennae, as revealed by a stronger localization of the HOMO on this unit (Figure 7). Nevertheless, these antennae still behave as electron-releasing substituents toward the phthalocyanine core (e.g., comparing the Hammett σ_p_ coefficients [58,60] of alkoxy groups) [61]. In this respect, the electronic influence of the substitution site (α- or β-) on the HOMO–LUMO gap are mainly translated into a destabilizing influence on the HOMO. DFT reveals that this effect is stronger for α- than for β-positions (see above), resulting in the red shift observed for the first absorption (Q) and emission bands of **ZnTOFPc3** relative to those of **ZnTOFPc2**. In agreement with the work of Kobayashi and coworkers [43], we also find that the fluorescence quantum yield is lower for the α-substituted derivative (33% vs. 23%). Then, among the β-substituted derivatives **ZnTOFPc2** and **ZnTOFPc4**, replacement of the methoxy (–CH_2_O–) by a 4-ethynylphenoxy (–C≡C–C_6_H_4_–O–) linker does slightly decrease the electron-releasing power of the peripheral alcoholate ligand [62]. As a result, **ZnTOFPc4** presents a slightly blue-shifted and more intense Q-band absorption relative to **ZnTOFPc2** (Figure 7), but also a blue-shifted emission (Figure 8) with a slightly higher fluorescence quantum yield (37%). Thus, the one-photon brightness of the Zn(II) phthalocyanines at 680 nm (Q band) follows the order:

**ZnTOFPc4** > **ZnTOFPc2** > **ZnTOFPc3** > **ZnPc** (1)

Without surprise, the corresponding free bases are always more luminescent than the Zn(II) analogues, because the heavy-metal effect of zinc is suppressed [28,54]; however, at 680 nm, these species are not more one-photon brilliant than the Zn(II) phthalocyanines. Given that all these species undergo a very efficient energy transfer from their fluorene-based π–π* state to the Soret and Q state, excitation for fluorescence imaging might also be performed at higher energy, using the antenna-based absorption or the Soret band. In this respect, the conjugation between the phenoxy and the 2-fluorenyl group in **ZnTOFPc4** shifts the fluorene-based π–π* transition to lower energy, making both bands overlap and increases the one-photon brightness of this compound in this spectral range (Figure 8).

The lowest triplet state of these species is at the origin of the oxygen photosensitization process [28]. Since population of the latter is favored for Zn(II) derivatives (e.g., 65% for **ZnPc** vs. 17% for **H_2_Pc**), [27] and since intersystem crossing takes place partly at the expense of fluorescence [44], their efficiency for sensitizing oxygen follows roughly the reverse order to that shown above. Albeit slightly lower than for **ZnPc**, the *Φ*_Δ_ values of the metalated species **ZnTOFPc2**–**4** remainnevertheless sufficient for performing PDT, but free bases are unsuited for that task [2,6]. 

The importance of shifting the excitation wavelength required for PDT or other bio-related photonic applications (such as fluorescence imaging) in the optical window 650–900 nm was underlined by many researchers [54,55]. In this respect, two-photon excitation constitutes an appealing alternative over one-photon excitation [16,58], even more since the control of the solvent volume in which excitation takes place is much more spatially resolved [63]. With **ZnTOFPc2**–**4**, **H_2_TOFPc2**, and **H_2_TOFPc4**, we found that these photosensitizers might conveniently be two-photon excited between 840 and 900 nm, exhibiting two 2PA maxima in this spectral range. These correspond to the population of one-photon forbidden states having energies in between the Soret and Q bands [58]. The corresponding 2PA transitions were shown to be sensitive to metalation or peripheral modifications of the central phthalocyanine unit, a statement also previously made by Rebane and coworkers [58]. Thus, the promoting effect of metalation by Zn(II) on the cross-sections of the 2PA state around 880 nm presently stated was also apparent in their work. Additionally, we have now established that, for tetrasubstituted phtalocyanines, β-substitution is better than α-substitution to favor 2PA, a finding which somewhat contradicts their prediction based on the three-state model that they proposed to rationalize the 2PA properties of these compounds. Furthermore, among the β-substituted derivatives, it seems that those featuring less electron-releasing arms with extended *π*-manifolds able to cross-conjugate with the phthalocyanine core can give rise to even more active two-photon absorbers in the spectral region of interest [16]. While the electronic substituent effect is in line with Rebane’s predictions [58], the potential importance of cross-conjugation is unprecedented. In this respect, we would like to stress that substituents at β-positions require the use of σ_p_ Hammett coefficients, whereas substituent effects at α-positions are better modeled by σ_m_ Hammett [58,60], suggesting that mesomeric interactions are better transmitted through β-positions. As a result, the qualitative ordering previously found for *Φ*_F_ among the various Zn(II) phthalocyanines was retrieved for the 2PA cross-sections and, thus eq. 1 (see above) is also applicable for ranking their two-photon brightness at 880 nm.

### 3.2. Comparison with Porphyrin Analogues

For fluorescence imaging purposes, based on their one-photon brightness around 680 nm in THF (Q band), all Zn(II) phthalocyanines **ZnTOFPc2**–**4** appear significantly better than free base porphyrin systems such as **H_2_TOFP** or **H_2_OOFP** (Figure 1 and Table 1), even when these compounds are excited in their Soret band (i.e., the first intense absorption band of their spectra). For oxygen sensitization purposes, these phthalocyanines are slightly less efficient than their porphyrin analogues [25]. However, when compared to **H_2_OOFP** (1 − *Ф*_F_ − *Φ*_Δ_ = 0.23), it can be stated that the Zn(II) phthalocyanines **ZnTOFPc2** and **ZnTOFPc4** undergo roughly two times less unproductive decay (1 − *Ф*_F_ − *Φ*_Δ_ = 0.10 and 1 − *Ф*_F_ − *Φ*_Δ_ = 0.09, respectively), i.e., desexcitation via mechanisms other than fluorescence and oxygen sensitization (such as internal conversion or non-radiative decay from triplet state) [27,56]. Finally, all these new phthalocyanines largely outperform the octa-substituted and hexadeca-substituted free base porphyrin dendrimers (**H_2_OOFP** and **H_2_SOFP**) in terms of 2PA cross-sections (Table 2). They present a red-shifted and larger 2PA cross-section maximum, resulting in a much larger two-photon brightness at this maximum, both features being desirable for two-photon PDT or two-photon fluorescence imaging [55]. Thus, in the perspective of developing new one- or two-photon fluorescent photosensitizers for theranostic applications, these results are very encouraging to further explore the potential of such phthalocyanine derivatives.

## 4. Materials and Methods

### 4.1. General

Unless otherwise stated, all solvents used in reactions were distilled using common purification protocols [64], except DMF and ^i^Pr_2_NH which were dried on molecular sieves (3 Å). Compounds were purified by chromatography on silica gel using different mixtures of eluents as specified. ^1^H- and ^13^C-NMR spectra were recorded on a BRUKER Ascend 400 and 500 at 298 K. Fourrier-transform Infrared (FTIR) spectra were recorded on a BRUKER IFS 28 spectrometer. The chemical shifts are referenced to internal tetramethylsilane. High-resolution mass spectra (HRMS) were recorded on different spectrometers: a Bruker MicrOTOF-Q II, a Thermo Fisher Scientific Q-Exactive in electrospray ionisation (ESI) positive mode, and a Bruker Ultraflex III MALDI Spectrometer at CRMPO (Centre Regional de Mesures Physiques de l’Ouest) in Rennes. Reagents were purchased from commercial suppliers and used as received.

### 4.2. Synthesis of Precursors

Firstly, 4-nitrophthalonitrile (**1**) was accessed in few steps with good yields from phthalimide (commercial product) [37], while 3-nitrophthalic anhydride (commercial product) was used to obtain 3-nitrophthalonitrile (**2**) [38]. The compounds 9*H*-(fluoren-2-yl)methanol (**3**) [23], 2-bromo-9,9-dibutyl-9*H*-fluorene (**4**) [40], aldehyde 9,9-dibutyl-fluorene-2-carbaldehyde (**5**) [40], 4-(4-bromophenoxy)phthalonitrile, and 9,9-dibutyl-2-ethynyl-9*H*-fluorene (**9**) [40,65] were prepared as reported earlier. The compound 3-(4-bromophenoxy)phthalonitrile (**8**) was obtained as reported for (**7**) [1]. 

**(9,9-Dibutyl-9*H*-fluoren-2-yl)methanol (6):** In a Schlenk tube, 9,9-dibutyl-fluorene-2-carbaldehyde (**5**) (3.0 g, 9.8 mmol, 1 equivalent (eq.)) was dissolved in ethanol (100 mL) under argon. An ice-water bath was fixed for cooling the system to 0 °C. Then, NaBH_4_ (444 mg, 11.8 mmol, 1.2 eq.) was added. The system was firstly stirred at 0 °C for 1 h, followed by another 2 h at room temperature. The mixture was extracted three times with ethyl acetate/water and washed with saturated NaCl. After evaporation of the volatiles, the residue was further purified by silica chromatography using CH_2_Cl_2_/heptane (1:1) as eluent. The (9,9-dibutyl-9*H*-fluoren-2-yl)methanol (**6**) was isolated as white crystals (2.94 g, 97% yield). ^1^H-NMR (400 MHz, CDCl_3_) *δ* = 7.72–7.66 (m, 2H), 7.37–7.27 (m, 5H), 4.78 (s, 2H), 2.01–1.91 (m, 4H), 1.12–1.03 (m, 4H), 0.67 (t, *J* = 7.3 Hz, 6H), 0.64–0.55 (m, 4H). ^13^C-NMR (101 MHz, CDCl_3_) *δ* = 151.2, 150.8, 140.8, 139.8, 127.0, 126.7, 125.7, 122.9, 121.5, 119.7, 119.6, 65.8, 55.0, 40.2, 26.0, 23.1, 13.8.

**4-((9,9-Dibutyl-9*H*-fluoren-2-yl)ethynyl)phenol (11)**: In a Schlenk tube, to a mixture of 4-iodophenol (0.92 g, 4.17 mmol, 1 eq.), **9** (1.51 g, 5 mmol, 1.2 eq.), Pd(PPh_3_)_2_Cl_2_ (59 mg, 0.08 mmol, 2% eq.), and CuI (16 mg, 0.08 mmol, 2% eq.) were added with THF (20 mL) and NEt_3_ (20 mL) under argon. Then, the system was degassed by freeze–pump–thaw twice and heated for 24 h at 70 °C. After being evaporated, the residue was further purified by chromatography (CH_2_Cl_2_), giving the title compound as a yellow powder (1.14 g, 69% yield). ^1^H-NMR (400 MHz, CDCl_3_) *δ* = 7.70–7.68 (m, 1H), 7.67 (d, *J* = 8.0 Hz, 1H), 7.54–7.49 (m, 2H), 7.48–7.44 (m, 2H), 7.37–7.29 (m, 3H), 6.88–6.77 (m, 2H), 5.02 (s, 1H), 2.03–1.92 (m, 4H), 1.08 (h, *J* = 7.1 Hz, 4H), 0.68 (t, *J* = 7.3 Hz, 6H), 0.64–0.50 (m, 4H). ^13^C-NMR (101 MHz, CDCl_3_) *δ* = 155.7, 151.1, 150.9, 141.3, 140.6, 133.4, 130.6, 127.6, 127.0, 125.95, 123.0, 121.8, 120.1, 119.7, 116.0, 115.7, 89.3, 55.2, 40.4, 26.00, 23.21, 14.0.

**4-((9*H*-Fluoren-2-yl)methoxy)phthalonitrile (Pn1)**: To a mixture of 4-nitrophtalonitrile (**1**) (1.04 g, 6 mmol, 1 eq.) and 9*H*-(fluoren-2-yl)methanol (**3**) (1.77 g, 9 mmol, 1.5 eq.) in dry DMF (20 mL) was added portion-wise anhydrous K_2_CO_3_ within 2 h (2.49 g,18 mmol, 3 eq.). The mixture was stirred for further 72 h at 50 °C under argon. Then, the reaction mixture was poured into ice water (50 mL) and the precipitate formed was filtered off, washed with cold water and methanol, and purified by chromatography using CH_2_Cl_2_/heptane (2:1) then CH_2_Cl_2_ (100%) as eluents and recrystallized from MeOH/CH_2_Cl_2_ to give the title compound as a white powder. Yield: 5%. ^1^H-NMR (400 MHz, CDCl_3_) *δ* = 7.82 (d, *J* = 7.8 Hz, 1H), 7.80 (d, *J* = 7.6 Hz, 1H) 7.70 (d, *J* = 8.8 Hz, 1H), 7.57 (d, *J* = 8.4 Hz, 2H), 7.43–7.37 (m, 2H), 7.37–7.31 (m, 2H), 7.28 (dd, *J* = 8.8 Hz, 1H), 5.21 (s, 2H), 3.93 (s, 2H). ^13^C-NMR (101 MHz, CDCl_3_) *δ* = 161.8, 144.0, 143.38, 142.6, 140.9, 135.2, 132.8, 127.3, 126.9, 126.5, 125.1, 124.5, 120.2, 120.1, 120.0, 119.7, 117.5, 115.6, 115.3, 107.5, 71.5, 36.9. FT-IR *ῡ* (cm^−1^): 3067–3040 (w, aromatic C–H), 2929 (w, aliphatic C–H), 2224 (m, C≡N), 1709, 1596, 1487, 1403, 1323, 1291, 1251, 1174, 1093, 1035, 999, 826, 763, 750, 731. HRMS-ESI: [M + Na]^+^ (C_22_H_14_N_2_ONa): *m*/*z* = 345.0999 (calculated: 345.0998). Analytically calculated (%) for C_22_H_14_N_2_O: C, 81.97; H, 4.38; N, 8.69. Found: C, 82.00; H, 4.46; N, 8.41.

**4-((9,9-Dibutyl-9*H*-fluoren-2-yl)methoxy)phthalonitrile (Pn2)**: 9,9-Dibutyl-9*H*-fluoren-2-yl)-methanol (**6**; 925 mg, 3 mmol, 1.5 eq.) and 4-nitrophthalonitrile (**1**; 346 mg, 2 mmol, 1 eq.) were dissolved in dry DMF (10 mL) under argon, and anhydrous K_2_CO_3_ (829 mg, 6 mmol, 3 eq.) was added portion-wise within 2 h and stirred for 72 h at room temperature. At the end of the reaction, the reaction mixture was poured into ice water (25 mL). The crude product was filtered and washed with water and MeOH and dried. The product was purified by silica gel column using heptane/CH_2_Cl_2_ (2:1) as an eluent and recrystallized from MeOH/CH_2_Cl_2_ as a white powder. Yield: 62%. ^1^H-NMR (400 MHz, CDCl_3_) *δ* = 7.73 (d, *J* = 7.6 Hz, 1H), 7.72–7.68 (m, 2H), 7.36 (dd, *J* = 8.9 Hz, 2.9 Hz, 3H), 7.34–7.31 (m, 3H), 7.28 (dd, *J*_1_ = 8.8 Hz, *J*_2_ = 2.6 Hz, 1H), 5.25 (s, 2H), 1.95 (m, 4H), 1.05 (h, *J* = 7.3 Hz, 4H), 0.66 (t, *J* = 7.3 Hz, 6H), 0.61–0.50 (m, 4H). ^13^C-NMR (101 MHz, CDCl_3_) *δ* = 161.8, 151.5, 150.8, 142.0, 140.3, 135.2, 133.0, 127.6, 126.9, 126.4, 122.9, 122.1, 120.2, 120.1, 119.9, 119.8, 117.5, 115.6, 115.2, 107.5, 71.6, 55.1, 40.1, 25.9, 23.0, 13.8. FT-IR *ῡ* (cm^−1^): 3119–3043 (w, aromatic C–H), 2956–2856 (s, aliphatic C–H), 2234 (s, C≡N), 1595, 1493, 1466, 1450, 1320, 1257, 1212, 1085, 1026, 876, 841,821, 776, 741. HRMS-ESI (*m*/*z*): [M + Na]^+^ (C_30_H_30_N_2_ONa): *m*/*z* = 457.2255 (calculated 457.2255). Analytically calculated (%) for C_30_H_30_N_2_O: C, 82.91; H, 6.96; N, 6.45. Found: C, 82.64; H, 6.83; N, 6.28.

**3-((9,9-Dibutyl-9*H*-fluoren-2-yl)methoxy)phthalonitrile (Pn3)**: 9,9-Dibutyl-9*H*-fluoren-2-yl)-methanol (**6**; 925 mg, 3 mmol, 1.5 eq.) and 3-nitrophthalonitrile (**2**; 346 mg, 2 mmol, 1 eq.) were dissolved in dry DMF (10 mL) under argon, and anhydrous K_2_CO_3_ (829 mg, 6 mmol, 3 eq.) was added portion-wise within 2 h and stirred for 72 h at 50 °C. At the end of the reaction, the reaction mixture was poured into ice water (25 mL). The crude product was filtered, and then washed with water and MeOH before being dried. The desired title product was purified by silica gel column using heptane/CH_2_Cl_2_ (2:1) as an eluent and recrystallized from MeOH/CH_2_Cl_2_ and isolated as a white powder. Yield: 48%. ^1^H-NMR (400 MHz, CDCl_3_) *δ* = 7.74–7.67 (m, 2H), 7.58–7.53 (m, 1H), 7.39 (d, *J* = 7.7 Hz, 2H), 7.37–7.27 (m, 5H), 5.39 (s, 2H), 2.01–1.92 (m, 4H), 1.04 (h, *J* = 6.9 Hz, 4H), 0.64 (t, *J* = 7.3 Hz, 6H), 0.60–0.46 (m, 4H). ^13^C-NMR (101 MHz, CDCl_3_) *δ* = 161.0, 151.5, 150.8, 141.8, 140.3, 134.2, 133.2, 127.5, 126.9, 126.0, 125.3, 122.9, 121.7, 120.0, 119.9, 117.9, 117.1, 115.2, 113.0, 105.7, 72.0, 55.0, 40.1, 26.0, 23.0, 13.8. FT-IR *ῡ* (cm^−1^): 3079 (w, aromatic C–H), 2955–2858 (s, aliphatic C–H), 2232 (s, C≡N), 1576, 1470, 1427, 1378, 1286, 1258, 1135, 1057, 1027, 824, 883, 842, 777, 754, 740. HRMS-ESI (*m*/*z*): [M + Na]^+^ (C_30_H_30_N_2_ONa): *m*/*z* = 457.2245 (calculated 457.2250). Analytically calculated (%) for C_30_H_30_N_2_O: C, 82.91; H, 6.96; N, 6.45. Found: C, 82.64; H, 6.92; N, 6.22.

**4-(4-((9,9-Dibutyl-9*H*-fluoren-2-yl)ethynyl)phenoxy)phthalonitrile (Pn4):** 4-Nitrophthalo-nitrile (**1**; 260 mg, 1.5 mmol, 1 eq.) and compound **11** (888 mg, 2.25 mmol, 1.5 eq.) were dissolved in anhydrous DMF (10 mL). After stirring for 10 min, anhydrous K_2_CO_3_ (622 mg, 4.5 mmol, 3 eq.) was added portion-wise within 2 h. The reaction mixture was stirred at room temperature for 4 h under argon atmosphere. It was then poured into ice water, and the obtained precipitate was filtered, washed with water and MeOH, and dried in vacuum. Then, it was also purified by column chromatography with silica gel eluting with heptane/CH_2_Cl_2_ (2:1) and recrystallized from CH_2_Cl_2_/MeOH as a yellow product. Yield: 61%. ^1^H-NMR (400 MHz, CDCl_3_) *δ* = 7.75 (d, *J* = 8.7 Hz, 1H), 7.73–7.68 (m, 2H), 7.68–7.64 (m, 2H), 7.55–7.50 (m, 2H), 7.38–7.31 (m, 4H), 7.30–7.26 (m, 1H), 7.10–7.05 (m, 2H), 1.99 (t, *J* = 8.3 Hz, 4H), 1.09 (h, *J* = 7.4 Hz, 4H), 0.68 (t, *J* = 7.3 Hz, 6H), 0.65–0.50 (m, 4H). ^13^C-NMR (101 MHz, CDCl_3_) *δ* = 161.4, 153.5, 151.1, 151.0, 141.9, 140.4, 135.6, 134.0, 130.8, 127.8, 127.1, 126.1, 123.1, 122.0, 121.8, 121.8, 121.0, 120.7, 120.2, 119.8, 118.0, 115.4, 115.0, 109.5, 91.6, 88.0, 55.2, 40.3, 26.0, 23.2, 13.9. FT-IR *ῡ* (cm^−1^): 3060–3040 (w, aromatic C–H), 2956–2859 (s, aliphatic C–H), 2233 (s, C≡N), 2206 (w, C≡C), 1592, 1559, 1503, 1484, 1466, 1285, 1249, 1212, 1170, 1155, 953, 895, 837, 816, 773, 736. HRMS-ESI (*m*/*z*): [M + Na]^+^ (C_37_H_32_N_2_ONa): *m*/*z* = 543.2411 (calculated: 543.2406). Analytically calculated (%) for C_37_H_32_N_2_O: C, 85.35; H, 6.19; N, 5.38. Found: C, 85.10; H, 6.19; N, 5.29.

### 4.3. Phthalocyanine Synthesis

**ZnTOFPc1 (mixture of regioisomers):** A mixture of **Pn1** (200 mg, 0.62 mmol, 1 eq) and anhydrous Zn(OAc)_2_ (57 mg, 0.31 mmol, 0.5 eq.) in dry dimethylaminoethanol (DMAE) (2 mL) was heated to 160 °C and stirred for 24 h under argon. The blue solution was allowed to cool to room temperature and poured into cold methanol, left for half an hour, and then crude product was filtered off. Then, this solid was washed with water and hot methanol, and dried in vacuum. The blue product was filtered over silica using THF/heptane (10:1) as eluent and finally recrystallized from MeOH/CH_2_Cl_2_. The title product was obtained as a blue solid (17 mg, 8% yield). ^1^H-NMR (400 MHz, THF-*d*_8_) *δ* = 9.12–8.82 (m, 4H), 8.82–8.49 (m, 4H), 8.01–7.87 (m, 8H), 7.84 (t, *J* = 7.1 Hz, 4H), 7.80–7.57 (m, 8H), 7.52 (d, *J* = 7.2 Hz, 4H), 7.38–7.24 (m, 8H), 5.64 (s, 8H), 3.94 (s, 8H). ^13^C-NMR (101 MHz, THF-*d*_8_) *δ* = 161.0, 160.9, 160.8, 153.1, 152.6, 144.0, 144.0, 143.9, 143.9, 141.9, 141.8, 136.6, 136.5, 132.0, 131.9, 131.8, 126.9, 126.8, 125.2, 124.8, 120.1, 120.0, 71.0, 36.9. FT-IR *ῡ* (cm^−1^): 3056 (w, aromatic C–H), 2919–2853 (m, aliphatic C–H), 1606, 1487, 1456, 1376, 1338, 1277, 1224, 1118, 1089, 1050, 948, 822, 762, 744. HRMS-ESI (*m*/*z*): [M + H]^+^ (C_88_H_57_N_8_O_4_Zn): *m*/*z* = 1353.3787 (calculated: 1353.3787).

**ZnTOFPc2 (mixture of regioisomers):** A mixture of **Pn2** (200 mg, 0.46 mmol, 1 eq.) and anhydrous Zn(OAc)_2_ (42 mg, 0.23 mmol, 0.5 eq.) in dry DMAE (2 mL) was heated at 160 °C for 24 h under argon. The resulting intense colored solution was cooled to room temperature, and the suspension was poured into cold methanol. The precipitated solid was then filtered off and was washed with hot methanol. The crude product was purified by column chromatography on silica gel using CH_2_Cl_2_/heptane (100:1) as eluent and then recrystallized from MeOH/CH_2_Cl_2_. The title product was isolated as a blue solid (54 mg, 26% yield). ^1^H-NMR (400 MHz, THF-*d*_8_) *δ* = 9.19–9.04 (m, 4H), 8.94–8.75 (m, 4H), 7.91–7.87 (m, 4H), 7.87–7.66 (m, 16H), 7.47–7.24 (m, 12H), 5.72 (s, 8H), 2.25–2.01 (m, 16H), 1.17–1.02 (m, 16H), 0.83–0.49 (m, 40H). ^13^C-NMR (101 MHz, THF-*d*_8_) *δ* = 161.2, 152.7, 152.4, 151.2, 151.0, 141.5, 141.5, 141.4, 136.9, 132.3, 132.1, 132.0, 127.3, 127.1, 127.0, 127.0, 123.6, 123.0, 122.8, 122.6, 120.0, 106.3, 71.2, 55.3, 40.6, 26.4, 23.4, 13.6. FT-IR ν (cm^−1^): 3056 (w, aromatic C–H), 2956–2858 (s, aliphatic C–H), 1607, 1586, 1488, 1454, 1374, 1337, 1275, 1217, 1118, 1091, 1050, 932, 823, 772, 739. HRMS-ESI (*m*/*z*): [M]^+^ (C_120_H_120_N_8_O_4_Zn): *m*/*z* = 1800.8716 (calculated 1800.8718). Analytically calculated (%) for C_120_H_120_N_8_O_4_Zn: C, 79.91.35; H, 6.71; N, 6.21. Found: C, 79.49; H, 6.65; N, 5.82.

**TOFPc2 (mixture of regioisomers):** A mixture of **Pn2** (200 mg, 0.46 mmol) and 1,8-diazabycyclo[5.4.0]undec-7-ene (DBU) (five drops) in degassed 1-pentanol (2 mL) was heated to 160 °C and stirred for 24 h under argon. The blue solution was allowed to cool to room temperature and poured into cold methanol, left for half an hour, and then crude product was filtered off. Then, this solid was washed with water and hot methanol, and dried in vacuum. The blue product was filtered over silica using heptane/CH_2_Cl_2_ as eluent and then recrystallized from MeOH/CH_2_Cl_2_. The title product was obtained as a blue solid (48 mg, 24% yield). ^1^H-NMR (400 MHz, THF-*d*_8_) *δ* = 8.58–8.38 (m, 2H), 8.37–8.21 (m, 2H), 8.13–7.90 (m, 4H), 7.90–7.69 (m, 16H), 7.56–7.21 (m, 16H), 5.51 (s, 8H), 2.29–2.04 (m, 16H), 1.12 (h, *J* = 8.0 Hz, 16H), 0.88–0.55 (m, 40H), −4.09 (s, 2H). ^13^C-NMR (101 MHz, THF-*d*_8_) *δ* = 161.1, 161.0, 160.8, 160.7, 151.2, 151.1, 151.0, 141.5, 141.4, 136.7, 127.3, 127.2, 127.1, 123.1, 122.6, 120.2, 120.1, 71.1, 55.3, 40.6, 26.5, 23.4, 13.7. FT-IR ν (cm^−1^): 3290 (w, N–H), 3056 (w, aromatic C–H), 2956–2858 (s, aliphatic C–H), 1611, 1455, 1425, 1376, 1344, 1323, 1213, 1114, 1096, 1051, 1004, 926, 823, 739. HRMS-ESI (*m*/*z*): [M]^+^ (C_120_H_122_N_8_O_4_): *m*/*z* = 1738.9584 (calculated: 1738.9583). Analytically calculated (%) for C_120_H_122_N_8_O_4_.2MeOH: C, 81.21; H, 7.26; N, 6.21. Found: C, 80.92; H, 7.22; N, 6.10.

**ZnTOFPc3 (mixture of regioisomers):** A mixture of **Pn3** (200 mg, 0.46 mmol, 1 eq.), anhydrous Zn(OAc)_2_ (42 mg, 0.23 mmol, 0.5 eq.), and DBU (5 drops) in degassed *n*-pentanol (2 mL) was heated at 160 °C for 24 h under argon. The resulting intense colored solution was cooled to room temperature, and the suspension was poured into cold methanol. The precipitated solid was then filtered off and washed with hot methanol. The crude product was purified by column chromatography on silica gel using CH_2_Cl_2_/EtOH (100:1) as eluent and then recrystallized from MeOH/CH_2_Cl_2_. The title product was obtained as a blue solid (44 mg, 21% yield). ^1^H-NMR (400 MHz, THF-*d*_8_) *δ* = 9.18–8.99 (m, 4H), 8.90–8.70 (m, 4H), 7.94–7.87 (m, 4H), 7.87–7.64 (m, 16H), 7.46–7.39 (m, 4H), 7.38–7.25 (m, 8H), 5.72 (s, 8H), 2.25–2.00 (m, 16H), 1.18–1.03 (m, 16H), 0.82–0.53 (m, 40H). ^13^C-NMR (101 MHz, THF-*d*_8_) *δ* = 162.7, 162.7, 162.6, 162.5, 154.8, 154.6, 154.1, 153.8, 153.2, 152.7, 152.5, 143.0, 142.9, 142.5, 142.1, 138.4, 133.8, 133.5, 128.6,128.5, 128.2, 125.0, 124.5, 124.2, 124.1, 121.5, 110.2, 107.8, 72.7, 56.8, 42.1, 27.9, 24.9, 15.1. FT-IR ν (cm^−1^): 3048 (w, aromatic C–H), 2952–2857 (s, aliphatic C–H), 1584, 1487, 1467, 1454, 1334, 1267, 12226, 1119, 1083, 1064, 878, 824, 800, 738. HRMS-ESI (*m*/*z*): [M + H]^+^ (C_120_H_121_N_8_O_4_Zn): *m*/*z* = 1801.8801 (calculated 1801.8796). Analytically calculated (%) for C_120_H_120_N_8_O_4_Zn: C, 79.91; H, 6.71; N, 6.21. Found: C, 79.36; H, 6.62; N, 5.82.

**ZnTOFPc4 (mixture of regioisomers)**: A mixture of **Pn4** (200 mg, 0.38 mmol, 1 eq.) and anhydrous Zn(OAc)_2_ (35 mg, 0.19 mmol, 0.5 eq.) in dry DMAE (2 mL) was heated at 140 °C for 16 h under argon. The resulting intense colored solution was cooled to room temperature, and the suspension was poured into cold methanol. The precipitated solid was then filtered off, and the precipitate was washed with hot methanol. The crude product was purified by column chromatography on silica gel using CH_2_Cl_2_/heptane (4:1) as eluent and then recrystallized from MeOH/CH_2_Cl_2_. The title compound was obtained as a blue solid (82 mg, 40% yield) ^1^H-NMR (400 MHz, THF-*d*_8_) *δ* = 9.17–8.98 (m, 4H), 8.84–8.68 (m, 4H), 7.89–7.67 (m, 20H), 7.64 (dd, *J_1_* = 8.8, *J_2_* = 3.5 Hz, 4H), 7.59–7.44 (m, 12H), 7.42–7.36 (m, 4H), 7.35–7.26 (m, 8H), 2.14–1.97 (m, 16H), 1.19–1.02 (m, 16H), 0.73–0.54 (m, 40H). ^13^C-NMR (101 MHz, THF-*d*_8_) *δ* = 158.5, 158.4, 158.3, 152.6, 152.4, 152.2, 152.1, 151.9, 151.1, 151.0, 141.9, 141.8, 140.9, 140.4, 134.3, 134.2, 133.7, 130.9, 127.7, 127.2, 126.1, 126.0, 124.4, 124.2, 123.0, 122.3, 121.1, 120.3, 119.9, 119.4, 119.2, 112.7, 112.4, 90.3, 89.34, 89.2, 55.3, 40.4, 26.3, 23.3, 13.6. FT-IR ν (cm^−1^): 3063 (w, aromatic C–H), 2956–2858 (s, aliphatic C–H), 2179 (vw, C≡C), 1597, 1504, 1457, 1394, 1336, 1259, 1230, 1161, 1120, 1090, 1045, 945, 889, 828 774, 737. HRMS-ESI (*m*/*z*): [M]^+^ (C_148_H_128_N_8_O_4_Zn): *m*/*z* = 2144.958 (calculated: 2144.934). Analytically calculated (%) for C_148_H_128_N_8_O_4_Zn: C, 82.75; H, 6.01; N, 5.22. Found: C, 82.24; H, 5.92; N, 5.11.

**TOFPc4 (mixture of regioisomers):** A mixture of **Pn4** (200 mg, 0.38 mmol) and DBU (five drops) in degassed *n*-pentanol (2 mL) was heated to 140 °C and stirred for 16 h under argon. The blue solution was allowed to cool to room temperature and poured into cold methanol, left for half an hour. The crude product was filtered off. Then, this solid was washed with water and hot methanol, and dried in vacuum. The blue product was filtered over silica using heptane/CH_2_Cl_2_ (3:2) as eluent and then recrystallized from CH_2_Cl_2_/MeOH. The title compound was obtained as a blue solid (74 mg, 37% yield). ^1^H-NMR (400 MHz, THF-*d*_8_) *δ* = 8.50–7.88 (broad, 8H), 7.88–7.78 (m, 8H), 7.77–7.67 (m, 12H), 7.66–7.42 (m, 16H), 7.42–7.36 (m, 4H), 7.34–7.24 (m, 8H), 2.18–1.97 (m, 16H), 1.18–1.02 (m, 16H), 0.77–0.54 (m, 40H), −5.65 (s, 2H). ^13^C-NMR (101 MHz, THF-*d*_8_) *δ* = 158.6, 157.9, 157.8, 151.1, 151.0, 141.9, 141.8, 140.9, 133.8, 131.0, 127.7, 127.1, 126.1, 123.0, 122.3, 120.0, 119.6, 90.6, 89.4, 55.3, 40.4, 26.3, 23.3, 13.6. FT-IR ν (cm^−1^): 3290 (vw, N–H), 3056 (w, aromatic C–H), 2952–2857 (s, aliphatic C–H), 2186 (vw, C≡C), 1596, 1504, 1465, 1419,1393, 1334, 1320, 1228, 1159, 1112, 1092, 1006, 928, 827, 736. HRMS-ESI (*m*/*z*): [M]^+^ (C_148_H_130_N_8_O_4_): *m*/*z* = 2082.961. Analytically calculated (%) for C_148_H_130_N_8_O_4_: C, 85.27; H, 6.29; N, 5.37. Found: C, 85.25; H, 6.24; N, 5.38.

### 4.4. Spectroscopic Measurements

All photophysical measurements were performed with freshly prepared air-equilibrated THF solutions (HPLC grade) at room temperature (298 K). UV–Vis absorption spectra were recorded on a Jasco V-570 spectrophotometer. Steady-state fluorescence measurements were performed on dilute solutions (ca. 10^−6^ M, optical density <0.1) contained in standard 1-cm quartz cuvettes using an Edinburgh Instrument (FLS920) spectrometer in photon-counting mode. Fully corrected emission spectra were obtained, for each compound, after excitation at the wavelength of the absorption maximum, with *A_λ_*_ex_ *<* 0.1 to minimize internal absorption. Fluorescence quantum yields were measured according to literature procedures [66,67]. 

### 4.5. Two-Photon Absorption Experiments

To span the 790–980-nm range, an Nd:YLF-pumped Ti/sapphire oscillator (Chameleon Ultra, Coherent) was used generating 140-fs pulses at a 80-MHz rate. The excitation power was controlled using neutral density filters of varying optical density mounted in a computer-controlled filter wheel. After a five-fold expansion through two achromatic doublets, the laser beam was focused by a microscope objective (10×, NA (Numerical Aperture) 0.25, Olympus, Shinjuku, Tokyo, Japan) into a standard 1-cm absorption cuvette containing the sample. The applied average laser power arriving at the sample was typically between 0.5 and 40 mW, leading to a time-averaged light flux in the focal volume on the order of 0.1–10 mW/mm^2^. The fluorescence from the sample was collected in epifluorescence mode, through the microscope objective, and reflected by a dichroic mirror (Chroma Technology Corporation, Bellows Falls, VT, USA; “red” filter set: 780dxcrr). This made it possible to avoid the inner filter effects related to the high dye concentrations used (10^−4^ M) by focusing the laser near the cuvette window. Residual excitation light was removed using a barrier filter (Chroma Technology; “red”: e750sp–2p). The fluorescence was coupled into a 600-µm multimode fiber by an achromatic doublet. The fiber was connected to a compact CCD (Charge-Coupled Device)-based spectrometer (BTC112-E, B&WTek, Newark, DE, USA), which measured the two-photon excited emission spectrum. The emission spectra were corrected for the wavelength dependence of the detection efficiency using correction factors established through the measurement of reference compounds having known fluorescence emission spectra. Briefly, the set-up allowed for the recording of corrected fluorescence emission spectra under multiphoton excitation at variable excitation power and wavelength. The 2PA cross-sections (*σ*_2_) were determined from the two-photon excited fluorescence (TPEF) cross-sections (*σ*_2_.*Φ*_F_) and the fluorescence emission quantum yield (*Φ*_F_). TPEF cross-sections of 10^−4^ M dichloromethane solutions were measured relative to fluorescein in 0.01 M aqueous NaOH using the well-established method described by Xu and Webb [57] and the appropriate solvent-related refractive index corrections [68]. The quadratic dependence of the fluorescence intensity on the excitation power was checked for each sample and all wavelengths.

### 4.6. Measurement of Singlet Oxygen Quantum Yields (Φ_Δ_)

Measurements were performed on a Fluorolog-3 (Horiba Jobin Yvon, Instruments Inc., Edison, NJ, USA), using a 450-W Xenon lamp, with air-equilibrated solutions. The optical density of the reference and the sample solution were set equal to 0.15 at the excitation wavelength (maximum of the Soret band). The emission at 1272 nm was detected using a liquid nitrogen-cooled Ge-detector model (EO-817L, North Coast Scientific Co, Santa Rosa, CA, USA). The emission spectra were corrected for the wavelength dependence of the lamp intensity and the excitation monochromator efficiency (excitation correction). Singlet oxygen quantum yields *Φ*_Δ_ were determined in toluene/pyridine (99:1) *v*/*v* solutions, using zinc(II) phtalocyanine (**ZnPc**) in a toluene/pyridine (99:1) mixture as reference solution (*Φ*_Δ_[**ZnPc**] = 0.61) [53,54] and were estimated from corrected ^1^O_2_ luminescence at 1272 nm. The uncertainty of the values of the singlet oxygen quantum yields determined by this method was estimated to be ±0.05.

### 4.7. DFT Computations

Density functional theory [69,70] calculations were performed with the hybrid Becke-3 parameter exchange functional [71,72,73] and the Lee–Yang–Parr nonlocal correlation functional (B3LYP) [74] implemented in the Gaussian 09 (Revision B.01) program suite [75] using the pseudo-potentials LANL2DZ for zinc and the 6-31G* basis set for C, H, N, O with the default convergence criteria implemented in the program. The pseudo-potentials LANL2DZ were used for heavy atoms such as Zn atom. Calculations were carried out on the OCCIGEN calculator of the Centre Informatique National de l’Enseignement Supérieur (CINES, Montpellier, France) under project 2018-A0040805032. Figures were generated with GaussView 5.0, and data were analyzed with GaussSum 2.2.6.1.

## 5. Conclusions

The synthesis and characterization of a series of metalated (**ZnTOFPc1**–**3**) and free base (**H_2_TOFPc2**) phthalocyanine-cored derivatives peripherally functionalized with 2-fluorenoxy antennae, as well as two analogues (**ZnTOFPc4** and **H_2_TOFPc4**) featuring expanded fluorene-containing antennae cross-conjugated to the central core, were reported here. These new tetrasubstituted phthalocyanines were isolated as mixtures of regioisomers from the corresponding α- or β-monosubstituted phthalonitrile precursors (**Pn1**–**4**). In terms of photophysical properties, these phthalocyanines exhibit significantly higher luminescence quantum yields (29%–47%) than their known porphyrin analogues. For fluorescence imaging purposes, their improved one- and two-photon brightness in the near-IR region is supplemented by a very efficient energy transfer from the peripheral fluorenyl units toward the central macrocycle, opening the possibility to perform excitation at higher energies. For PDT purposes, the singlet oxygen generation capability of the Zn(II) derivatives is comparable to that of larger free base porphyrin analogues, whereas their two-photon absorption cross-sections (*σ*_2_) are significantly improved. Thus, our data point to the advantages of β-functionalized tetrasubstituted Zn(II) phthalocyanines over porphyrin free base *meso*-functionalized analogues, such as **H_2_TOFP**, **H_2_OOFP**, or even **H_2_SOFP**, with the new **ZnTOFPc4** derivative presenting the most promising perspectives in this respect. In order to further improve their two-photon oxygen photosensitizing capability while preserving an optimal luminescence, this contribution also constitutes a strong incentive to explore related assemblies with peripheral antennae in which the 2-fluorenyl units are fully conjugated to the central phthalocyanine core. Work along these lines is in progress in our laboratory.

## Figures and Tables

**Table 1:** Photophysical properties of the new phthalocyanines **ZnTOFPc1**–**4**, **H_2_TOFPc2**, and **H_2_TOFPc4**, and of the reference **ZnPc** in THF at 293 K. Related data are also given for selected porphyrin dendrimers in CH_2_Cl_2_.

Compounds	*λ*_abs_ Fluorenyl (nm)	*λ*_abs_ N and B (nm)	*λ*_abs_ Q (nm)	*λ*_max_ (M^−1^·cm^−1^)	*λ*_em_ (nm)	*Ф*_F_*^a^*	*λ*_max_.*Ф*_F_*^b^*	*Ф*_Δ_*^c^*
**ZnPc**	-	343	602, 666	234,000	671, 741	0.26 *^d^*	60840	0.61
**ZnTOFPc1**	276	303, 350	611, 677	-	687, 758	0.29	-	-
**ZnTOFPc2**	276	306, 351	611, 677	215,000	688, 758	0.33	70950	0.57
**H_2_TOFPc2**	276	306, 343, 387	606, 644, 667, 703	136,000	709, 790	0.43	58480	-
**ZnTOFPc3**	270	307, 353	628, 698	270,000	706, 779	0.23	62100	0.60
**ZnTOFPc4**	309 (sh), 328, 344		609, 675	291,000	683, 753	0.37	107670	0.54
**H_2_TOFPc4**	310 (sh), 327, 344		604, 637, 665, 699	175,000	705, 786	0.47	82250	-
**H_2_TOFP *^e^***	304	423	516, 551, 590, 647	213,000	663, 728	0.10	21300	-
**H_2_OOFP *^e^***	304	423	516, 551, 592, 653	245,300	652, 721	0.13	31889	0.64

*^a^* Fluorescence quantum yield determined relative to **(Py)ZnPc** in a toluene/pyridine (99:1) mixture after excitation at 606 nm, *Ф*_F_ = 0.30 [28]. *^b^* One-photon brightness when excited in the Soret band. *^c^* Singlet oxygen formation in a toluene/pyridine (99:1) mixture relative to **ZnPc** (*Φ*_Δ_ = 0.61) in the same mixture [53,54]. *^d^* Literature value of *Ф*_F_ = 0.23 (± 0.03) determined via the comparative method using chlorophyll in diethylether as standard [29]. *^e^* In CH_2_Cl_2_ [22,25].

**Table 2:** Two-photon absorption of new phthalocyanines in THF at 20 °C.

Compounds	*λ*_2PA_^max2^ (nm)	*σ*_2_^max2 *a*^ (GM)	*σ*_2_^max2^*.Ф*_F_*^b^* (GM)	*λ*_2PA_^max1^ (nm)	*σ*_2_^max1 *a*^ (GM)	*σ*_2_^max1^*.Ф*_F_*^b^* (GM)
**ZnPc *^b^***	820	200	52	880	150 *^c^*	14
**ZnTOFPc2**	830	330	109	880	160	53
**H_2_TOFPc2**	840	350	150	880	130	56
**ZnTOFPc3**	860	160	37	880	140	32
**ZnTOFPc4**	830	510	189	880	240	89
**H_2_TOFPc4**	840	360	169	880	200	94
**H_2_OOFP *^d^***	790	45	5	-	-	-
**H_2_SOFP *^d^***	790	75	11	-	-	-

*^a^* Cross-sections derived by two-photon excited fluorescence (TPEF) in THF (*fs* regime). *^b^* Two-photon brightness at the various 2PA maxima. *^c^* In line with the value reported by Drobizhev et al. (*σ*_2_ = 150 GM in pyridine at 880 nm) [58]. *^d^* In CH_2_Cl_2_ [25].

## Supplementary Materials

The following are available online, ^1^H-NMR and ^13^C-NMR of the new compounds, selected emission and TPEF data, and selected DFT data (Cartesian coordinates, optimized GS geometries, TD-DFT outputs).
