# Supplementary material for: Phthalocyanine-Cored Fluorophores with Fluorene-Containing Peripheral Two-Photon Antennae as Photosensitizers for Singlet Oxygen Generation
Source: Molecules. 2020 Jan 7;25(2):239. doi: 10.3390/molecules25020239 (PMC7024215; doi:10.3390/molecules25020239)
Supplement: Supplementary file 1 [file molecules-25-00239-s001.pdf]

Supporting information:

Phthalocyanine-Cored Fluorophores with Fluorene-Containing Peripheral Two-photon Antennae as Photosensitizers for Singlet Oxygen Generation

Seifallah Abid,[a,b] Sarra Ben Hassine,[a,c] Nicolas Richy,[a] Franck Camerel,[a] Bassem Jamoussi,[d] Mireille Blanchard-Desce,[e] Olivier Mongin,[a] Frédéric Paul,[a] Christine O. Paul-Roth,*[a]

[a] *Univ Rennes, INSA Rennes, CNRS, ISCR (Institut des Sciences Chimiques de Rennes) – UMR 6226, F-35000 Rennes, France*
[b] *Université de Carthage, Faculté des Sciences de Bizerte, Tunisie*
[c] *Faculté des Sciences de Tunis, Université de Tunis El Manar, Tunisie*
[d] *Department of Environmental Sciences, Faculty of Meteorology, Environment and Arid Land Agriculture, King Abdulaziz University, Jeddah, Saudi Arabia*
[e] *Université de Bordeaux, Institut des Sciences Moléculaires (CNRS UMR 5255), 33405 Talence, France*

*Corresponding author: christine.paul@univ-rennes1.fr or christine.paul@insa-rennes.fr

tel : (+33) (0) 2 23 23 63 72

Contents:

1. NMR spectra of compounds **6**, **11** and **Pn1-4** in $CDCl_3$ P. 2

2. NMR Spectra of **ZnTOFPc1-4**, **H_2TOFPc2** and **H_2TOFPc4** in THF-d_8 P. 9

3. Complementary UV-vis and TPEF data on **ZnTOFPc1-4**, **H_2TOFPc2** and **H_2TOFPc4** P. 16

4. Complementary DFT data on **ZnTOFPc2'-4'** and **H_2TOFPc4'** P. 19

1. NMR spectra of compounds 6, 11 and Pn1-4 in $CDCl_3$

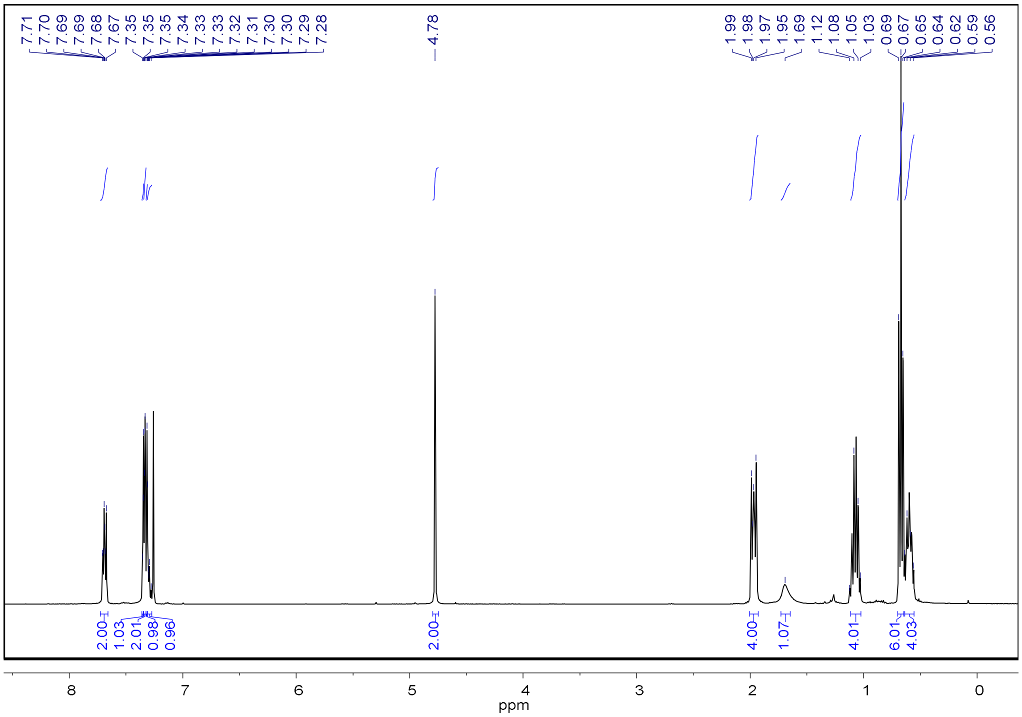

Figure S1: ^{1}H NMR Spectrum of compound **6** in $CDCl_3$.

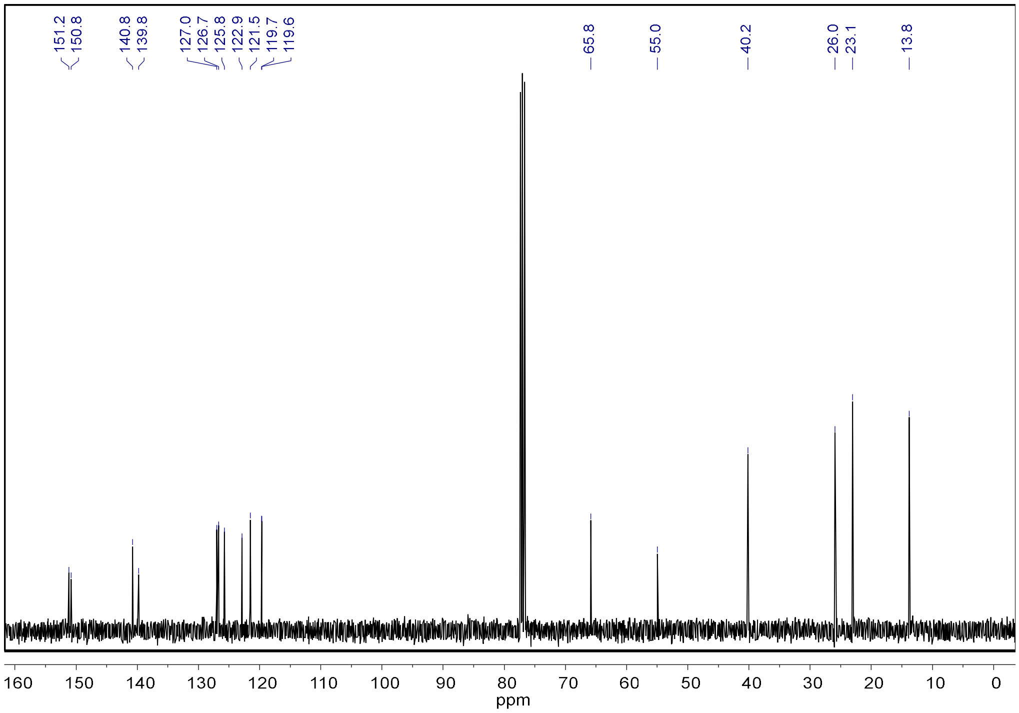

Figure S2: $^{13}C\{^{1}H\}$ NMR Spectrum of compound **6** in $CDCl_3$.

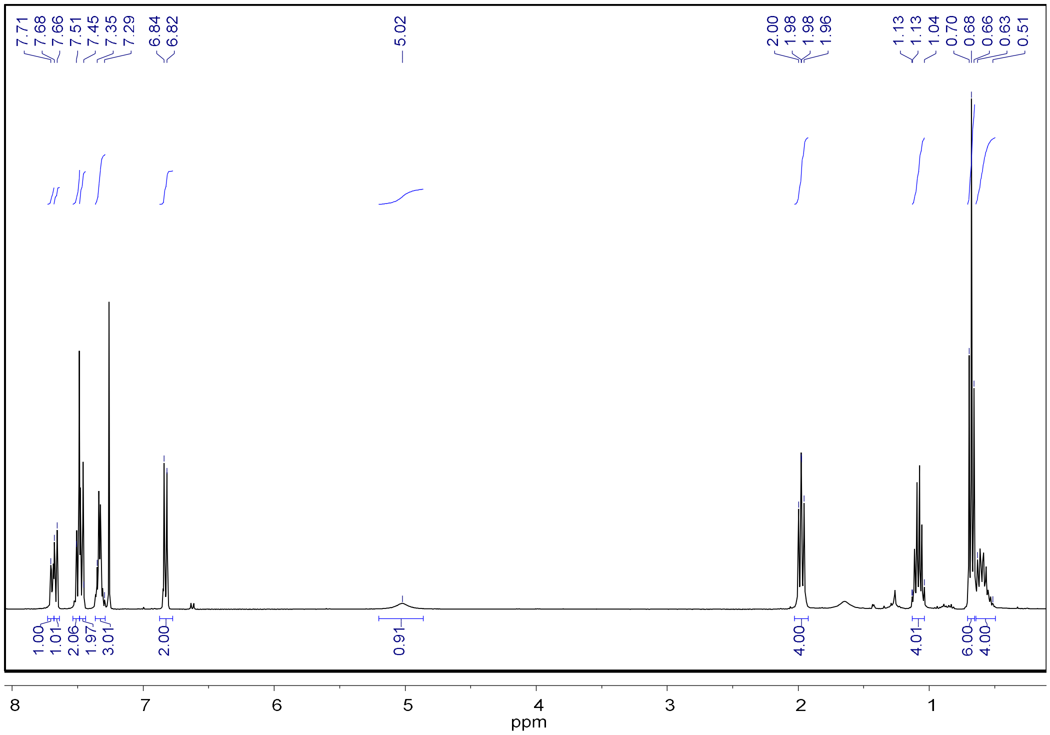

Figure S3: ^{1}H NMR Spectrum of compound **11** in $CDCl_3$.

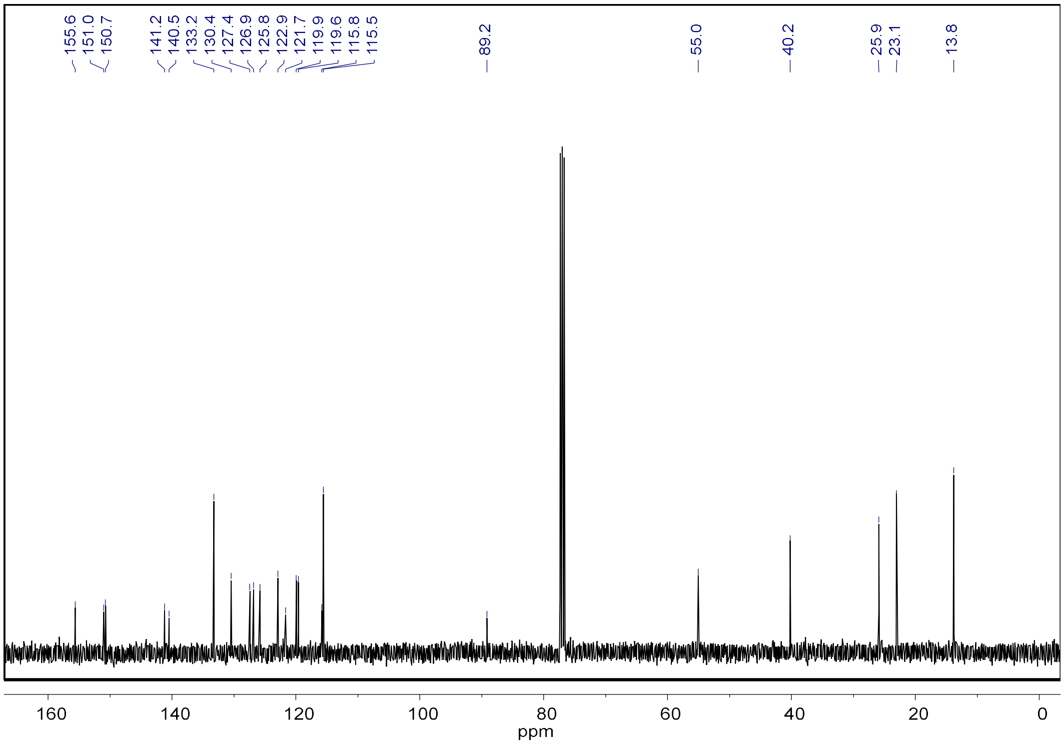

Figure S4: $^{13}C\{^{1}H\}$ NMR Spectrum of compound **11** in $CDCl_3$.

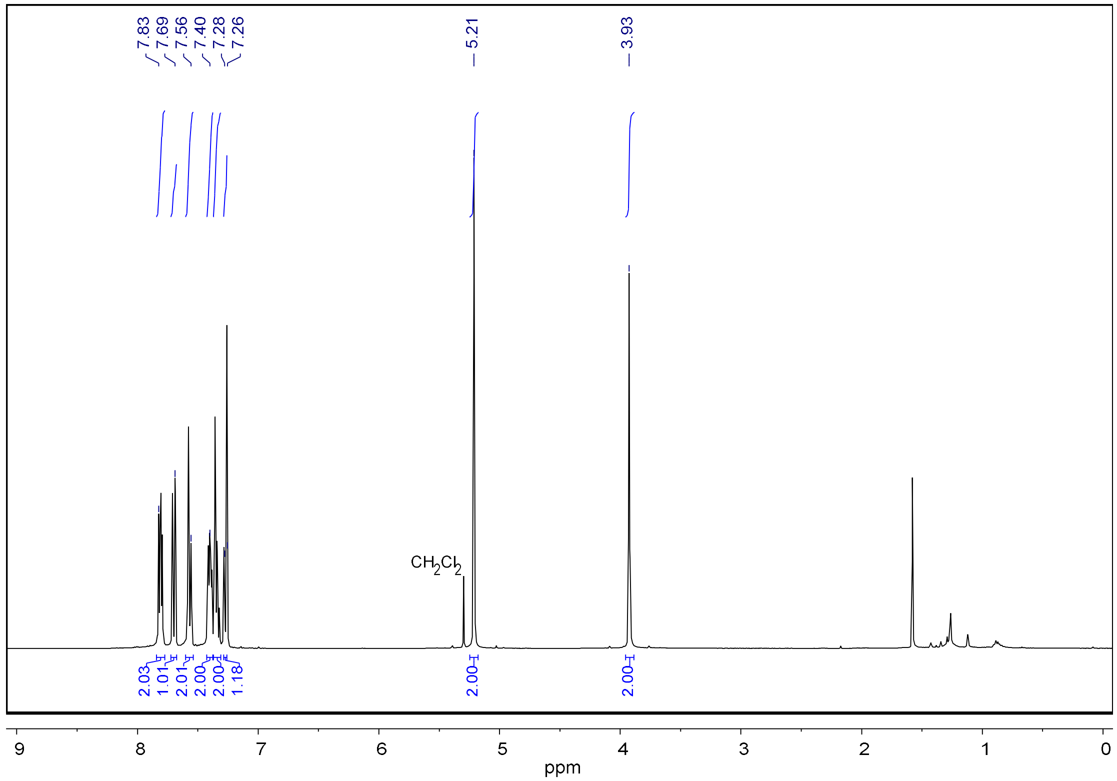

Figure S5: ^{1}H NMR Spectrum of phthalonitrile **Pn1** in $CDCl_3$.

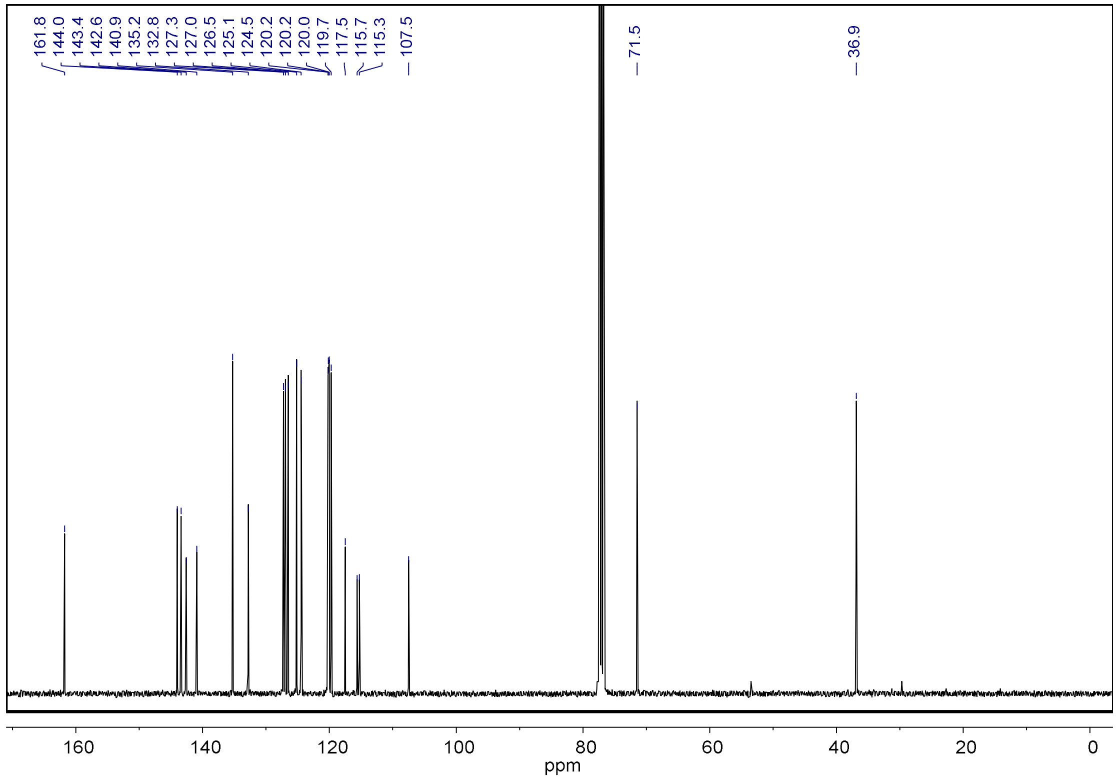

Figure S6: $^{13}C\{^{1}H\}$ NMR Spectrum of compound **Pn1** in $CDCl_3$.

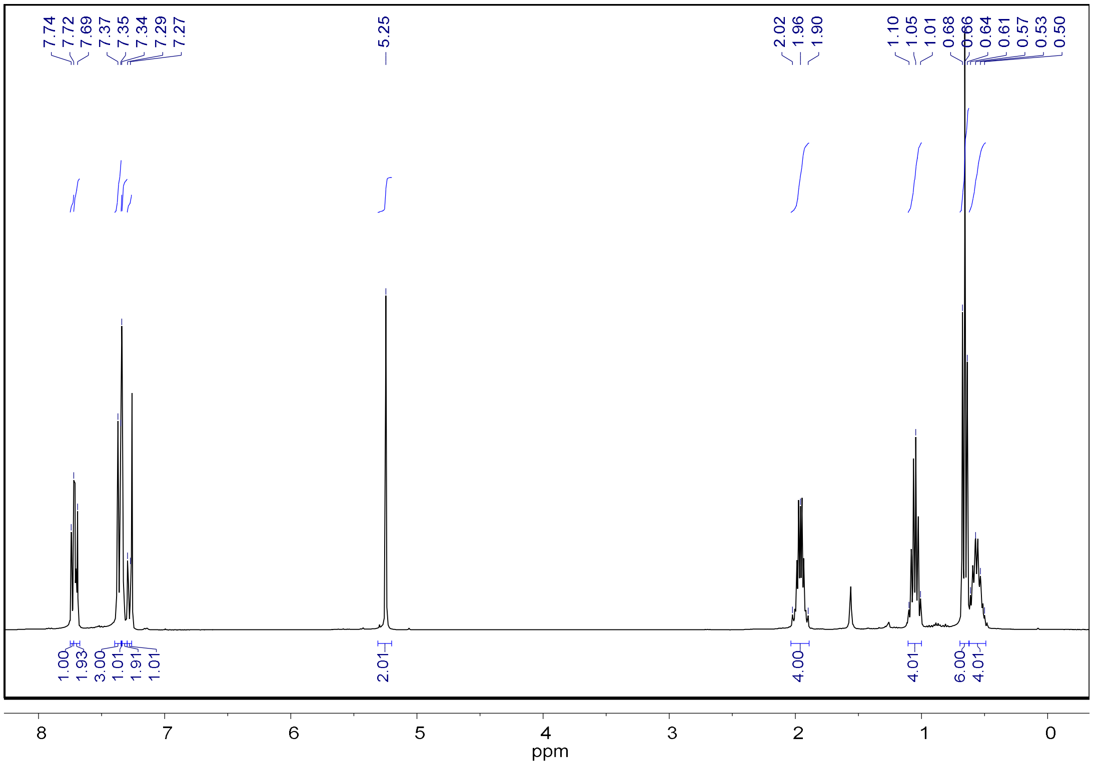

Figure S7: ^{1}H NMR Spectrum of phthalonitrile **Pn2** in $CDCl_3$.

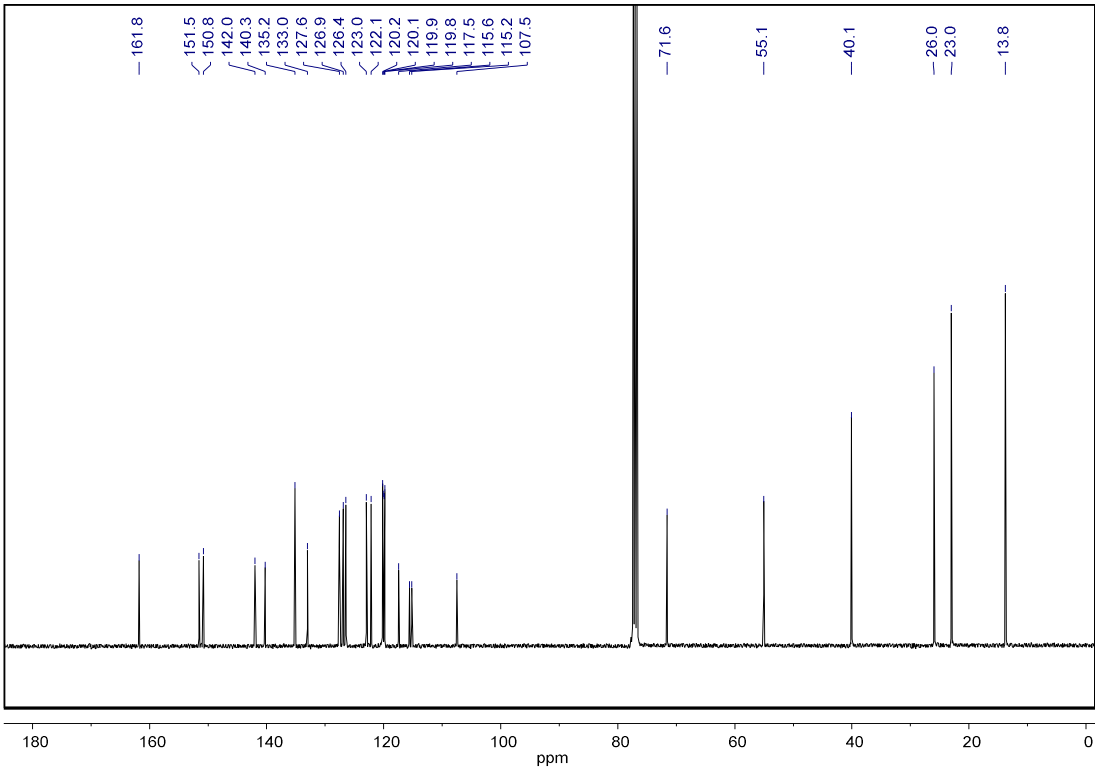

Figure S8: ^{13}C{^{1}H} NMR Spectrum of compound **Pn2** in $CDCl_3$.

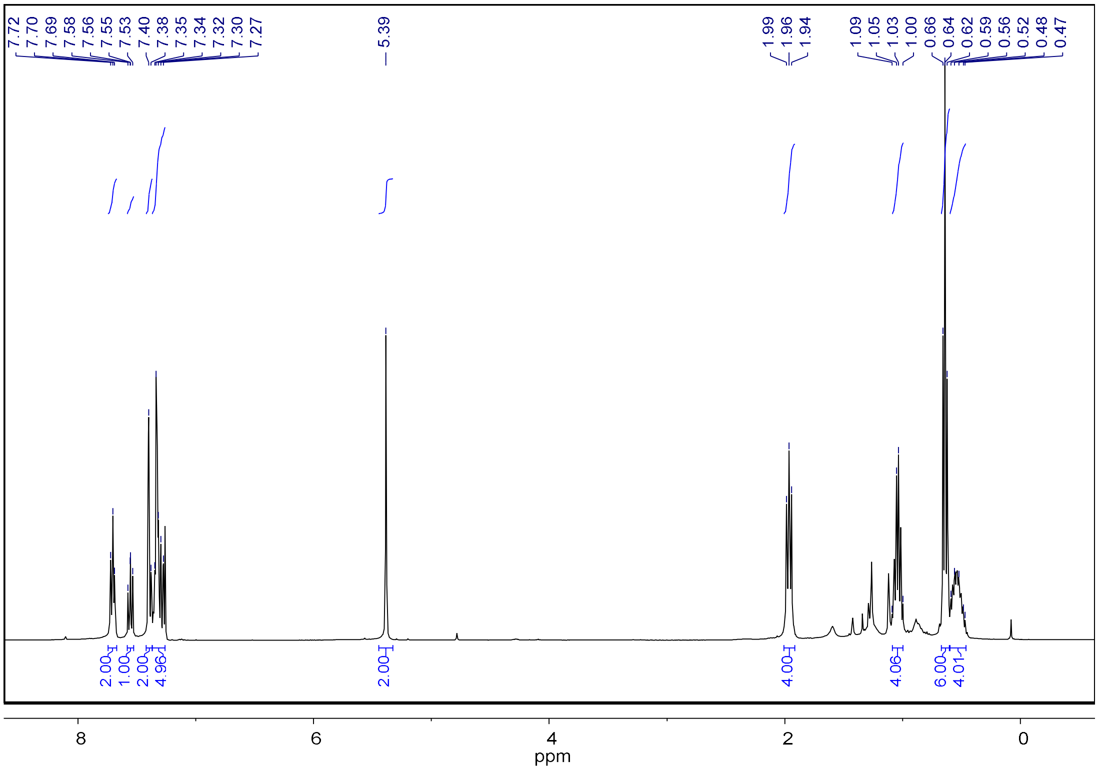

Figure S9: ^{1}H NMR Spectrum of phthalonitrile **Pn3** in $CDCl_3$.

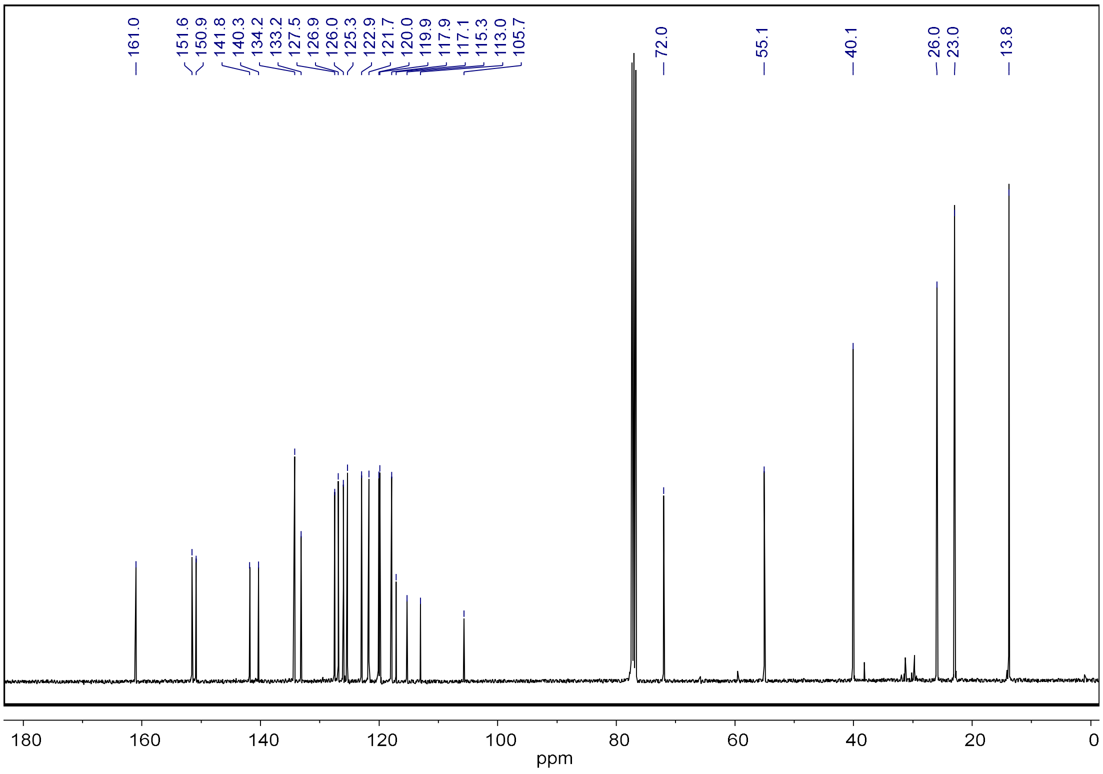

Figure S10: $^{13}C\{^{1}H\}$ NMR Spectrum of compound **Pn3** in $CDCl_3$.

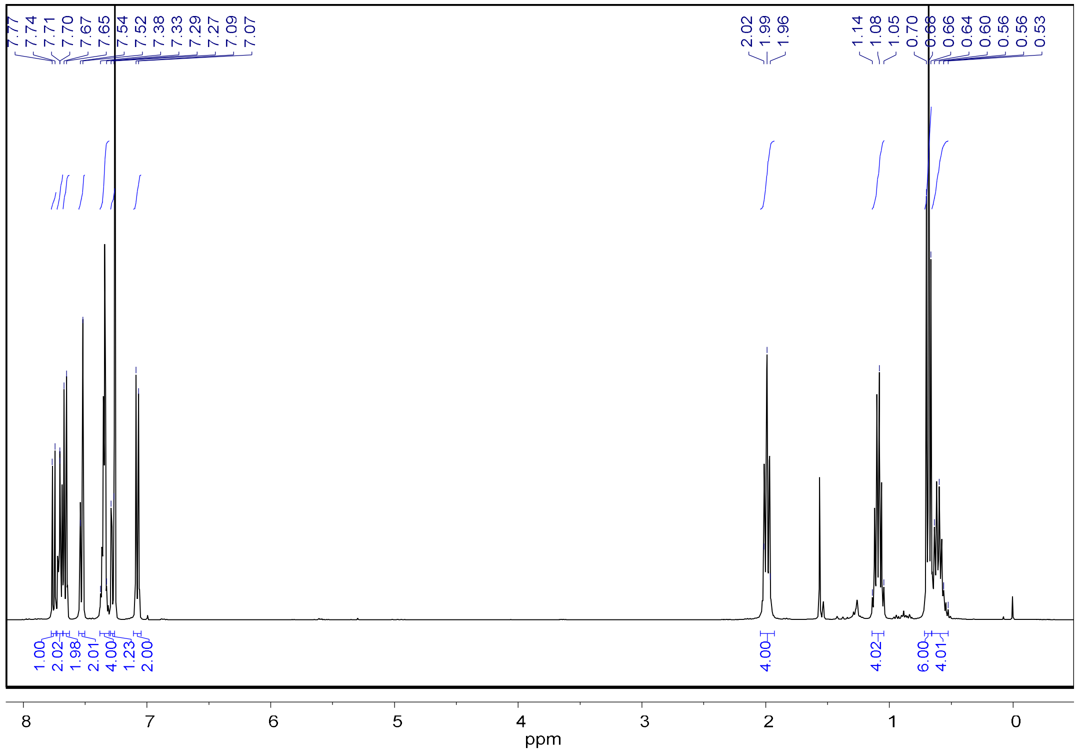

Figure S11: ^{1}H NMR Spectrum of phthalonitrile **Pn4** in $CDCl_3$.

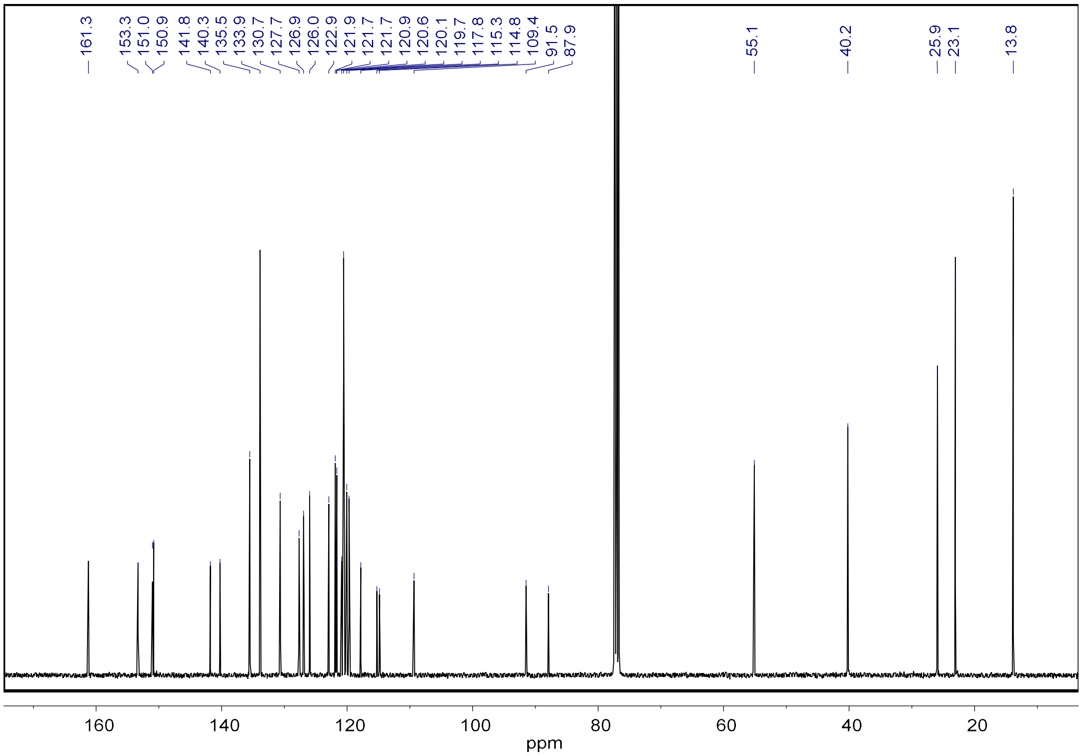

Figure S12: $^{13}C\{^{1}H\}$ NMR Spectrum of compound **Pn4** in $CDCl_3$.

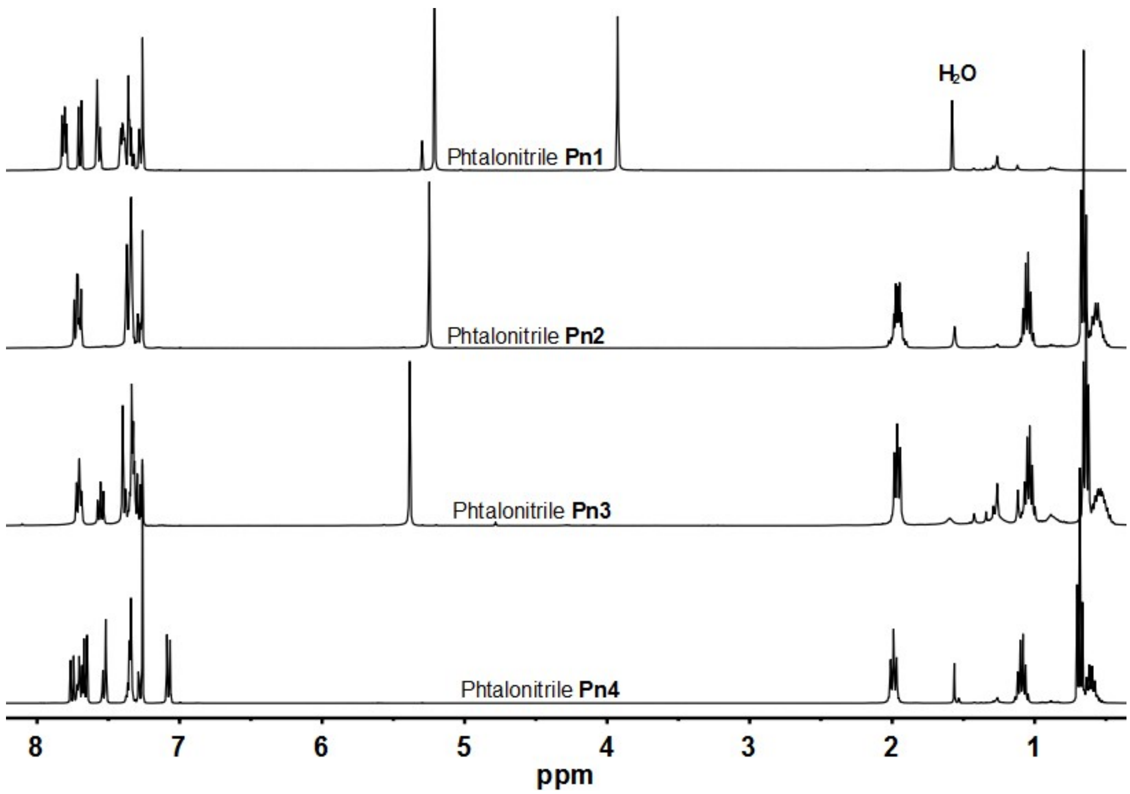

Figure S13: Comparison of ^{1}H NMR spectra of phthalonitriles **Pn1-4** in $CDCl_3$.

2. NMR Spectra of ZnTOFPc1-4, H_2TOFPc2 and H_2TOFPc4 in THF-d_8

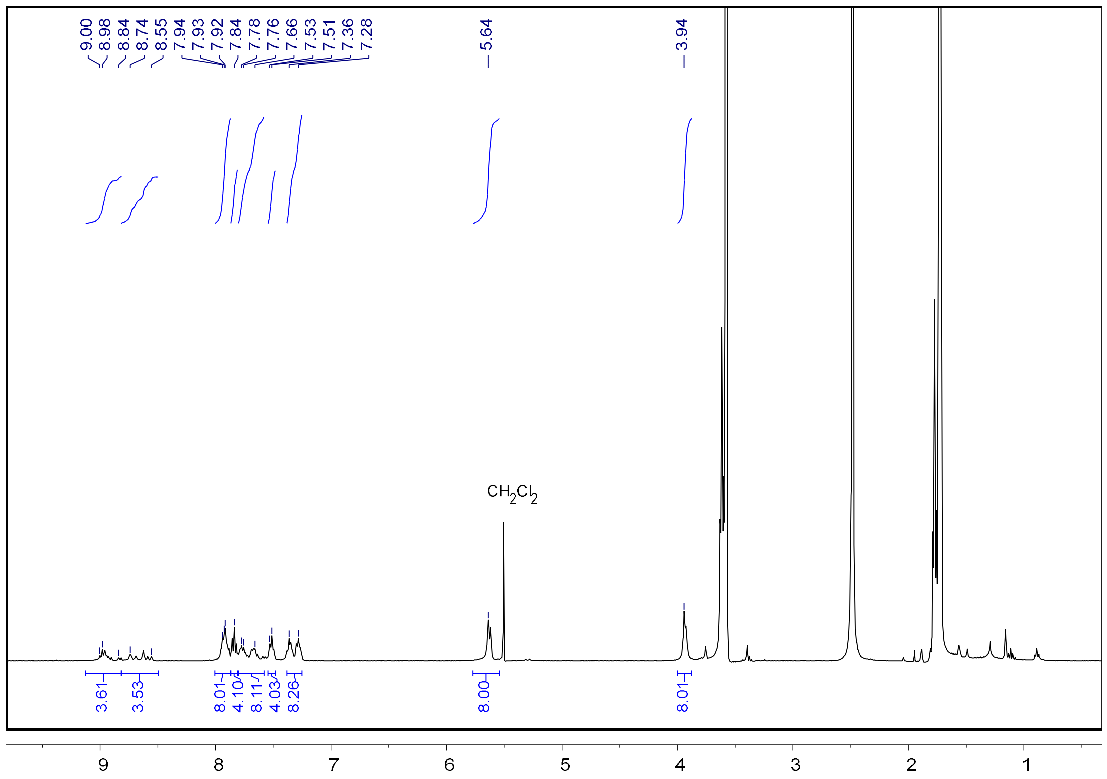

Figure S14: ^{1}H NMR Spectrum of phthalonitrile **ZnTOFPc1** in THF-d_8.

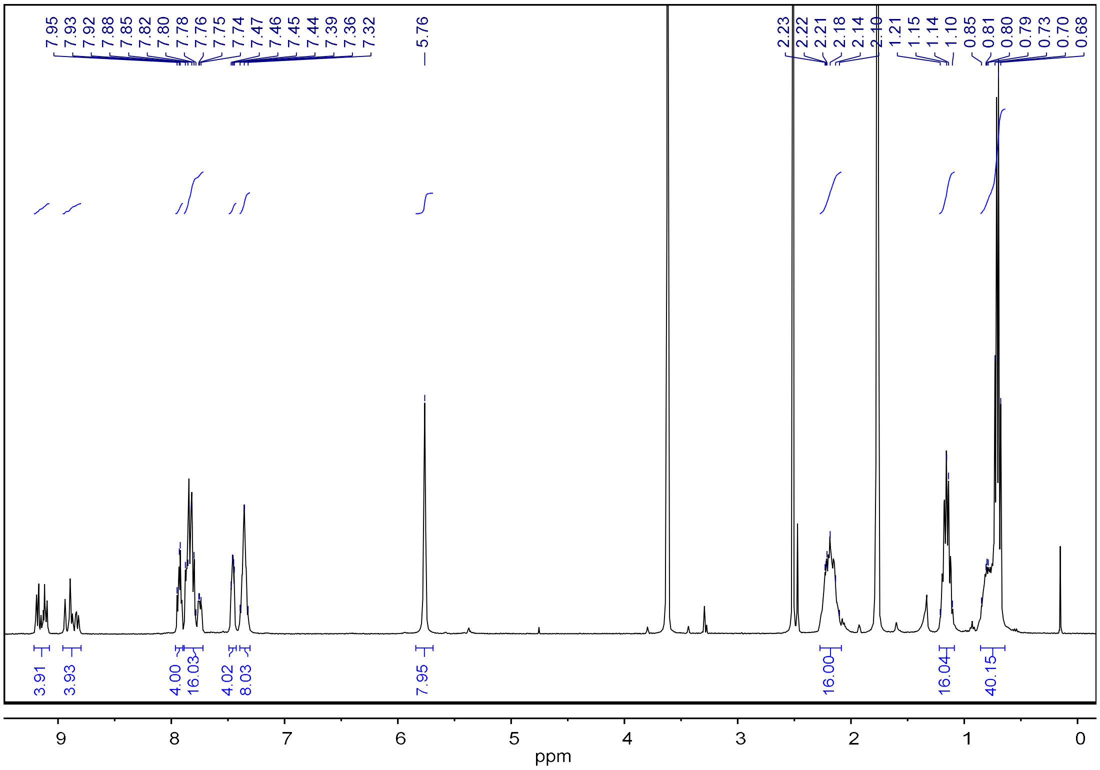

Figure S15: ^{1}H NMR Spectrum of phthalonitrile **ZnTOFPc2** in THF-d_8.

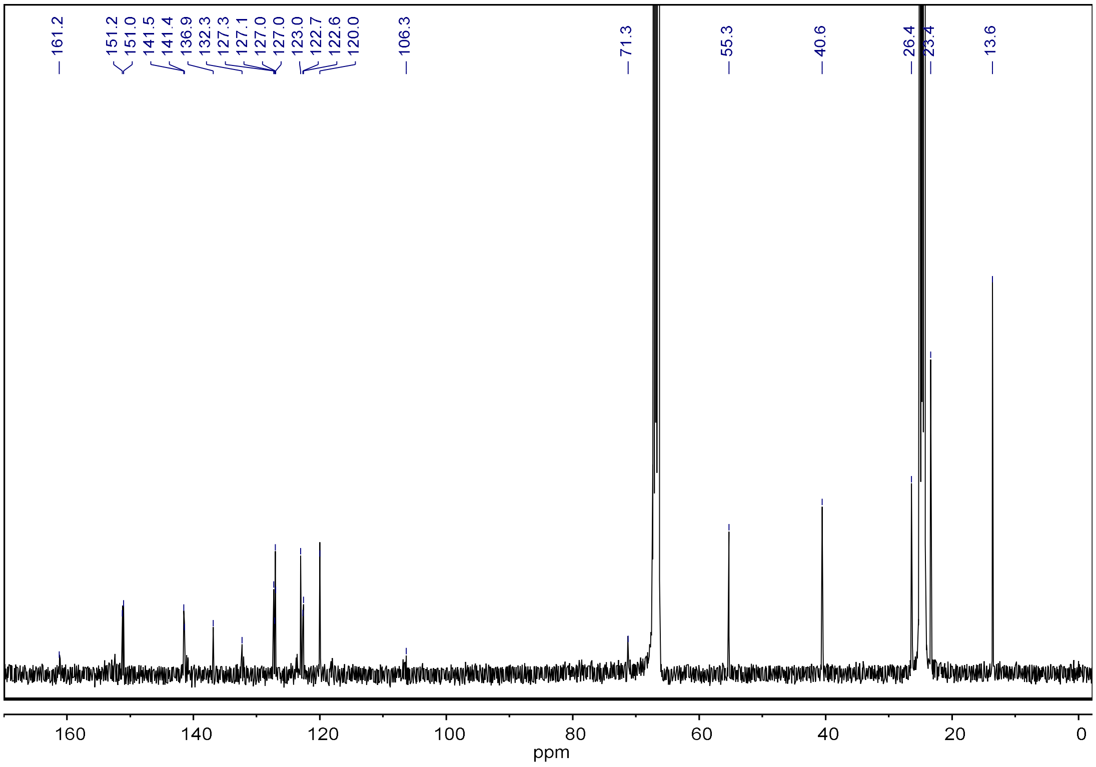

Figure S16: ^{13}C{^{1}H} NMR Spectrum of phthalonitrile **ZnTOFPc2** in THF-d_8.

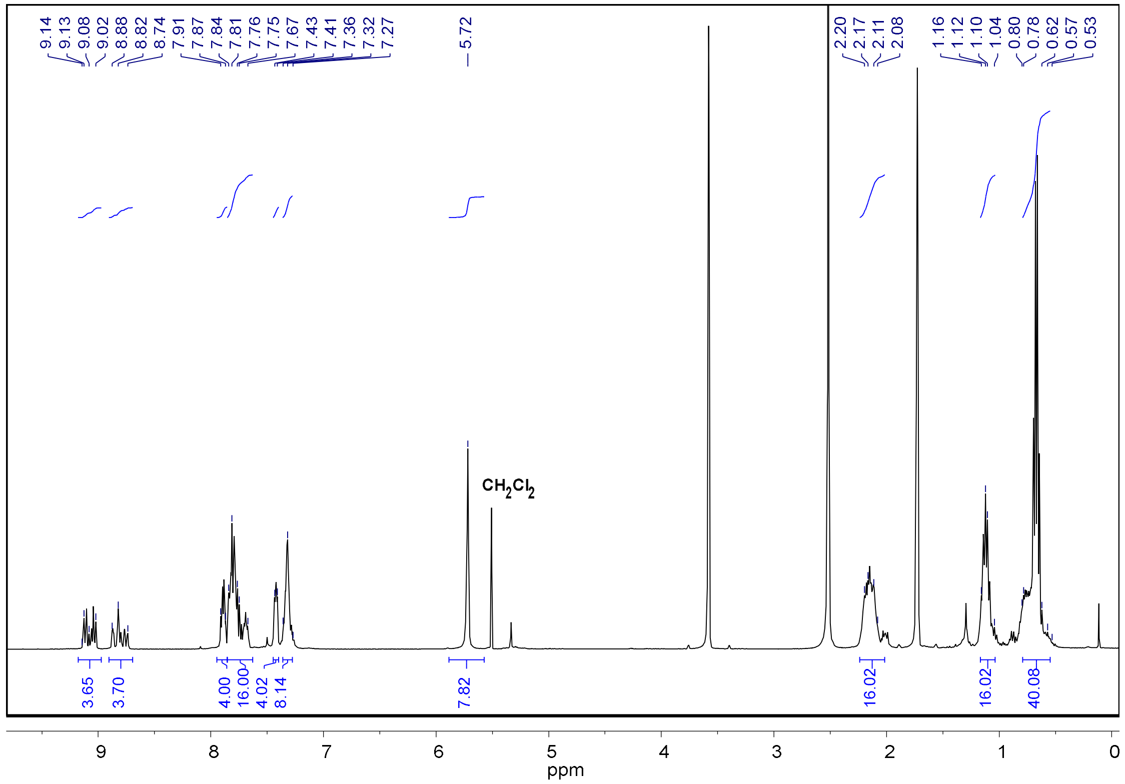

Figure S17: ^{1}H NMR Spectrum of phthalonitrile **ZnTOFPc3** in THF-d_8.

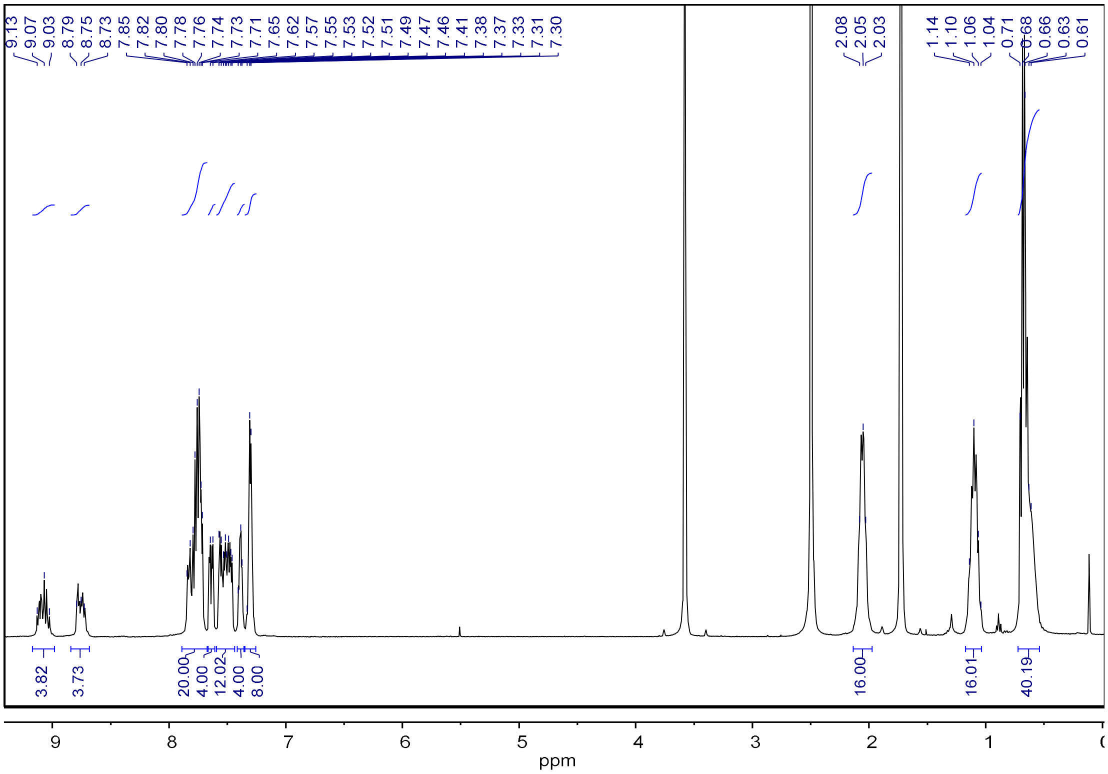

Figure S18: ^{1}H NMR Spectrum of phthalonitrile **ZnTOFPc4** in THF-d_8.

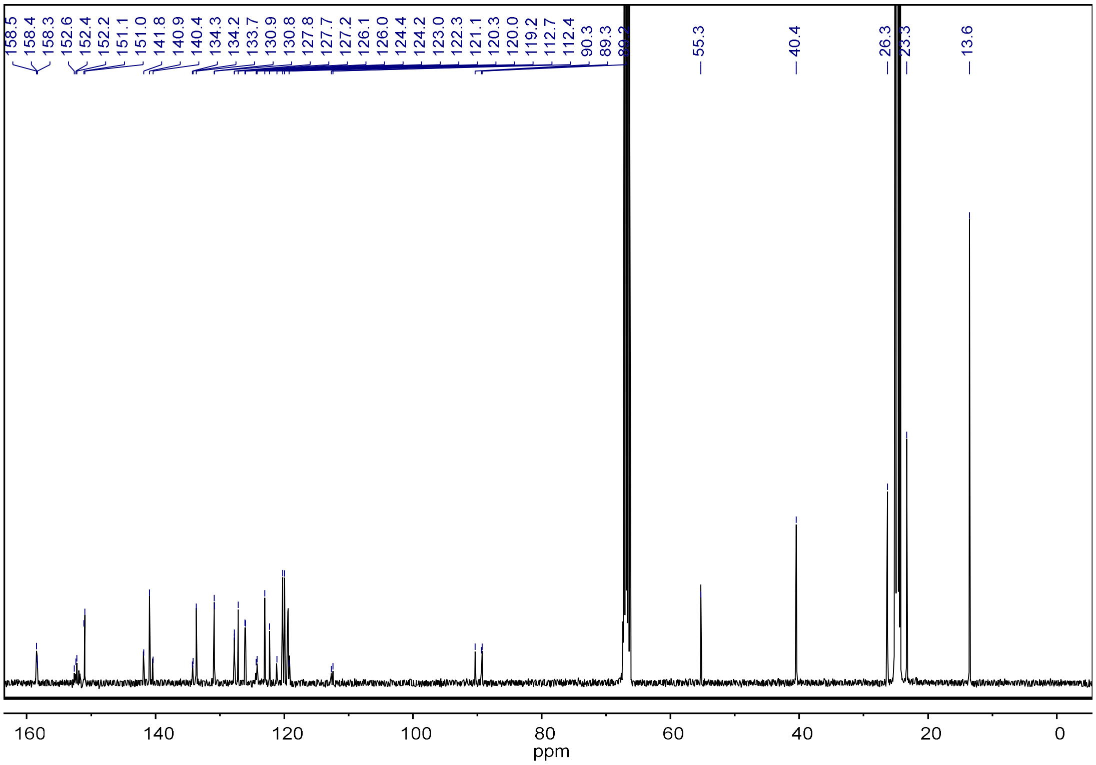

Figure S19: $^{13}C\{^{1}H\}$ NMR Spectrum of phthalonitrile **ZnTOFPc4** in THF-d_8.

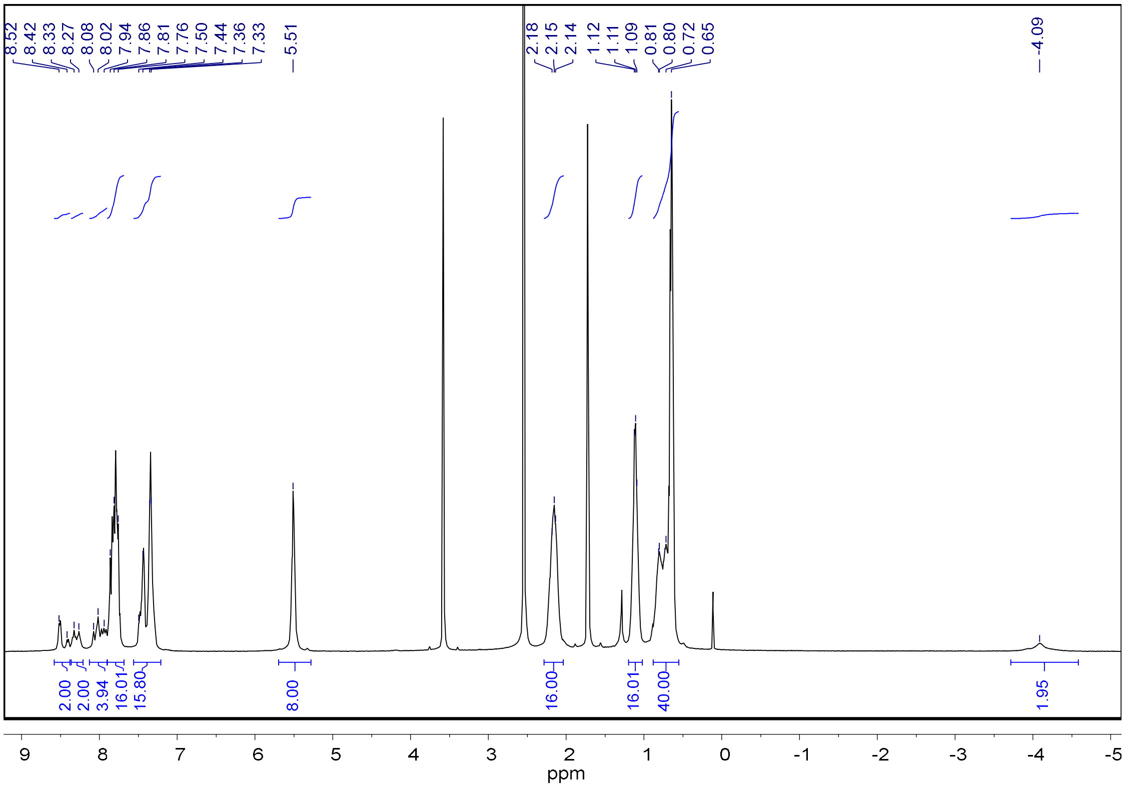

Figure S20: ^{1}H NMR Spectrum of phthalonitrile **H_2TOFPc2** in THF-d_8.

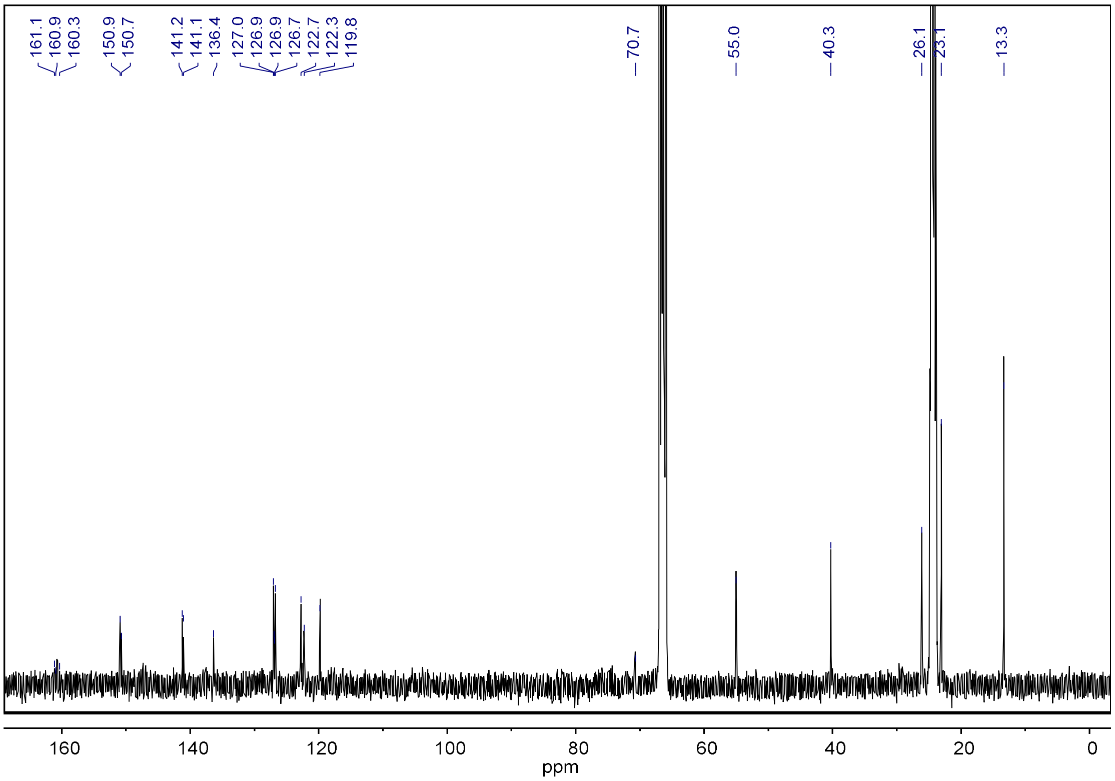

Figure S21: $^{13}C\{^{1}H\}$ NMR Spectrum of phthalonitrile **H_2TOFPc2** in THF-d_8.

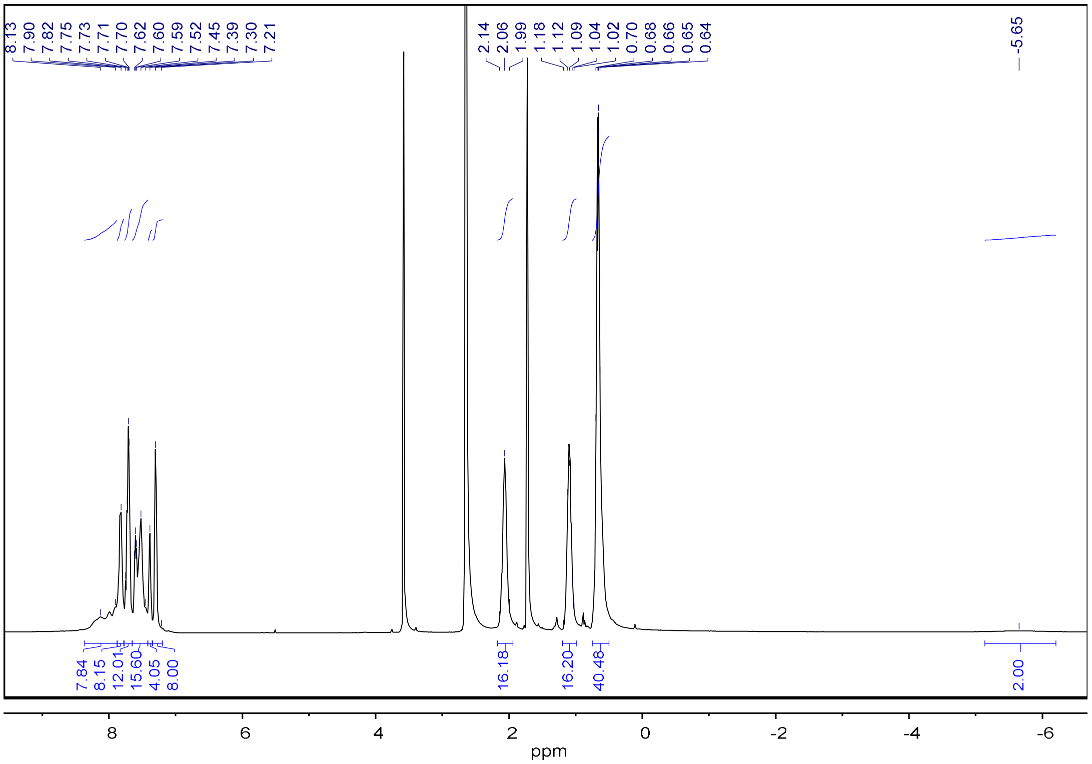

Figure S22: ^{1}H NMR Spectrum of phthalonitrile **H_2TOFPc4** in THF-d_8.

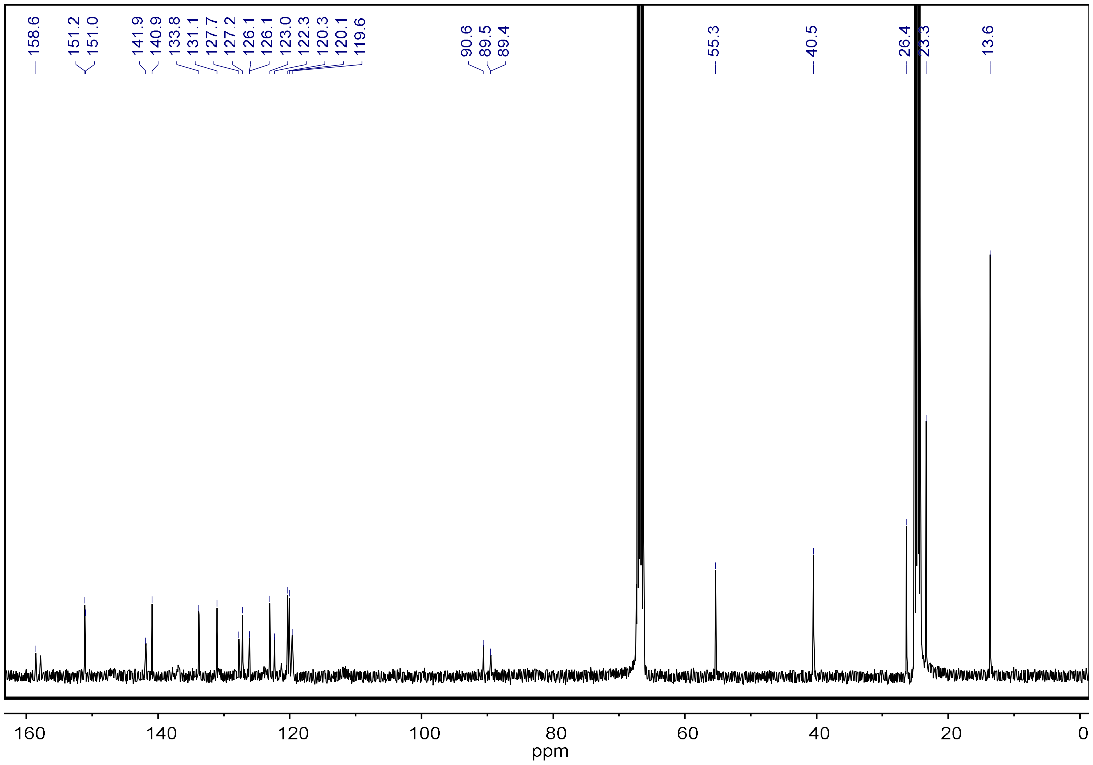

Figure S23: $^{13}C\{^{1}H\}$ NMR Spectrum of phthalonitrile **H_2TOFPc4** in THF-d_8.

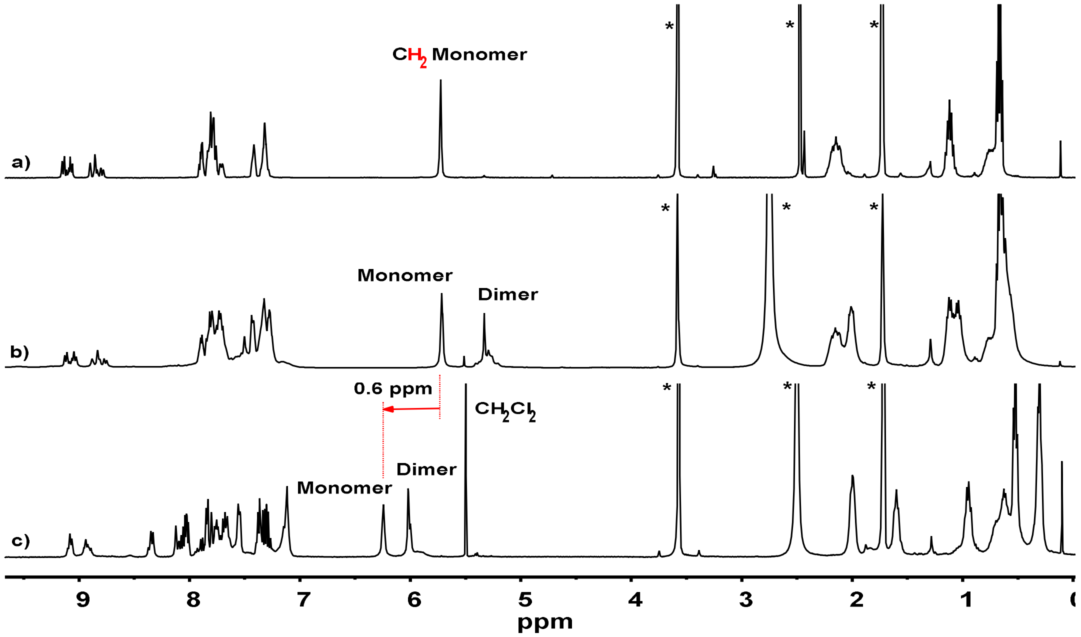

Figure S24: Comparative ^{1}H NMR Spectra of **ZnTOFPc2** in THF-d_8 (*) at different concentrations: a) C ≈ 6 mM, b) C ≈ 14 mM, c) C ≈ 22 mM.

3. Complementary UV-vis and TPEF data on ZnTOFPc1-4, H_2TOFPc2 and H_2TOFPc4

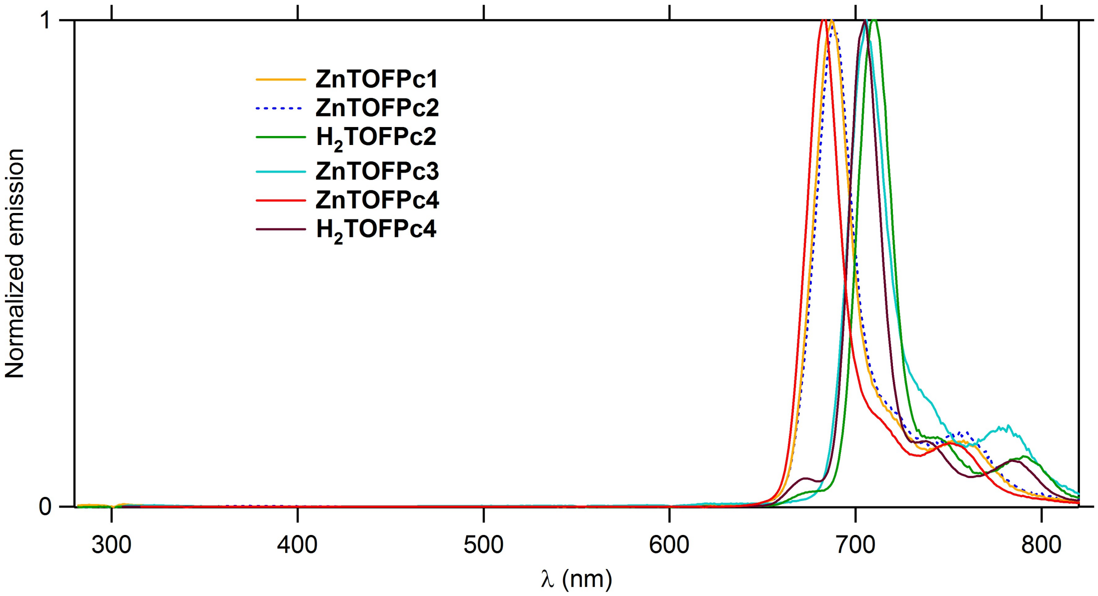

Figure S25: Emission spectra of **ZnTOFPc1-4** and **H_2TOFPc2** and **H_2TOFPc4** upon excitation within the fluorene-containing π- π* absorption band (276 or 310 nm; see Table 1).

(a)

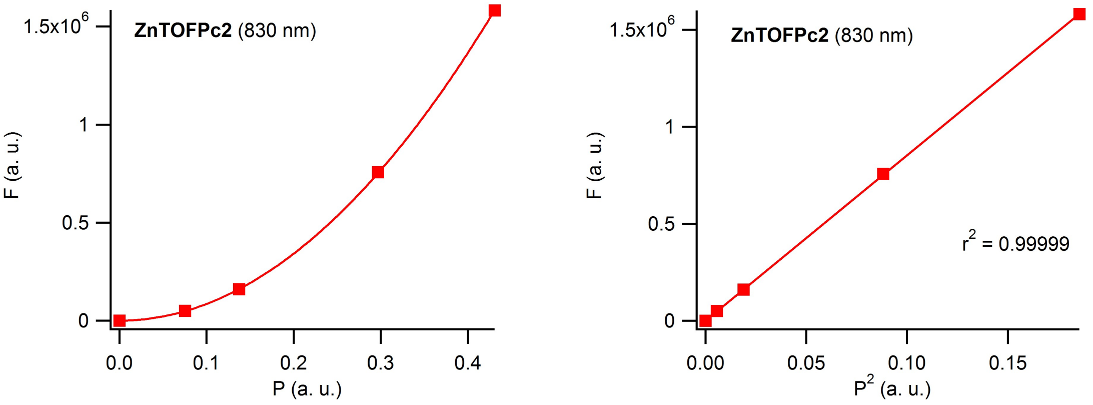

(b)

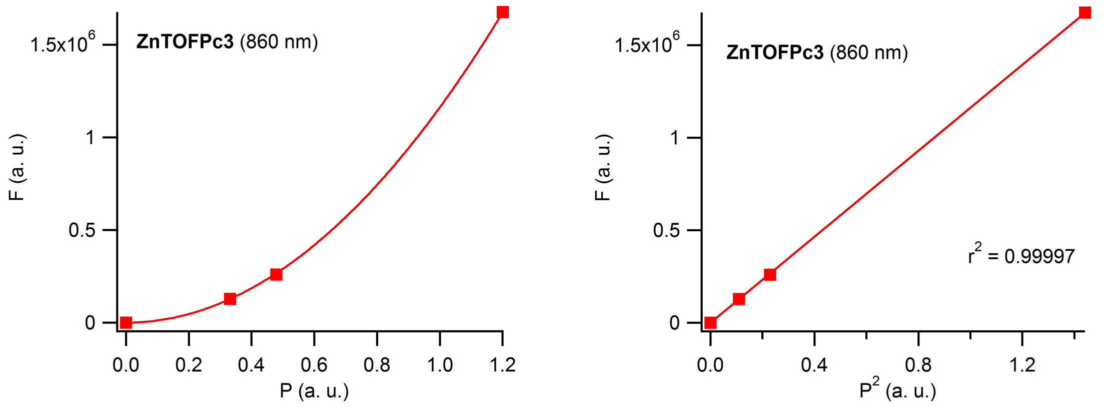
ZnTOFPc3 (860 nm)
1.5x10^6
1
0.5
0
F (a. u.)
0.0 0.2 0.4 0.6 0.8 1.0 1.2
P (a. u.)
ZnTOFPc3 (860 nm)
1.5x10^6
1
0.5
0
F (a. u.)
r^2 = 0.99997
0.0 0.4 0.8 1.2
P^2 (a. u.)

(c)

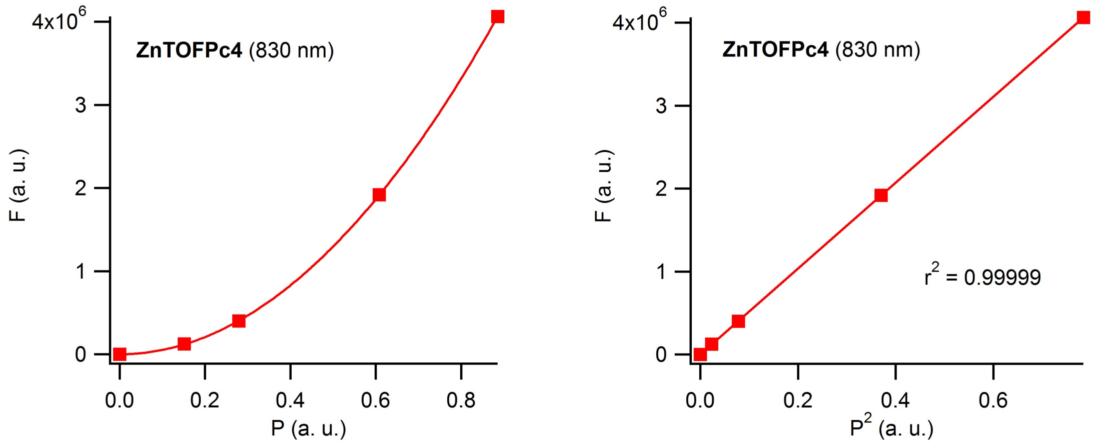
ZnTOFPc4 (830 nm)
4x10^6
3
2
1
0
F (a. u.)
0.0 0.2 0.4 0.6 0.8
P (a. u.)
ZnTOFPc4 (830 nm)
4x10^6
3
2
1
0
F (a. u.)
r^2 = 0.99999
0.0 0.2 0.4 0.6
P^2 (a. u.)

(d)

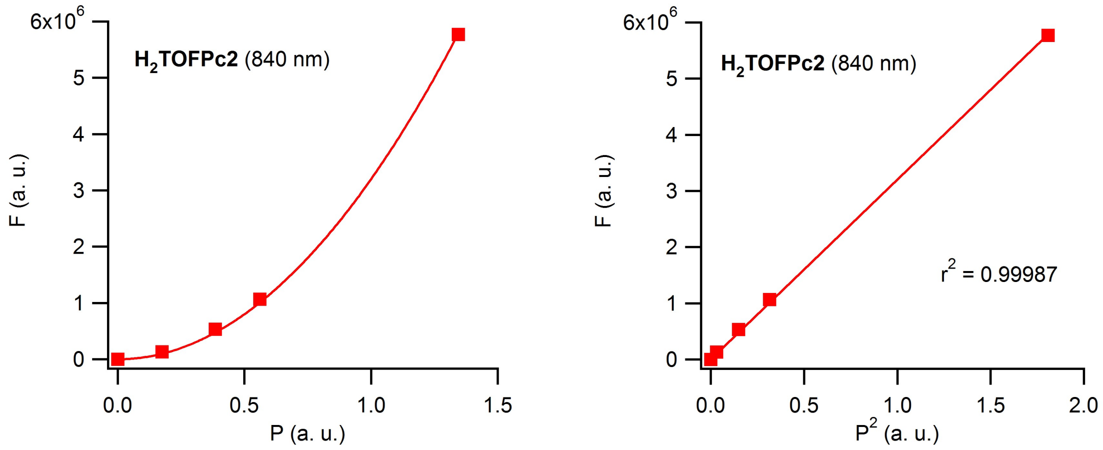
H2TOFPc2 (840 nm)
6x10^6
5
4
3
2
1
0
F (a. u.)
0.0 0.5 1.0 1.5
P (a. u.)
H2TOFPc2 (840 nm)
6x10^6
5
4
3
2
1
0
F (a. u.)
r^2 = 0.99987
0.0 0.5 1.0 1.5 2.0
P^2 (a. u.)

(e)

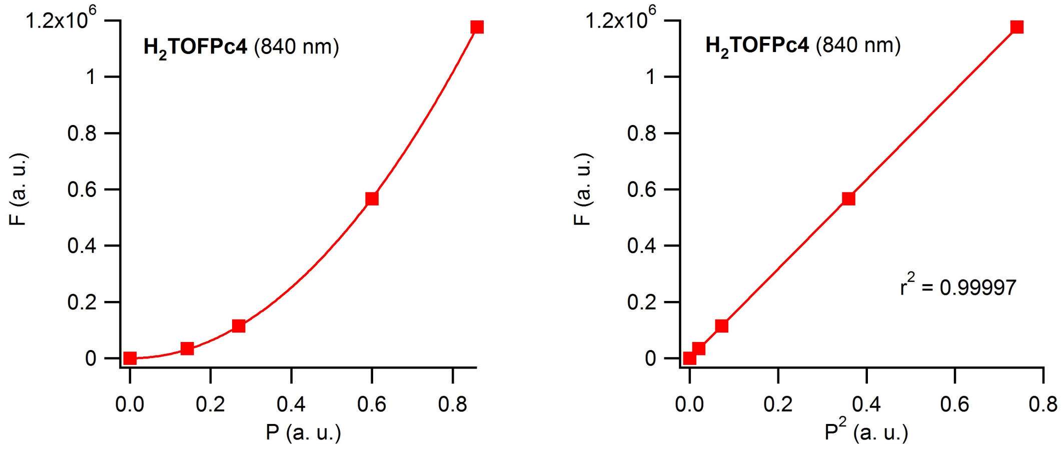

Figure S26: Left: quadratic dependence of the emission intensity (F) on laser excitation power (P) for compound **ZnTOFPc2-4**, **H_2TOFPc2** and **H_2TOFPc4** (a-e) at $(\lambda_{2PA})^{max2}$ nm. Right: dependence of F on P^2.

4. Complementary DFT data on ZnTOFPc2'-4' and H_2TOFPc4'

Table S1. Cartesian coordinates of **ZnTOFPc2'-4'** and **H_2TOFPc4'** after geometrical optimization.

a) ZnTOFPc2':

Atom	X	Y	Z
C	-3.10141500	-2.93246829	0.42802650
C	-1.96156382	-3.78884160	0.44552128
C	-0.78030459	-2.92748510	0.36994886
N	-1.20330223	-1.60275661	0.31291976
C	-2.59256281	-1.55562504	0.34487278
C	-2.92841243	3.12800786	0.14861346
C	-3.78359875	1.99170273	0.21263079
C	-2.92252275	0.80887967	0.23858616
N	-1.59418726	1.22885442	0.18963125
C	-1.54958960	2.61942170	0.13484518
N	-3.36540752	-0.45791020	0.31011435
C	3.13527911	2.95293034	-0.04625791
C	1.99658420	3.81044692	-0.03589996
C	0.81560135	2.94958139	0.04671174
N	1.23820603	1.62372720	0.08845099
C	2.62588248	1.57625224	0.03448784
N	-0.45389097	3.39161724	0.07254824
C	2.96291895	-3.10649329	0.24830252
C	3.81785963	-1.97118356	0.15622182
C	2.95685893	-0.78798397	0.12949631
N	1.62880335	-1.20691144	0.20113990
C	1.58445575	-2.59649949	0.27429295
N	0.48971235	-3.36862143	0.35290921
N	3.39910387	0.47802427	0.05524785
C	4.43179439	3.46094804	-0.15399199
C	4.58045329	4.85917846	-0.25017359
C	3.45233128	5.72507256	-0.23866535
C	2.15298489	5.19924266	-0.13345530
C	-3.43932925	4.42828322	0.12144691
C	-4.84037329	4.58174976	0.15530265
C	-5.70463039	3.45181362	0.21349169
C	-5.17595157	2.15089885	0.24315538
C	3.47469503	-4.40450290	0.30479149
C	4.87671503	-4.55798791	0.26719941
C	5.73991318	-3.43099458	0.17854126
C	5.20896642	-2.13082212	0.12257068
C	-4.39971479	-3.44466216	0.46029972

C	-4.55048293	-4.84619745	0.51598423
C	-3.42197774	-5.70991486	0.53293943
C	-2.12031723	-5.17925850	0.49602923
Zn	0.01757435	0.01064712	0.20067412
C	-6.88841773	7.67915083	0.49574308
C	-7.58588795	8.23022597	1.59197428
C	-7.79416444	9.62003323	1.69539955
C	-7.28927611	10.45763710	0.68897602
C	-6.57932355	9.90912927	-0.41275089
C	-6.38031877	8.53167914	-0.51331608
C	-7.34232676	11.92234363	0.52913051
C	-6.66460687	12.26760511	-0.66947736
C	-6.11780627	11.01608785	-1.37269130
C	-7.92535192	12.91439513	1.33604688
C	-7.82385954	14.26162537	0.93597479
C	-7.14952871	14.60618871	-0.25488076
C	-6.56681830	13.60623077	-1.06446894
C	-4.56693223	11.05635178	-1.47927582
C	-6.73917985	10.84285506	-2.78666848
O	-5.29695208	5.90075406	0.10407864
C	-6.71439038	6.18161007	0.38710086
C	-7.63016198	-6.96258986	0.58231883
C	-8.14502081	-8.19346736	0.11649742
C	-9.52198219	-8.47540379	0.18428956
C	-10.38790445	-7.50687067	0.71883819
C	-9.87552027	-6.26642464	1.17860344
C	-8.50691714	-5.99146880	1.11427767
C	-11.84979793	-7.51092136	0.91013757
C	-12.22905857	-6.27145458	1.48913099
C	-11.00468461	-5.37018042	1.70841070
C	-12.81368693	-8.49140928	0.61889077
C	-14.16585985	-8.22319754	0.91166958
C	-14.54377860	-6.99148114	1.48734047
C	-13.57216033	-6.00845968	1.77916626
C	-10.80486696	-5.03571367	3.21346582
C	-11.11433098	-4.05329871	0.88917606
O	-5.87134588	-5.29592791	0.55667565
C	-6.13246797	-6.73340684	0.53915647
C	6.98095273	-7.63899485	0.23175689
C	7.97616596	-8.21480945	1.05011526
C	8.22384139	-9.60151700	1.02435905
C	7.45402079	-10.41117671	0.17508968
C	6.44382735	-9.83829466	-0.64364915
C	6.20901351	-8.46291970	-0.62118967
C	7.47372782	-11.86700698	-0.05544322

C	6.47468579	-12.18315160	-1.01286381
C	5.73014668	-10.91926455	-1.46902117
C	8.27839089	-12.87508525	0.50273408
C	8.07650373	-14.20865573	0.09503203
C	7.08379828	-14.52419259	-0.85700719
C	6.27716239	-13.50828726	-1.41605724
C	4.21761374	-10.99290475	-1.11438088
C	5.90047518	-10.67782971	-2.99514592
O	5.32992998	-5.87636560	0.33334535
C	6.77180590	-6.14203343	0.24377549
C	7.65815446	6.95956831	-0.59896330
C	8.12238679	8.27459197	-0.83529947
C	9.49730363	8.54925836	-0.94002627
C	10.41338904	7.49131312	-0.80766260
C	9.95095687	6.17227824	-0.57235816
C	8.58257647	5.90208288	-0.46759222
C	11.88614928	7.46892877	-0.87105786
C	12.32232500	6.13237060	-0.67298557
C	11.12688836	5.19062669	-0.46346025
C	12.81460484	8.50271198	-1.08196278
C	14.18869982	8.19008004	-1.09401984
C	14.62316540	6.86204597	-0.89740061
C	13.68712499	5.82547267	-0.68540984
C	11.17320142	4.51800798	0.93776742
C	11.05936084	4.09876613	-1.56788312
O	5.89923590	5.30729003	-0.35396440
C	6.16106545	6.73654571	-0.48378864
H	5.30305841	2.81563844	-0.16468438
H	3.58501487	6.79913743	-0.30738667
H	1.28602419	5.85286521	-0.12437793
H	-2.79413849	5.29869889	0.07835240
H	-6.78025728	3.59135727	0.22906454
H	-5.82634872	1.28252565	0.28737078
H	2.83152165	-5.27437562	0.37787140
H	6.81580064	-3.56624197	0.15808463
H	5.85943394	-1.26375403	0.05672712
H	-5.27312441	-2.80242223	0.44157836
H	-3.55316313	-6.78568146	0.57457889
H	-1.25265175	-5.83201277	0.50852378
H	-7.96246360	7.57163022	2.37226958
H	-8.33225404	10.02861072	2.54729837
H	-5.82721006	8.09931461	-1.34327756
H	-8.44652045	12.65416094	2.25458904
H	-8.26787957	15.04370828	1.54746392
H	-7.07925174	15.65069813	-0.54963696

H	-6.04949674	13.88449016	-1.98025114
H	-4.18794684	10.12493426	-1.91892891
H	-4.24527860	11.88925746	-2.11788908
H	-4.10735630	11.18170144	-0.49192210
H	-6.45987872	11.68319472	-3.43550859
H	-6.37845734	9.91860382	-3.25614031
H	-7.83342331	10.79897325	-2.73359630
H	-7.31877303	5.77393710	-0.43856720
H	-7.01350491	5.68265895	1.31863050
H	-7.46678941	-8.93430741	-0.30459597
H	-9.90161580	-9.42694380	-0.18007656
H	-8.09840627	-5.04480451	1.45173341
H	-12.52868381	-9.44264910	0.17505291
H	-14.92611499	-8.96960205	0.69318750
H	-15.59147838	-6.79952447	1.70711366
H	-13.87504652	-5.06206989	2.22220500
H	-9.89690019	-4.43539655	3.35541857
H	-11.65617885	-4.45812405	3.59636233
H	-10.71100208	-5.94917933	3.81257316
H	-11.96240600	-3.45145583	1.24100018
H	-10.20139497	-3.45499063	1.00206580
H	-11.25915653	-4.26259584	-0.17720835
H	-5.69740003	-7.17875887	-0.36784161
H	-5.64625489	-7.19481337	1.41520593
H	8.55676119	-7.57789033	1.71490467
H	8.99366231	-10.03004299	1.66140561
H	5.43107729	-8.01021079	-1.23040065
H	9.04490392	-12.63760690	1.23692522
H	8.68900841	-15.00262253	0.51602222
H	6.94043675	-15.55837348	-1.16148821
H	5.51495628	-13.76347124	-2.14917364
H	3.71713419	-10.04869998	-1.36426435
H	3.72924777	-11.79658947	-1.68061828
H	4.07190066	-11.18616335	-0.04514256
H	5.44122469	-11.49418277	-3.56750465
H	5.41347638	-9.74030458	-3.29322700
H	6.96021406	-10.61897144	-3.27030737
H	7.15204648	-5.68063453	-0.68193609
H	7.28964881	-5.68458849	1.09856684
H	7.40485318	9.08793524	-0.93826495
H	9.83734976	9.56600772	-1.12171771
H	8.20833269	4.89993945	-0.29001659
H	12.48570086	9.52829728	-1.23393265
H	14.92197253	8.97686136	-1.25590124
H	15.68719512	6.63724248	-0.90949430

H	14.03403629	4.80533166	-0.53460770
H	10.27621471	3.90669212	1.09971660
H	12.05063964	3.86370678	1.02220243
H	11.22614343	5.26930706	1.73453946
H	11.94101517	3.44630497	-1.52027496
H	10.16697014	3.47374342	-1.43513458
H	11.01857212	4.54920945	-2.56663384
H	5.64684423	7.12667830	-1.37595165
H	5.75959201	7.25881238	0.39978038

b) ZnTOFPc3':

Atom	X	Y	Z
C	-1.81521006	-2.56574070	4.77424321
C	-0.88200362	-3.39049529	5.46376039
C	-1.07889168	-1.37351441	4.33224923
C	0.41205776	-2.69945090	5.42608958
C	10.22443038	7.16743903	11.19290417
C	-3.15821235	-2.99097592	4.64114634
C	11.19346584	7.51664209	10.22810133
C	6.97511770	4.74178854	9.10830803
C	-3.52487494	-4.22691792	5.22308339
C	-1.23661852	-4.62156685	6.03464188
C	-0.91982649	0.75979696	3.26531979
C	2.74342941	-2.61049718	5.94939349
C	-2.57570032	-5.02438114	5.90578061
C	8.58543694	3.05299515	10.11939996
C	-1.53934873	1.88464073	2.55416101
C	3.97536058	-3.15631092	6.53423939
C	-5.42626908	-2.62190533	3.75077131
C	9.29815291	6.13195747	10.93774903
C	11.25182086	6.83722729	8.99487026
C	9.35293016	5.45523116	9.71394727
C	10.84571902	4.87843538	6.34893168
C	8.46492470	4.30786101	9.20912085
C	-6.10673662	-1.64913255	2.81904401
C	-0.51280002	2.83996635	2.31023358
C	0.72563298	2.28107611	2.86977725
C	5.01174174	-2.22298554	6.25006703
C	4.40110504	-1.11564169	5.50466447
C	-7.17025896	-0.84342642	3.27882033
C	10.32853023	5.80727506	8.74454434
C	10.15136719	4.93604160	7.56822211
C	9.06799208	4.05311517	7.81966120
C	-0.82568714	4.04663900	1.64104701

C	-2.86789621	2.08128056	2.15055426
C	4.27552552	-4.33753066	7.25336656
C	6.34084379	-2.42546254	6.65033332
C	-4.07602947	-9.39460213	10.53120649
C	-5.68581830	-1.55166969	1.47107420
C	3.06915883	2.34179817	3.34106370
C	-2.16206958	4.24720481	1.22273595
C	-3.16139341	3.27818447	1.47705248
C	5.61489801	-4.54961292	7.65472899
C	6.62588967	-3.60549396	7.35536273
C	-6.32641397	-0.65839371	0.61147504
C	4.37509497	3.00392617	3.24603981
C	-3.54216106	-10.32649681	9.61540801
C	-7.39626845	0.15024480	1.08186209
C	6.54654965	11.16933192	2.47158039
C	6.52037469	11.00040288	1.07052914
C	8.67998435	3.11325084	6.86199300
C	-0.64668286	-6.82001453	12.05893565
C	-1.71299662	-5.51398746	10.15516864
C	-3.34182812	-8.24375719	10.89390035
C	-2.26829241	-10.12291582	9.04837945
C	2.44459284	10.02426188	4.08407945
C	5.31926351	2.16013174	3.89719951
C	3.90470804	7.95947105	4.34870254
C	-0.12220527	6.24857052	0.78744108
C	-7.87087646	0.99140075	-0.03255274
C	-6.04787978	-0.38925269	-0.87489294
C	3.52636352	-6.44655261	8.28561770
C	4.57373321	0.99122941	4.38621028
C	4.73313399	4.21946778	2.64651397
C	1.10984375	7.11818432	0.84171407
C	-7.82284865	0.05982821	2.41637164
C	-7.09273740	0.69401911	-1.18223990
C	2.22009482	-7.16035911	8.53180202
C	5.56563786	10.56189364	3.28633614
C	6.67850666	2.54606033	3.94872650
C	6.08613374	4.59176225	2.71361526
C	1.78635032	7.47331586	-0.34485822
C	-8.89328342	1.95488973	-0.08617310
C	-7.33410716	1.35976613	-2.38934118
C	-6.28984925	-1.66421745	-1.73298452
C	-4.60004696	0.12768261	-1.10317915
C	1.93940018	-8.37934007	7.87798220
C	5.51519980	10.22192673	0.46281726
C	10.44618369	3.93372975	5.38337492

C	-2.07633800	-8.03832926	10.33276831
C	0.72639887	-9.06028917	8.10054595
C	4.56559895	9.78819655	2.68625678
C	2.92954926	8.29625670	-0.31071283
C	7.04660722	3.77404413	3.35411570
C	1.58428876	7.59308354	2.08793751
C	-9.13323720	2.62338521	-1.30328618
C	-8.35996202	2.32906575	-2.44677986
C	1.27110210	-6.61047717	9.42649252
C	9.37323215	3.04962185	5.63088525
C	-1.09692032	-6.87913024	10.57113244
C	-1.54068610	-8.97612908	9.41142356
C	2.71664159	8.40764449	2.12979728
C	0.06659740	-7.27852772	9.65135777
C	3.40077831	9.03955595	3.35127542
C	4.54120624	9.61812009	1.27698446
C	9.00412240	1.99794505	4.59653281
C	-0.20883797	-8.50505279	8.98849378
C	3.39220928	8.76138647	0.93053958
H	-8.55739181	2.85339288	-3.37899181
H	-6.74476653	1.14136353	-3.27729539
H	-7.30586968	-2.05072972	-1.58954994
H	-5.57783359	-2.45290613	-1.45796021
H	-3.86749676	-0.63839380	-0.81898099
H	-4.39764452	1.02571272	-0.50879835
H	-9.92017399	3.37164353	-1.36442670
H	-9.49224428	2.18571774	0.79184483
H	6.41455490	5.52755551	2.26760899
H	1.06717204	7.30101188	2.99929460
H	1.48850181	-5.66315586	9.91529207
H	8.07381985	4.11759800	3.39985160
H	6.36739611	3.93373539	8.68186875
H	-4.44155368	0.37146153	-2.16174842
H	-6.15341074	-1.44227035	-2.79927807
H	3.98342688	4.83179241	2.15619612
H	4.00882764	-6.16407912	9.23362842
H	4.21884625	-7.07615865	7.70973114
H	-8.63887119	0.67503373	2.78741965
H	-7.48327868	-0.91622479	4.31847764
H	-0.95939692	6.71572322	1.32807793
H	9.45595462	1.03163398	4.84721510
H	9.35091828	2.28615990	3.59414611
H	-0.43114230	6.06295266	-0.25060482
H	7.99666602	2.22250759	9.70939433
H	9.62853028	2.72666163	10.20708193

H	-4.85428441	-2.16348699	1.12851702
H	7.10261116	-1.69128156	6.41075895
H	7.64232618	-3.81016003	7.68331911
H	5.88914714	-5.44749607	8.19805098
H	-3.62208764	1.32976812	2.35950071
H	-4.17674257	3.47704716	1.14221635
H	1.42223717	7.09715508	-1.29886113
H	3.44044741	8.55870269	-1.23385983
H	2.66933394	-8.79219517	7.18462781
H	0.52458469	-9.99704937	7.58694540
H	-2.44164229	5.15281420	0.69510492
H	8.55568950	5.87245737	11.68935895
H	10.19179686	7.70128679	12.13994153
H	11.89936225	8.31637951	10.44033263
H	12.00064662	7.11023544	8.25481583
H	4.44180094	8.42692856	5.18425740
H	2.96471925	10.52076990	4.91343723
H	-2.58163621	-5.27634581	10.78297098
H	-1.50297210	-6.60564715	12.71139586
H	-5.40959391	-3.63463345	3.32004745
H	5.49967099	10.09495086	-0.61726428
H	7.28275596	11.47558447	0.45742797
H	7.32931974	11.77244004	2.92592871
H	5.59710596	10.69858753	4.36508072
H	4.58104428	7.24884617	3.86046197
H	3.06027160	7.39378121	4.76244735
H	1.58317178	9.48536492	4.49901721
H	2.07183944	10.79643103	3.40056131
H	-1.86178841	-10.84454118	8.34345714
H	-4.11995037	-11.20803144	9.34716361
H	-5.06063174	-9.56595391	10.96027404
H	-3.76354390	-7.53240384	11.60071631
H	-2.03779197	-5.52515860	9.10853484
H	-0.97893927	-4.70688694	10.27335419
H	0.09684827	-6.02584389	12.20481215
H	-0.20275891	-7.77120127	12.37584424
H	7.84863779	2.43438782	7.03436074
H	-5.93421066	-2.66015770	4.72443111
H	6.57517417	4.97860195	10.10277226
H	8.20707451	3.27137945	11.12644258
H	-0.49380073	-5.22301137	6.54830583
H	-2.90087452	-5.97061339	6.33185153
H	-4.54726529	-4.58288096	5.15577356
H	11.67382892	5.55230354	6.14257457
H	10.97299885	3.88417887	4.43133614

H	6.86349602	5.62757387	8.47200515
N	5.08679562	-0.04576060	5.06846195
N	-1.59470561	-0.33078577	3.66108076
N	0.24996814	-1.50259939	4.74169790
N	0.42931201	1.03908546	3.43550058
N	3.04922425	-1.38697926	5.34833942
N	1.93794311	2.85650776	2.83298234
N	1.53362055	-3.19185997	5.97501456
N	3.23295649	1.14454903	4.02394435
O	0.19694266	4.96387398	1.43978991
O	-4.03444177	-2.17117304	3.94238581
O	7.57175172	1.68107815	4.57021582
O	3.23558710	-5.21688173	7.52225899
Zn	1.73992078	-0.17578638	4.38829237

c) ZnTOFPc4':

Atom	X	Y	Z
C	1.64164643	-13.77764319	-5.71295207
C	1.29593383	-15.06405006	-5.21454793
C	2.76242024	-13.64470312	-6.58093140
C	0.86896839	-12.63044465	-5.34455919
C	3.50166500	-14.77422085	-6.92821419
C	3.14665174	-16.05470454	-6.42492471
C	4.09590951	-17.04696866	-6.95619459
C	5.03230095	-16.37397834	-7.78472000
C	2.04220970	-16.19957734	-5.56660958
C	4.73659113	-14.86758634	-7.83740743
C	4.17748553	-18.43714735	-6.76113345
C	6.05342127	-17.08833016	-8.42106824
C	5.20669870	-19.15296806	-7.40382711
C	6.13810747	-18.48504842	-8.22769364
C	0.21009303	-11.64167622	-5.03320354
C	-0.56096306	-10.49231826	-4.66633625
C	-1.63323291	-10.60125940	-3.74151637
C	-0.27668414	-9.21760806	-5.22288861
C	-2.38534041	-9.47633911	-3.37798632
C	-2.07652296	-8.22413133	-3.93688171
C	-1.02852117	-8.08906153	-4.86781390
C	12.64771027	1.40812800	8.13625943
C	-2.47533324	-5.84264664	-3.40803801
C	-1.18377217	-5.53826611	-2.94023910
C	-0.88759935	-4.18354691	-2.73671908
C	-1.84655386	-3.16162323	-2.97963265
C	0.33544243	-3.51685691	-2.26831079

C	-1.20136572	-1.88606184	-2.65829400
C	-3.44391259	-4.83545549	-3.65023935
C	-3.13650399	-3.48435920	-3.43426263
C	12.75919162	-0.73823227	9.49798966
C	3.24917003	14.26314845	-7.23193743
C	2.60079086	-3.56526691	-1.50792145
C	3.82946507	-4.27744537	-1.14766832
C	0.83718784	13.53374656	-6.87935821
C	4.77831406	-3.29362566	-0.75449253
C	4.16884174	-5.64111970	-1.14231252
C	6.07515797	-3.63800155	-0.34947004
C	6.39402097	-5.00834947	-0.33764007
C	5.46006662	-6.00084138	-0.73033434
C	-15.95784129	1.00816806	-5.23112499
C	8.54899255	-4.82492944	0.85993077
C	-14.95081439	-1.05828892	-6.32126794
C	-1.21441592	0.49633734	-2.46810112
C	-1.88172939	1.80074957	-2.58181727
C	4.41350136	-14.40226020	-9.28541990
C	-0.93440558	2.78415054	-2.18350572
C	0.29654433	2.07240488	-1.83026929
C	-3.17682301	2.14620990	-2.99136911
C	-3.50199020	3.51502567	-2.98210786
C	-2.56693160	4.50761944	-2.59293254
C	5.92307206	-14.03792066	-7.27011545
C	-5.93587174	3.27262997	-3.33061418
C	-1.27201763	4.14816789	-2.19201150
C	16.75374054	1.36240653	10.10872566
C	17.67572913	0.81741084	9.18926278
C	2.56257114	2.02430859	-1.07190046
C	3.78654006	2.69112579	-0.60594056
C	4.74691068	1.66941851	-0.36796159
C	4.10195496	0.39407712	-0.69018215
C	15.36497888	1.17954105	9.92649120
C	4.07787001	4.04464343	-0.38730815
C	5.37239884	4.34918186	0.07217621
C	6.03228980	1.99064708	0.10059991
C	6.33986243	3.34146679	0.31719390
C	17.22547427	0.08306119	8.07443936
C	5.23670787	6.80007227	-0.21139853
C	13.47091899	0.11874554	8.41286430
C	-6.85036115	3.42034921	-4.38768978
C	-6.26408176	2.50696834	-2.19518066
C	-8.09889269	2.78814200	-4.31611242
C	-8.45172649	1.99826095	-3.18999784

C	-7.51282534	1.87339072	-2.13222062
C	-9.72558229	1.34838473	-3.11860194
C	-10.81514131	0.78569004	-3.04807616
C	-12.08667280	0.13253228	-2.97479129
C	-12.43019821	-0.63866046	-1.83021841
C	-13.01955953	0.24538534	-4.04451915
C	-14.25148104	-0.40043333	-3.95413815
C	-14.58411842	-1.16829756	-2.80575371
C	-15.93380860	-1.72723320	-2.99034313
C	-16.42689119	-1.30261137	-4.25229283
C	-13.67190516	-1.28722575	-1.74208319
C	-15.39875351	-0.42019831	-4.97604195
C	-16.71196316	-2.54614865	-2.15295808
C	-17.69907664	-1.69603908	-4.68168990
C	-17.99250087	-2.93999205	-2.58871114
C	-18.48337056	-2.51902936	-3.84331539
C	5.04460033	7.91858258	0.61756566
C	4.98986794	6.87718351	-1.59558675
C	4.59099358	9.12218946	0.06152012
C	4.32151222	9.22579210	-1.32900441
C	4.53204955	8.08290334	-2.14474763
C	3.85663840	10.45632717	-1.89455967
C	3.45895417	11.51567700	-2.37240623
C	2.99819584	12.74806588	-2.93602709
C	2.77593745	13.87774691	-2.10095708
C	2.75881839	12.85809533	-4.33529909
C	2.31269244	14.06818923	-4.86400727
C	2.09405478	15.19039314	-4.02028332
C	1.63053041	16.31622012	-4.84839482
C	1.56671437	15.88358142	-6.19923591
C	2.32582136	15.09463333	-2.63650346
C	1.99404630	14.41369752	-6.32670920
C	1.28036238	17.63209730	-4.49752371
C	1.15344145	16.76505001	-7.20420415
C	0.86429024	18.51658495	-5.51210808
C	0.80112513	18.08771420	-6.85541845
C	9.91688574	-4.88835741	0.54295116
C	8.11246122	-4.20844966	2.04840428
C	10.85617255	-4.31900193	1.41294250
C	10.44380287	-3.67609880	2.61025343
C	9.05701467	-3.63601107	2.91156656
C	11.40405949	-3.08647576	3.49363576
C	12.23143066	-2.57850705	4.24607124
C	13.18738439	-1.97964711	5.12713118
C	14.57915797	-2.08833644	4.85523693

C	12.75678238	-1.26724433	6.28228900
C	13.70294162	-0.68765819	7.12581797
C	15.09095031	-0.80187981	6.84411919
C	15.84231036	-0.09639769	7.89577721
C	14.91414346	0.45032997	8.82074999
C	15.52943119	-1.50390565	5.70720669
H	10.22143222	-5.38282458	-0.37411330
H	3.02235591	-12.65913611	-6.95818350
H	1.76290153	-17.17516968	-5.17629694
H	3.46300009	-18.95719439	-6.12728823
H	6.77782651	-16.58495638	-9.05773409
H	5.28637380	-20.22872228	-7.26547393
H	6.92695406	-19.05208179	-8.71651869
H	-1.86622868	-11.57122648	-3.31184000
H	0.53248769	-9.12128161	-5.94116521
H	-3.20813133	-9.54860488	-2.67367172
H	-0.80975750	-7.12393320	-5.31432444
H	-0.44688987	-6.30616093	-2.73285262
H	-4.42320031	-5.14477230	-4.00153965
H	12.54500258	2.00326770	9.05286534
H	11.64004191	1.15398721	7.78303219
H	13.13234974	2.03062679	7.37498838
H	12.66445529	-0.17246152	10.43378702
H	13.32035869	-1.65661516	9.70674490
H	11.75047570	-1.01829600	9.16802103
H	3.02238377	14.57581537	-8.25937898
H	3.58008067	13.21699265	-7.26170624
H	4.07926517	14.87587987	-6.86124643
H	0.57538171	13.83995201	-7.90041222
H	-0.05884188	13.62094047	-6.25373910
H	1.13561919	12.47788788	-6.90864743
H	-16.81810421	0.96831860	-5.91167612
H	-15.19187595	1.64592633	-5.69082172
H	-16.28102581	1.47900454	-4.29530702
H	-15.79831440	-1.12505605	-7.01557791
H	-14.55261459	-2.06783483	-6.16617900
H	-14.17097196	-0.44949817	-6.79659595
H	5.28639994	-14.53832019	-9.93680307
H	4.14449489	-13.33818440	-9.29765760
H	3.57798323	-14.97348074	-9.70674892
H	6.81589668	-14.16731593	-7.89527127
H	6.17035510	-14.34980339	-6.24861317
H	5.67340343	-12.96920038	-7.25317130
H	17.11747248	1.92719083	10.96397855
H	18.74163737	0.96707357	9.34470670

H	14.66379188	1.60410155	10.64174760
H	17.94063002	-0.33398012	7.36921923
H	16.58923674	-1.59684564	5.48284331
H	11.69207744	-1.18730663	6.48544608
H	14.89815273	-2.63431595	3.97153795
H	8.72744975	-3.15929113	3.83029602
H	11.91446328	-4.36301305	1.17239889
H	7.05660804	-4.18430685	2.29985084
H	0.43879157	-15.15524879	-4.55292661
H	0.47819472	18.78284630	-7.62695665
H	0.58910246	19.53829906	-5.26094622
H	1.10052303	16.44628866	-8.24297284
H	1.32746165	17.96817211	-3.46430248
H	2.16158347	15.94768680	-1.98268827
H	2.93007714	11.99173649	-4.96884265
H	2.96168596	13.78404602	-1.03442885
H	4.34145180	8.15077207	-3.21199251
H	4.43974585	9.99005714	0.69686215
H	5.16197654	6.01529829	-2.23309411
H	5.25648192	7.82916729	1.67839658
H	-19.47396375	-2.83156026	-4.16550588
H	-18.60938338	-3.57310231	-1.95520048
H	-18.08911673	-1.37815411	-5.64627124
H	-16.33904428	-2.87317178	-1.18509695
H	-13.91706758	-1.87271423	-0.85933385
H	-12.75162899	0.83567886	-4.91687555
H	-11.71161747	-0.71891142	-1.01906264
H	-7.77364887	1.28249140	-1.25888616
H	-8.80932394	2.89777444	-5.13043931
H	-5.56245755	2.41549961	-1.37166654
H	-6.57149279	4.02824641	-5.24265887
H	7.31589748	3.64985519	0.67823521
H	6.76443600	1.21104941	0.28545396
H	3.32865190	4.80980120	-0.55759126
H	-0.54798975	4.89895513	-1.89187387
H	-2.88492882	5.54505625	-2.61883445
H	-3.88247413	1.38593116	-3.30743872
H	5.77828556	-7.03819669	-0.70393615
H	6.79206217	-2.87388371	-0.07053854
H	3.44625535	-6.39208217	-1.44546801
H	-3.87017354	-2.70535187	-3.61548805
N	0.08431890	0.70771247	-2.01526738
N	0.10188002	-2.14538343	-2.23867074
N	1.47009979	-4.15398990	-1.93504015
N	2.81567017	-2.19984256	-1.33132792

N	2.79763875	0.65314788	-1.10643780
N	-1.79059263	-0.68177690	-2.75854132
N	1.42665966	2.66105232	-1.40150142
O	-4.73914319	4.01272660	-3.43184513
O	7.67374836	-5.49828407	-0.01790198
O	5.78489647	5.65350657	0.40134073
O	-2.93547744	-7.15946078	-3.59265094
Zn	1.45027685	-0.74598537	-1.67410809
C	4.11439724	-1.98843655	-0.87853146
N	4.69141325	-0.81018855	-0.59047750

d) H_2TOFPc4':

Atom	X	Y	Z
C	1.60937850	-13.72406876	-5.77030023
C	1.26601247	-15.01567638	-5.28370180
C	2.72816437	-13.58171849	-6.63937062
C	0.83652799	-12.58152989	-5.38808180
C	3.46802341	-14.70710424	-6.99873811
C	3.11566753	-15.99264612	-6.50665873
C	4.06539634	-16.97868537	-7.04849919
C	4.99938143	-16.29692531	-7.87257639
C	2.01291847	-16.14694447	-5.64782840
C	4.70102536	-14.79058830	-7.91154733
C	4.14945569	-18.37040958	-6.86589335
C	6.02062578	-17.00405154	-8.51675391
C	5.17876964	-19.07892928	-7.51649048
C	6.10784329	-18.40229642	-8.33584027
C	0.17727376	-11.59740631	-5.06298487
C	-0.59426218	-10.45447711	-4.67745200
C	-1.67108108	-10.58076209	-3.75999432
C	-0.30579283	-9.16897947	-5.20608372
C	-2.42269025	-9.46249473	-3.37628203
C	-2.10929975	-8.19877987	-3.90683297
C	-1.05695421	-8.04656680	-4.83046574
C	12.64703573	1.50999186	8.10774773
C	-2.50681015	-5.82327060	-3.35138877
C	-1.21589255	-5.52353196	-2.87117579
C	-0.92258367	-4.17187020	-2.66153175
C	-1.87283201	-3.15070287	-2.90609363
C	0.29288106	-3.49037605	-2.18037452
C	-1.20790268	-1.88525582	-2.56566936
C	-3.46930605	-4.81452071	-3.59701028
C	-3.15877485	-3.46263139	-3.37157910
C	12.80466906	-0.62203823	9.48742051

C	3.39570224	14.15451952	-7.21693837
C	2.53553730	-3.59552010	-1.41328631
C	3.76067282	-4.28934979	-1.04255543
C	0.97463235	13.44420480	-6.88858167
C	4.70974506	-3.30933669	-0.63653407
C	4.09672779	-5.65606779	-1.03747896
C	6.00265095	-3.66022583	-0.21794778
C	6.31550477	-5.02932619	-0.20685206
C	5.37977488	-6.01891389	-0.61395498
C	-15.91709988	0.95846519	-5.39689630
C	8.47438300	-4.83724444	0.98013383
C	-14.87926603	-1.08065359	-6.50944522
C	-1.26245770	0.47909251	-2.37456624
C	-1.90532564	1.78656775	-2.47329255
C	4.37344952	-14.31362825	-9.35477096
C	-0.95717024	2.76617772	-2.06366432
C	0.27273321	2.07333694	-1.70720273
C	-3.20085621	2.13778056	-2.88333476
C	-3.52336438	3.50447762	-2.86312371
C	-2.58502022	4.49433101	-2.46349924
C	5.88750262	-13.96368842	-7.34032139
C	-5.95065283	3.25774552	-3.24826651
C	-1.29425131	4.13253138	-2.06267707
C	16.78771673	1.52468232	10.00623255
C	17.69872363	0.98066211	9.07528271
C	2.51764341	1.96897524	-0.94676763
C	3.73480300	2.65086644	-0.47045902
C	4.68782081	1.63033868	-0.23420701
C	4.02215561	0.36479348	-0.57267071
C	15.39790635	1.32578408	9.85095522
C	4.02248603	4.00111506	-0.24429692
C	5.31761724	4.30163630	0.22396388
C	5.97009253	1.94120855	0.24209052
C	6.28062231	3.29295276	0.46808403
C	17.23618128	0.23116053	7.97570806
C	5.20528107	6.75147126	-0.07369100
C	13.48807804	0.23156867	8.38167147
C	-6.83565690	3.41118817	-4.32870810
C	-6.30245976	2.47533601	-2.13182315
C	-8.08015114	2.76709887	-4.29966516
C	-8.45665705	1.96024585	-3.19355702
C	-7.54640307	1.82983472	-2.11146777
C	-9.72637748	1.29918810	-3.16443776
C	-10.81248362	0.72674428	-3.12804035
C	-12.07998987	0.06276862	-3.09146657

C	-12.43724950	-0.73402300	-1.96886406
C	-12.99498274	0.19094967	-4.17480629
C	-14.22371109	-0.46475442	-4.11860754
C	-14.57045078	-1.25782589	-2.99163524
C	-15.91323746	-1.82112883	-3.21001539
C	-16.38819177	-1.37378381	-4.47101125
C	-13.67559692	-1.39255160	-1.91516304
C	-15.35357230	-0.47122841	-5.15992679
C	-16.69967323	-2.66161272	-2.40229314
C	-17.65082531	-1.76571153	-4.92905212
C	-17.97044450	-3.05397844	-2.86684670
C	-18.44348112	-2.61018336	-4.12047043
C	5.02096306	7.88571337	0.73598335
C	4.97569888	6.81210979	-1.46187247
C	4.59212615	9.08736971	0.15701499
C	4.33976164	9.17426294	-1.23795925
C	4.54289948	8.01640317	-2.03406463
C	3.89765039	10.40249482	-1.82637764
C	3.51752321	11.45968761	-2.32300345
C	3.07530037	12.68917253	-2.90743342
C	2.84957737	13.82992781	-2.08843662
C	2.85660154	12.78517766	-4.31113859
C	2.42615490	13.99217964	-4.85957031
C	2.20345343	15.12532614	-4.03170444
C	1.75850713	16.24456862	-4.87872681
C	1.71005795	15.79695262	-6.22532456
C	2.41555598	15.04373902	-2.64388578
C	2.12964283	14.32302671	-6.33030575
C	1.41179814	17.56645291	-4.54756912
C	1.31567188	16.66936820	-7.24566370
C	1.01465137	18.44178082	-5.57759425
C	0.96679582	17.99801273	-6.91668881
C	9.83465879	-4.88818969	0.63211289
C	8.05674161	-4.21599228	2.17254219
C	10.78666432	-4.30012364	1.47600100
C	10.39346144	-3.65149413	2.67653210
C	9.01332633	-3.62462301	3.00910947
C	11.36581633	-3.04320501	3.53357897
C	12.20228014	-2.51908196	4.26450078
C	13.16784801	-1.90150297	5.12183779
C	14.55548586	-1.99856649	4.82579616
C	12.75041548	-1.18229242	6.27760933
C	13.70539139	-0.58482346	7.09835578
C	15.08928049	-0.68756896	6.79272930
C	15.85196853	0.03571525	7.82393357

C	14.93488504	0.58160007	8.76032954
C	15.51469709	-1.39609836	5.65486455
H	10.12463342	-5.38766115	-0.28695530
H	2.98611614	-12.59235622	-7.00799242
H	1.73549483	-17.12651024	-5.26619882
H	3.43677521	-18.89714309	-6.23554397
H	6.74336061	-16.49405114	-9.15003318
H	5.26031239	-20.15574699	-7.38779145
H	6.89684170	-18.96372337	-8.83086342
H	-1.90755156	-11.55908043	-3.35162056
H	0.50662007	-9.05898014	-5.91876710
H	-3.24844174	-9.54779222	-2.67689816
H	-0.83412713	-7.07275964	-5.25548921
H	-0.48351376	-6.29489443	-2.66056240
H	-4.44695795	-5.11859313	-3.95731236
H	12.55556348	2.11328636	9.02019547
H	11.63546966	1.24261362	7.77613532
H	13.11117265	2.12953390	7.33145476
H	12.72042992	-0.04812322	10.41924984
H	13.37899555	-1.53250496	9.69508691
H	11.79328164	-0.91571743	9.17811318
H	3.18487723	14.45816720	-8.25048472
H	3.71981722	13.10590078	-7.23162495
H	4.22496538	14.76542825	-6.84140805
H	0.72917929	13.73975061	-7.91681884
H	0.07062671	13.54497639	-6.27662977
H	1.26617618	12.38608631	-6.90095097
H	-16.76517479	0.92686894	-6.09301701
H	-15.14737495	1.60959110	-5.83083027
H	-16.25940744	1.40903267	-4.45790491
H	-15.71454114	-1.13849009	-7.21919394
H	-14.47796827	-2.09084701	-6.36714178
H	-14.09502989	-0.45850302	-6.95955211
H	5.24478436	-14.44303151	-10.00958944
H	4.10286103	-13.24988731	-9.35739986
H	3.53761361	-14.88251412	-9.77861452
H	6.77887597	-14.08604367	-7.96897211
H	6.13810715	-14.28391797	-6.32220839
H	5.63592561	-12.89561315	-7.31350147
H	17.16083363	2.10126831	10.84947914
H	18.76559703	1.14295939	9.20994256
H	14.70526223	1.74975592	10.57483472
H	17.94279361	-0.18505782	7.26145382
H	16.57122154	-1.48035517	5.41240067
H	11.68874188	-1.11129808	6.49914531

H	14.86432211	-2.54993155	3.94182866
H	8.69907616	-3.14359428	3.93088955
H	11.83974652	-4.33420234	1.21228765
H	7.00644521	-4.20294588	2.44742824
H	0.41025956	-15.11407605	-4.62131353
H	0.65846654	18.68619584	-7.70033708
H	0.74240320	19.46791901	-5.34170576
H	1.27462847	16.33903581	-8.28135348
H	1.44726835	17.91403272	-3.51769405
H	2.24824968	15.90525260	-2.00204617
H	3.03080097	11.91062526	-4.93249357
H	3.01958460	13.74700388	-1.01839502
H	4.36579482	8.07106596	-3.10442052
H	4.44656353	9.96673778	0.77774951
H	5.14136984	5.93833464	-2.08463117
H	5.21873098	7.80869914	1.80051044
H	-19.42681617	-2.92174553	-4.46505352
H	-18.59359548	-3.70360683	-2.25664823
H	-18.02698852	-1.43038686	-5.89320699
H	-16.34048830	-3.00623051	-1.43537049
H	-13.93161282	-1.99749225	-1.04878190
H	-12.71636271	0.80077378	-5.03021313
H	-11.73204382	-0.82597019	-1.14728358
H	-7.82615543	1.22570792	-1.25323638
H	-8.76878537	2.88086242	-5.13188973
H	-5.62322589	2.38058555	-1.29003168
H	-6.53911354	4.03265159	-5.16781639
H	7.25570262	3.59635869	0.83585159
H	6.69989796	1.15931843	0.42668021
H	3.27581006	4.76910530	-0.41327039
H	-0.56942279	4.87895454	-1.75420731
H	-2.90307792	5.53183899	-2.48180492
H	-3.90540378	1.37933201	-3.20566161
H	5.69517764	-7.05702736	-0.58629461
H	6.71693033	-2.89785259	0.07159166
H	3.37399063	-6.40272371	-1.35026885
H	-3.88915039	-2.68099640	-3.55492844
N	0.03113661	0.71475796	-1.91210843
N	0.09217637	-2.11880317	-2.13476684
N	1.41203520	-4.16179944	-1.84815528
N	2.78106541	-2.23580562	-1.22060697
N	2.72036426	0.59791876	-0.99847320
N	-1.82200162	-0.69013999	-2.67134960
N	1.39606545	2.63968648	-1.27205526
O	-4.75789004	4.01222357	-3.30153511

O	7.58317136	-5.53102749	0.13194949
O	5.72393276	5.60641536	0.56486720
O	-2.96597852	-7.14132439	-3.53941359
C	4.07328176	-2.00035954	-0.75426174
N	4.63495333	-0.83067979	-0.46340427
H	2.09667923	-1.50292467	-1.39683845
H	0.71761955	-0.01773510	-1.74246850

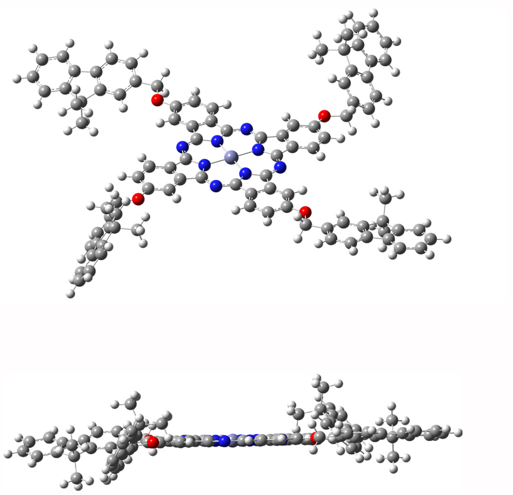

ZnTOFPc2’

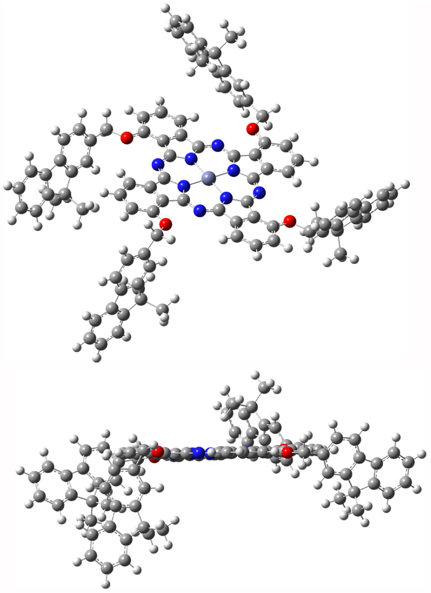

ZnTOFPc3’

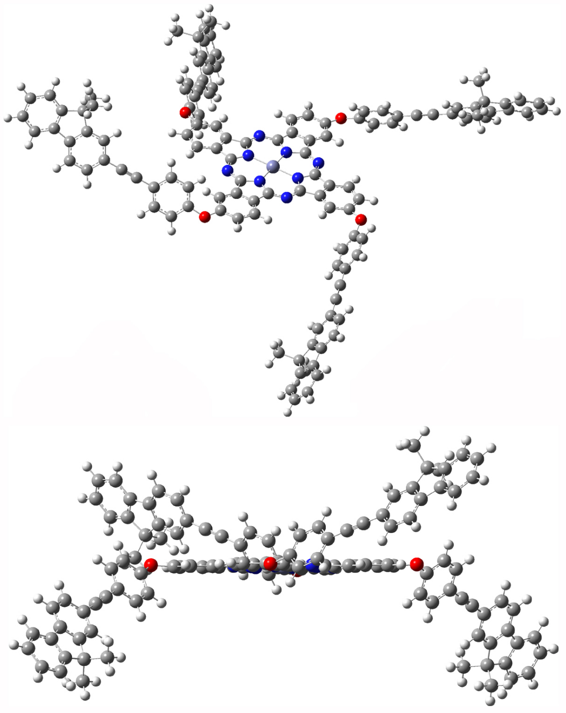

ZnTOFPc4’

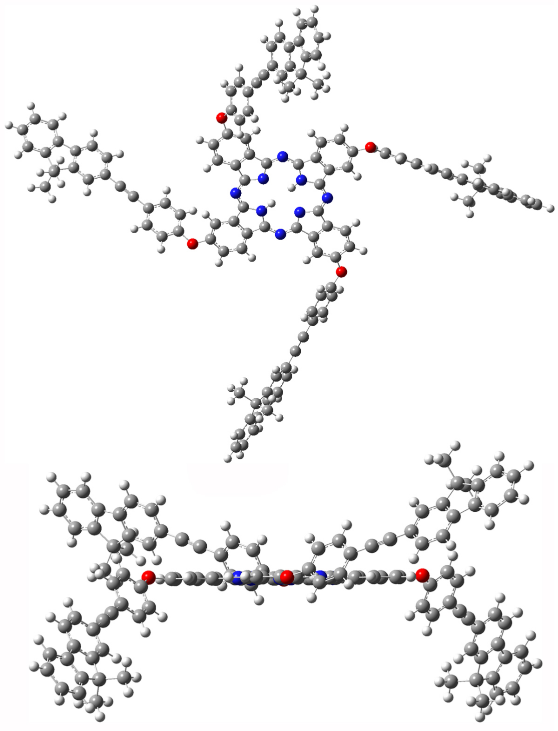

H_2TOFPc4’

Figure S27: Optimized geometries of **ZnTOFPc2’-4’** and **H_2TOFPc4’** (view from above and side view).

Table S2. Experimental *vs.* computed (B3LYP / 6-31G* or LANL2DZ) values (nm). Energy and composition of the first singlet excited states (wavelength, oscillator strength *f*, transition percentage).

Model Cpnd	Experimental value (mixture of stereoisomers) λ_{max} [ε] [a]	Calculated [b] λ_{max} [f [c]]	Composition	Major Assignment
**ZnTOFPc2'**	677 [215]	620 [0.55]	H→L (94%)	$\pi^*\leftarrow\pi$ (Q Band)
	/	619 [0.55]	H→L+1 (94%)	$\pi^*\leftarrow\pi$ (Q Band)
	/	427 [0.04]	H-5→L (43%)	$\pi^*\leftarrow\pi$ (B Band)
	/	427 [0.06]	H-5→L (23%), H-5→L+1 (29%)	$\pi^*\leftarrow\pi$ (B Band)
	351 [94]	386 [0.24]	H-8→L+1 (79%)	$\pi^*\leftarrow\pi$ (B Band)
	/	338 [0.50]	H-24→L (21%), H-21→L+1 (18%)	$\pi^*\leftarrow\pi$ (N Band[d])
	/	337 [0.22]	H-24→L (33%), H-10→L+1 (29%)	$\pi^*\leftarrow\pi$
	306 [61]	336 [0.60]	H-21→L (20%), H-21→L+1 (19%)	$\pi^*\leftarrow\pi$
	/	336 [0.12]	H-24→L (82%)	$\pi^*\leftarrow\pi$
	276 [107]	/	/	$\pi^*_{Flu}\leftarrow\pi_{Flu}$
**ZnTOFPc3'**	698 [270]	632 [0.43]	H→L (86%)	$\pi^*\leftarrow\pi$ (Q Band)
	/	630 [0.50]	H→L+1 (86%)	$\pi^*\leftarrow\pi$ (Q Band)
	/	418 [0.06]	H-5→L+1(36%)	$\pi^*\leftarrow\pi$ (B Band)
	353 [51]	368 [0.18]	H-10→L (32%)	$\pi^*\leftarrow\pi$ (B Band)
	307 [86]	335 [0.19]	H-21→L (35%), H-19→L (32%)	$\pi^*_{Pc}\leftarrow\pi_{Flu}$
	/	333 [0.18]	H-21→L+1(35%), H-19→L+1(32%)	$\pi^*_{Pc}\leftarrow\pi_{Flu}$
	270 [100]	/	/	$\pi^*_{Flu}\leftarrow\pi_{Flu}$
**ZnTOFPc4'**	675 [291]	630 [0.50]	H→L (92%)	$\pi^*\leftarrow\pi$ (Q Band)
	/	630 [0.50]	H→L+1 (92%)	$\pi^*\leftarrow\pi$ (Q Band)
	/	555 [0.13]	H-4→L (39%), H-4→L+1 (56%)	$\pi^*_{Pc}\leftarrow\pi_{Flu}$
	/	410 [0.27]	H-8→L (88%)	$\pi^*\leftarrow\pi$ (B Band)
	/	410 [0.27]	H-8→L+1 (88%)	$\pi^*\leftarrow\pi$ (B Band)
	344 [253]	366 [0.62]	H→L+4 (85%)	$\pi^*_{Flu}\leftarrow\pi_{Pc}$
	327 (sh)	367 [0.53]	H→L+3 (55%)	$\pi^*_{Flu}\leftarrow\pi_{Pc}$
	/	359 [0.27]	H-16→L+1 (45%)	$\pi^*\leftarrow\pi$ (N Band[d])
	/	349 [0.35]	H-11→L (31%)	$\pi^*\leftarrow\pi$ (B Band)
**H_2TOFPc4'**	699 [175]	647 [0.48]	H→L (85%),	$\pi^*\leftarrow\pi$ (Q Band)
	665 [158]	631 [0.44]	H→L+1 (87%)	$\pi^*\leftarrow\pi$ (Q Band)
	/	569 [0.22]	H-4→L (49%), H-2→L+1 (45%)	$\pi^*_{Pc}\leftarrow\pi_{Flu}$
	/	465 [0.06]	H-6→L (84%)	$\pi^*_{Pc}\leftarrow\pi_{Flu}$
	/	413 [0.40]	H-8→L (89%)	$\pi^*\leftarrow\pi$ (B Band)
	/	404 [0.30]	H-8→L+1 (83%)	$\pi^*\leftarrow\pi$ (B Band)
	344 [261]	368 [0.72]	H→L+3 (68%)	$\pi^*_{Flu}\leftarrow\pi_{Pc}$
	324 (sh)	363 [0.85]	H-16→L (10%), H-15→L (11%),	$\pi^*\leftarrow\pi$ (N Band[d])
	/		H-13→L (10%)	
	/	362 [0.47]	H→L+5 (43%)	$\pi^*_{Flu}\leftarrow\pi_{Pc}$
	/	353 [0.91]	H-21→L (34%)	$\pi^*\leftarrow\pi$

a. Experimental absorption (nm) and extinction coefficients (ε) in 10^3 $M^{-1}.cm^{-1}$. *b*. in nm. *c*. Computed oscillator strength. *d* Tentative assignment.

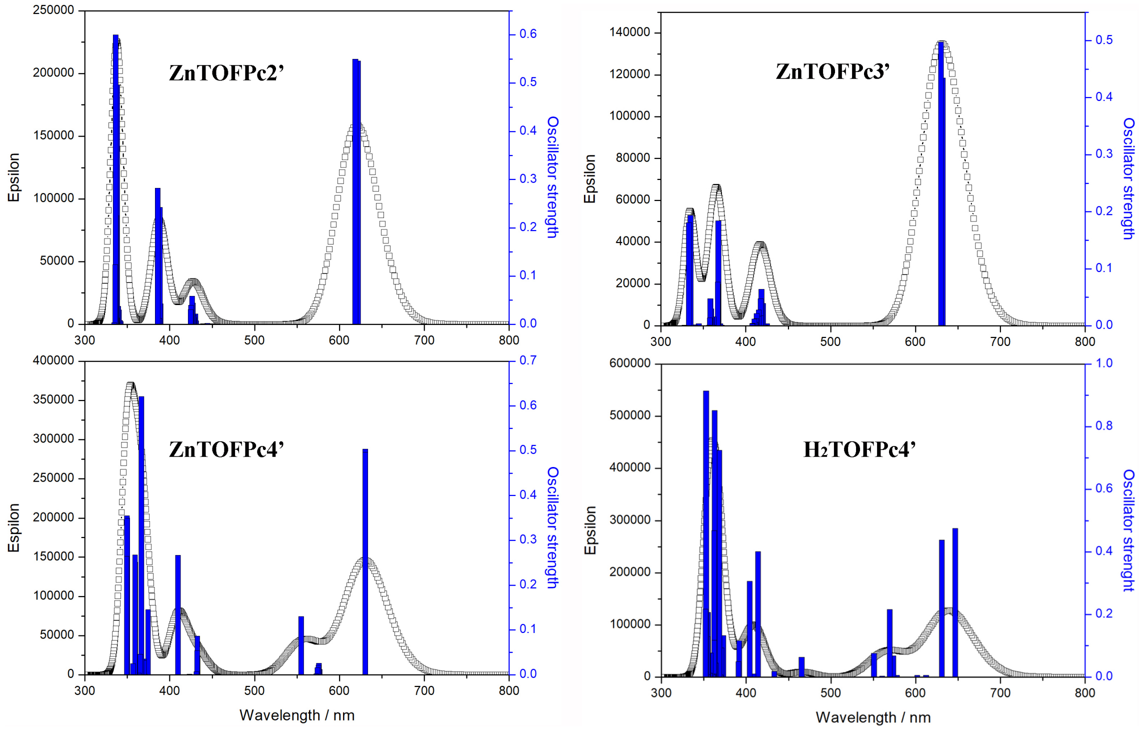

Figure S28: UV-visible absorption spectra simulated from the vertical excitation calculated by TD-DFT (40 states).
